# Reactivity of the phosphaethynolate anion with stabilized carbocations: mechanistic studies and synthetic applications†‡

**DOI:** 10.1039/d4sc03518f

**Published:** 2024-08-08

**Authors:** Thi Hong Van Nguyen, Saloua Chelli, Sonia Mallet-Ladeira, Martin Breugst, Sami Lakhdar

**Affiliations:** a CNRS, Université Paul Sabatier, Laboratoire Hetérochimie Fondamentale et Appliquée (LHFA, UMR5069) 118 Route de Narbonne 31062 Cedex 09 Toulouse France sami.lakhdar@univ-tlse3.fr; b Institut de Chimie de Toulouse (FR 2599) 118 Route de Narbonne 31062 Cedex 09 Toulouse France; c Institut für Chemie, Technische Universität Chemnitz 09111 Chemnitz Germany martin.breugst@chemie.tu-chemnitz.de

## Abstract

The reactivity of sodium phosphaethynolate Na(OCP) towards various Mayr's reference electrophiles was investigated using conventional UV-visible and laser-flash photolysis techniques. The kinetic data, along with density functional theory (DFT) calculations, enabled the first experimental quantification of the phosphorus nucleophilicity of [OCP]^−^. Product studies of these reactions demonstrate the formation of secondary as well as tertiary phosphines. The mechanism of this unprecedented phosphorus-atom transfer reaction is thoroughly discussed, with key intermediates successfully isolated and characterized. Importantly, some bulky secondary phosphine oxides synthesized using this approach, have demonstrated high efficiency as ligands in the Suzuki coupling reaction.

## Abstract 2

Kinetics and computational studies enabled understanding the ambident reactivity of the sodium phosphaethynolate anion and have led the discovery of new reactivity with stabilized carbocations.

## Graphical abstract

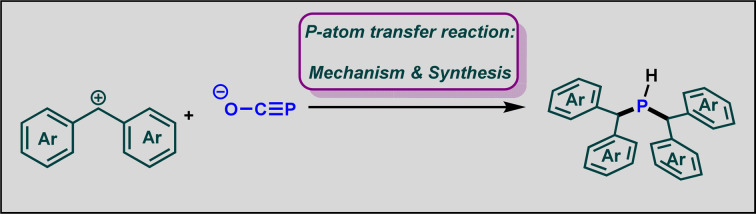

## Introduction

**Scheme 1:**
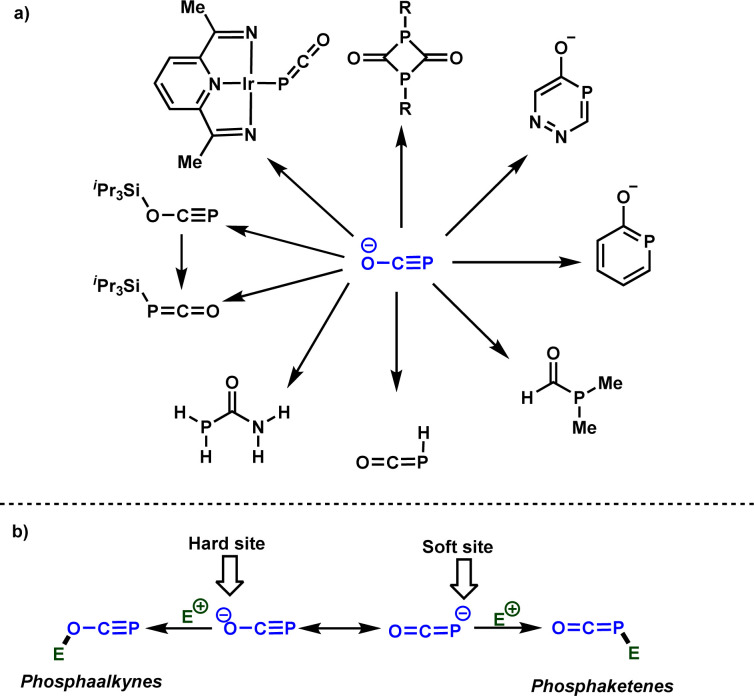
(a) Selected examples of the use of [OCP]^−^ for the synthesis of organophosphorus molecules, (b) ambident reactivity of [OCP]^−^ with electrophiles.

The exploration of practical, simple, robust, and sustainable approaches for forming carbon-phosphorus bonds constitutes a vibrant research domain for both academia and industry.^1^ This significance arises from the importance of organophosphorus compounds in many areas, spanning from catalysis to medicinal chemistry and materials sciences.^2^ Typically, PCl_3_ serves as a common starting material from which the majority of organophosphorus molecules can be synthesized. However, the generation of HCl as a side product has spurred efforts to identify alternative phosphorus precursors that are easily accessible and manufactured on a large scale.^3^ In this context, phosphinates and related derivatives,^4^ as well as white phosphorus (P_4_)^5^ have garnered attention in recent decades as PCl_3_ surrogates, given their potential to be converted into valuable organophosphorus molecules. Phosphaethynolate anion, denoted as [OCP]^−^, the phosphorus analog of well-studied cyanate anion, has emerged recently as a potential phosphorus precursor.^6^Scheme 1a summarizes selected examples of the use of [OCP]^−^ for the synthesis of interesting organophosphorus molecules. From the reactivity perspective, [OCP]^−^ possesses two nucleophilic centers (oxygen and phosphorus) that facilitate the formation of phosphaalkynes^7^ or phosphaketenes,^8^ serving as key intermediates for the synthesis of organic and organometallic phosphorus compounds (Scheme 1b).

Although the synthesis of [OCP]^−^ was initially reported nearly thirty years ago by Becker *et al.*,^9^ its utilization in inorganic and organic chemistry faced limited development during that period, likely attributed to challenges in reproducing its synthesis on a large scale and the sensitivity of the anion to moisture and air. A noteworthy resurgence in the chemistry of the phosphaethynolate anion has occurred in the last decade, driven by seminal contributions of Grützmacher and Goicoechea groups.^10^ These reports independently presented efficient procedures for synthesizing stable phosphaethynolate anion, overcoming previous limitations. Consequently, [OCP]^−^ has emerged as an appealing phosphorus transfer agent, demonstrating efficiency in the synthesis of numerous organophosphorus molecules and phosphorus-based transition metal complexes.^11^

It is obvious that the rational design of new reactions involving [OCP]^−^ as a nucleophile necessitates a comprehensive understanding of its ambident reactivity. In 2014, Grützmacher and Benkő elucidated this ambident reactivity by reacting [OCP]^−^ with iPr_3_Si–OTf, yielding the phosphaalkyne and phosphaketene, which were fully characterized (Scheme 1a).^12^ It was concluded that while the oxygen attack is kinetically controlled, the phosphorus attack is thermodynamically controlled. Very recently, the Benkő's group employed Marcus theory to rationalize the ambident reactivity of [OCP]^−^ towards carbon electrophiles. Their results indicated a lower intrinsic barrier for the oxygen attack, highlighting the kinetic preference of oxygen attacks.^13^

Building upon these insights and drawing inspiration from precedent contributions by Mayr and coworkers,^14^ which revealed that the ambident reactivity of various nucleophiles does not follow the well-established Hard and Soft Acids and Bases theory (HSAB),^15^ we embarked on investigating the reactivity of [OCP]^−^ towards Mayr's reference electrophiles. The objective is to gain deeper insights into the factors controlling the reactivity of this anion, which is crucial for unlocking its synthetic potential (Table 1).

**Table 1:** Reference electrophiles employed for the determination of the nucleophilicity parameters of Na(OCP), their electrophilicity

Electrophile		Electrophilicity, *E*
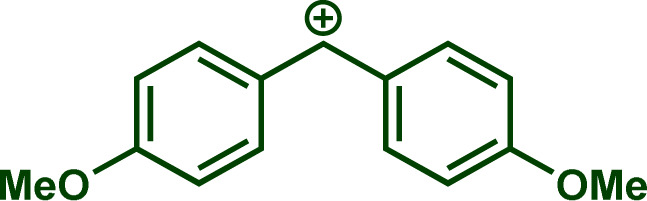	1a	0^a^
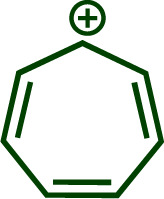	1b	−3.72^b^
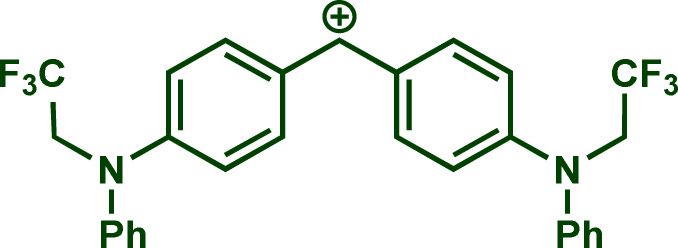	1c	−3.85^a^
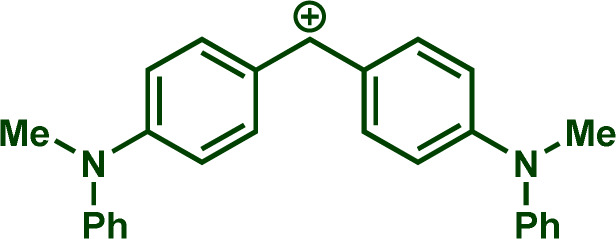	1d	−5.89^a^
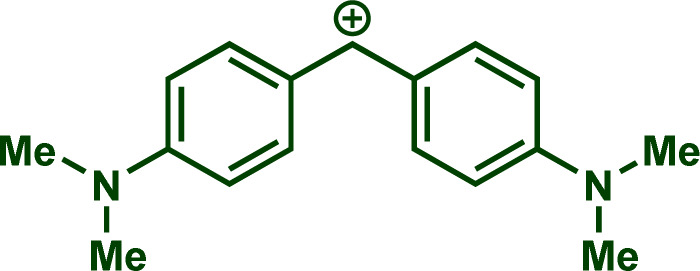	1e	−7.02^a^
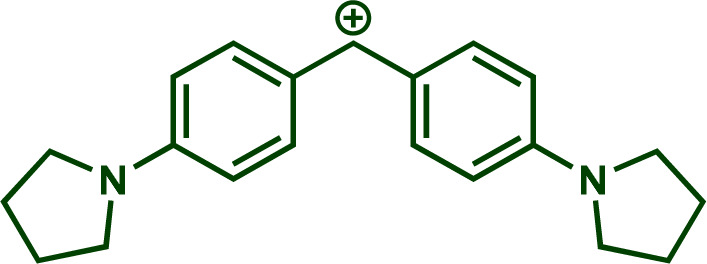	1f	−7.69^a^
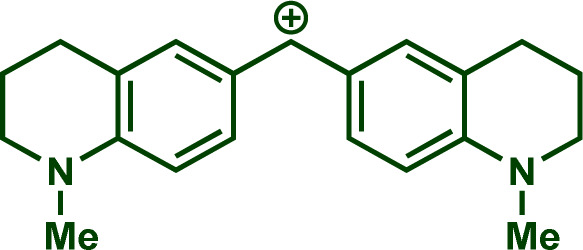	1g	−8.22^a^
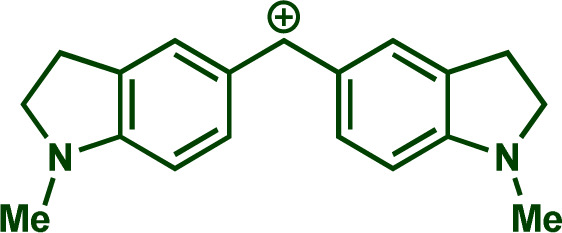	1h	−8.76^a^
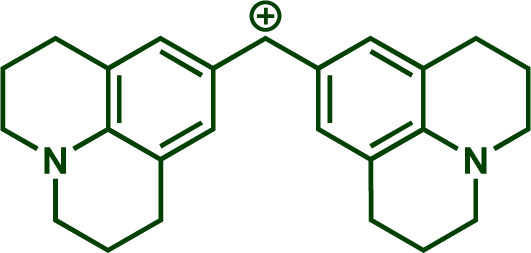	1i	−9.45^a^
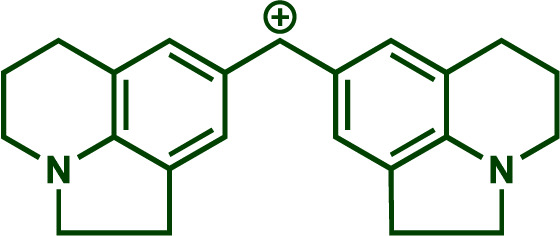	1j	−10.04^a^
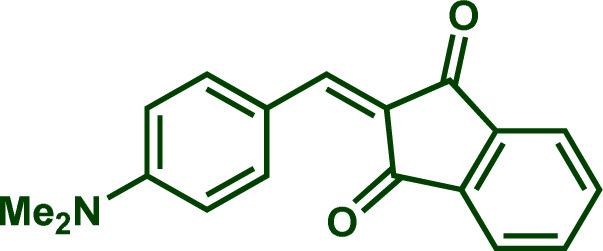	1k	−13.56^a^
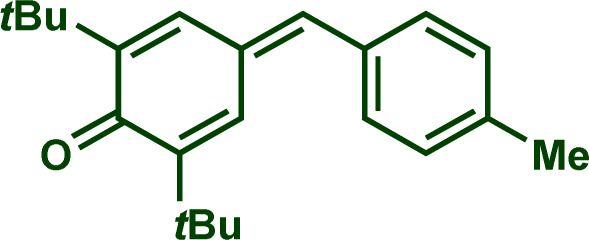	1l	−15.83^a^
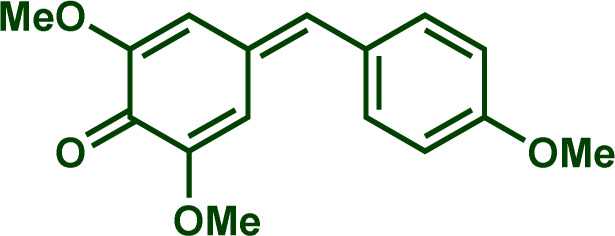	1m	−16.38^a^
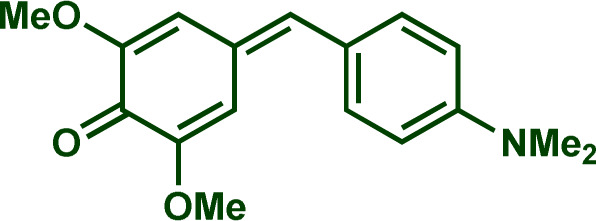	1n	−17.18^a^

a Taken from ref. 16.

b Taken from ref. 17.

In previous investigations, Mayr *et al.* have shown that numerous nucleophile–electrophile combinations can simply be described by the three parameters eqn (1), where *k*_2_ measures the second-order rate constant of the reaction of an electrophile with a nucleophile, *N* and *s*_N_ are nucleophilicity parameters, and *E* is the electrophilicity parameter.^18^

1 log *k*_2_ (20 °C) = *s*_N_(*E* + *N*)

**Scheme 2:**
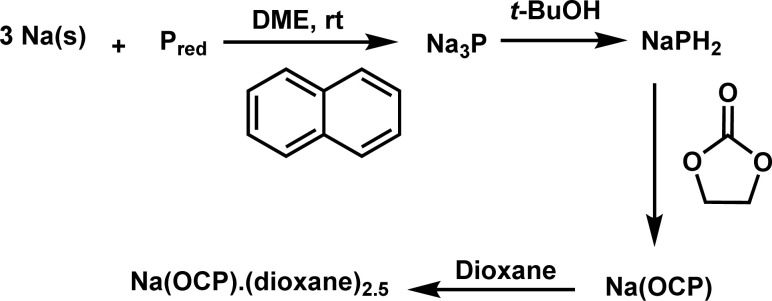
Synthesis of sodium phosphaethynolate.

Our investigation commences with the synthesis of sodium phosphaethynolate using a protocol outlined in Scheme 2. This method, previously described by Grützmacher,^12^ utilizes inexpensive precursors (sodium, red phosphorus, *t*BuOH, ethylene carbonate).^12^ The target compound, Na(dioxane)_2.5_(OCP) was successfully obtained on a gram scale. As ion pairing should not be important under the low concentrations used in the kinetic studies, [Na(OCP)](dioxane)_2.5_ will be referred to as Na(OCP) throughout this article.

The reaction of Na(OCP) with different reference electrophiles were carried out in acetonitrile at 20 °C, under pseudo-first-order conditions, by using at least 10 equivalents of the nucleophile with respect to the electrophile. Rates of those reactions were determined either by studying the kinetics of laser-flash photolytically generated benzhydrylium ions or conventional UV-visible spectrophotometry using stable quinone methides or benzhydrylium ions.

**Fig. 1:**
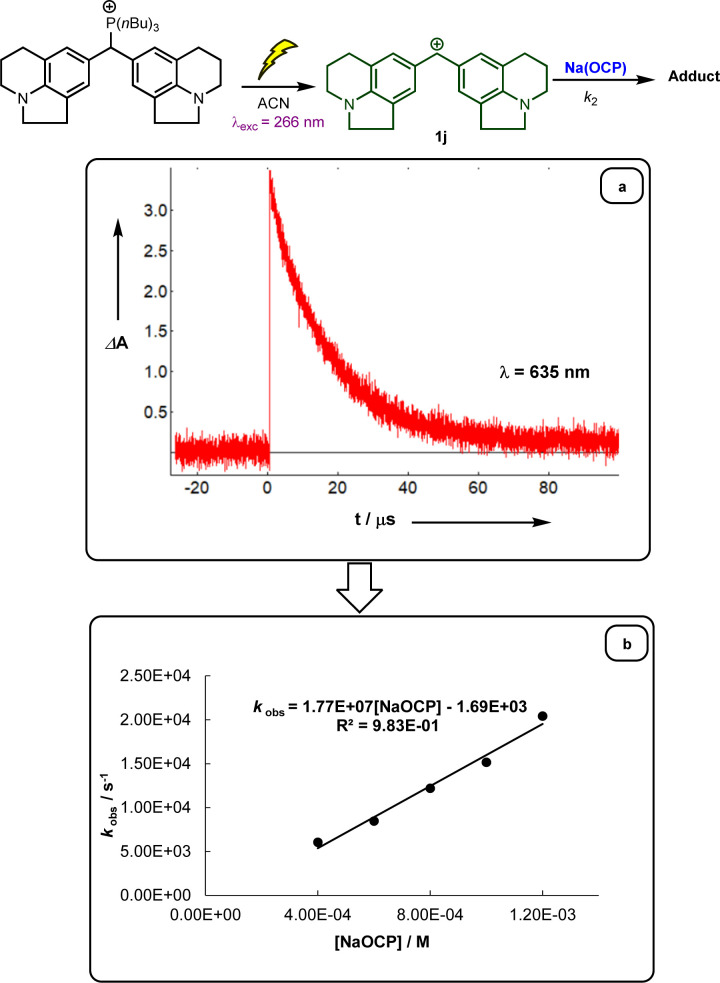
(a) Decay of the absorbance of carbocation 1j obtained after irradiation of a 3.07 × 10^−5^ mol L^−1^ solution of the phosphonium salt 1j-P(*n*Bu)_3_ in acetonitrile in the presence of Na(OCP). (b) Plot of the pseudo-first-order rate constants *k*_obs_ (s^−1^) *versus* the concentration of Na(OCP).

Following previous investigations by Mayr *et al.*,^19^ the carbocation 1j was generated photolytically upon irradiation of the corresponding phosphonium salt with a 7 ns laser pulse at 266 nm (see ESI‡). In the presence of a large excess of Na(OCP), one can follow the monoexponentional decays of the absorbance of 1j (*λ*_max_ = 635 nm), from which the rate constants *k*_obs_ (s^−1^) are obtained (Fig. 1a). Interestingly, plots of *k*_obs_*versus* the Na(OCP) concentrations gave linear correlations (Fig. 1b), and the resulting slopes yielded the second-order rate constants *k*_2_ (L mol^−1^ s^−1^) which are listed in Table 2. To investigate the role of the counterion on the reactivity of Na(OCP) with benzhydrylium ions, reactions of the former with the carbocation 1n were studied in the presence of the crown ether 15-crown-5. However, under these conditions, only a very small change in the second-order rate constant was noticed, indicating that the counterion does not play a crucial role in the reactivity of [OCP]^−^ towards carbocations (see ESI‡). The effect of dioxane on the reactivity has also been addressed, and we found that the reaction of dioxane-free Na(OCP) with 1n react similarly as [Na(OCP)](dioxane)_2.5_.

**Table 2:** Second-order rate constants for the reaction of [OCP^−^] with reference electrophile in acetonitrile at 20 °C

Electrophile	*k*_2_ (M^−1^ s^−1^)
1a	—a
1b	—a
1c	2.52 × 10^9^
1d	2.18 × 10^9^
1e	—
1f	1.04 × 10^8^
1g	9.67 × 10^7^
1h	2.23 × 10^8^
1i	1.08 × 10^8^
1j	1.77 × 10^7^
1k	1.74 × 10^4^
1l	2.98 × 10^2^
1m	2.17 × 10^2^
1n	3.71 × 10^1^

a Very fast reaction.

**Fig. 2:**
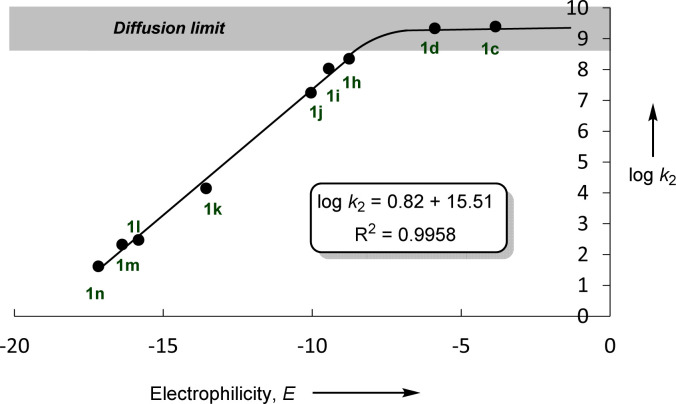
Plot of log *k*_2_ for the reactions of sodium phosphaethynolate Na(OCP) with reference electrophiles 1, in acetonitrile at 20 °C *versus* their electrophilicity parameters *E*.

In accordance with eqn (1), Fig. 2 shows that the second-order rate constants *k*_2_ correlate linearly with the electrophilicity parameters *E* of the reference electrophiles 1. The flattening of the curve at *k*_2_ ≈ 2.5 × 10^9^ L mol^−1^ s^−1^ is obviously due to diffusion control, which is in agreement with previous observations by Mayr *et al.* for the reactions of other benzhydrylium ions with other nucleophiles.^19*b*^ The nucleophilicity parameters (*N* = 19.02 and *s*_N_ = 0.82) of [OCP]^−^ were derived from the linear part of the curve (*i.e.*, reactions with 1h–1n in Fig. 2).

It should be noted that the measured second-order rate constants (Table 2) could not be attributed to a single electron transfer mechanism, as the oxidation of Na(OCP) is known to yield the heterobicyclic dianion, (P_4_C_4_O_4_)^2−^. This intermediate was not observed by ^31^P NMR when we investigated the reaction of Na(OCP) with various carbocations 1.

It is important to emphasize the excellent linearity observed in the correlation (log *k*_2_*vs. E*), indicating that the rate-determining step does not change throughout this reaction series. This implies that the same nucleophile terminus center attacks all electrophiles.

**Scheme 3:**
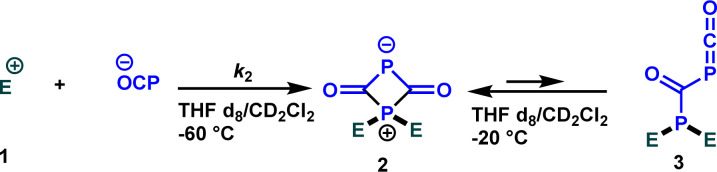
Reactions of Na(OCP) with carbocations 1b and 1e.

The next critical step involves determining whether the identified nucleophilicity parameters align with the attack on oxygen or phosphorus. To elucidate this aspect, we investigated the reaction outcomes of Na(OCP) with both the highly reactive electrophile 1b as well as the stabilized carbocation 1e (Scheme 3).

When Na(OCP) (1.2 equivalents) reacted with one equivalent of carbocation 1e in a dichloromethane/THF (1 : 1) mixture at room temperature, a complex ^31^P NMR spectrum was obtained. However, when the same reaction was carried out at low temperature (−60 °C), an immediate disappearance of the Na(OCP) (*δ*^31^P{^1^H} = −394.1 ppm) was observed within five minutes and a new species bearing two phosphorus atoms appeared (*δ*^31^P{^1^H} = 341.8 ppm (d, ^2^*J*_PP_ = 36.7 Hz) and 121.2 ppm (d, ^2^*J*_PP_ = 36.7 Hz)). ^1^H, ^13^C and 2D NMR experiments indicated the exclusive formation of the zwitterion 2. The same intermediate was also detected for other electrophiles 1 (for more information, see ESI‡). When the temperature was raised to −20 °C, the zwitterion 2 coexists with the phosphaketene adduct 3 (*δ*^31^P{^1^H} = −276.2 ppm (d, ^2^*J*_PP_ = 167.7 Hz) and 21.1 ppm (d, ^2^*J*_PP_ = 167.8 Hz)) in 5 to 1 ratio (2/3).

Grützmacher, Stephan, and co-authors reported the formation of structurally analogous complexes when they studied the reactions of the phosphaethynolate anion with a variety of boranes as Lewis acids.^20^

**Scheme 4:**
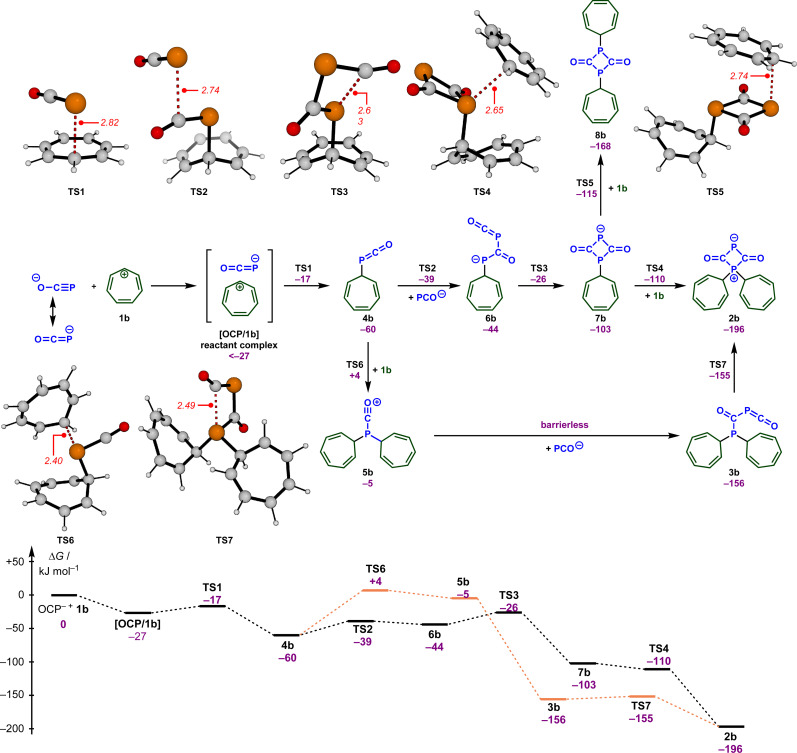
Calculated reaction free energies (in kJ mol^−1^) and selected transition states (bond lengths in Å) for the proposed reaction mechanism for the formation of the zwitterion 2b and the phosphaketene 3b.

To understand how these intermediates 2 and 3 were formed and to support the analysis of the kinetic data, we analyzed putative reaction mechanisms for the combination of free [OCP]^−^ with the tropylium cation 1b using density functional theory (RI-DSD-PBEP86-D3(BJ)/def2-QZVPP/SMD(THF)//M06-2X/6-31+G(d,p)/SMD(THF)). These results are summarized together with selected transition state structures in Scheme 4. Our calculations predict that [OCP]^−^ and 1b initially form a reactant complex [OCP/1b] that is more stable than the separated reactants. This complex is most likely held together *via* coulombic interactions. However, due to the large number of potential conformers of the reactant complex, the energetic value for this complex in Scheme 4 corresponds to the optimized structure at the end of the corresponding IRC calculation. Therefore, it is not unlikely that there are more stable bimolecular complexes. Within this complex, the OCP anion undergoes a rapid reaction with the tropylium ion (TS1, Δ*G*^‡^ = 10 kJ mol^−1^) and forms the P-alkylated intermediate 4b. This is in perfect agreement with the high nucleophilicity (see above) and electrophilicity (*E* = −3.72)^17^ of the tropylium ion. The alternate reactivity at the oxygen atom of [OCP]^−^ (not shown in Scheme 4) leads to an *O*-alkylated compound that lies 101 kJ mol^−1^ above intermediate 5b.

In the next steps, 4b is transformed into the experimentally observed intermediate 2b. This requires the reaction with another [OCP]^−^ and a second tropylium cation. According to our DFT calculations, the formal [2 + 2] cycloaddition between [OCP]^−^ and 4b proceeds in a stepwise fashion and occurs very quickly (TS2, TS3, Δ*G*^‡^ = 21 and 34 kJ mol^−1^) and forms the diphosphetanedione 6b. In this sequence, another [OCP]^−^ will initially attack the electrophilic carbon atom within phosphaketene 4b. This is followed by a ring-closing reaction to yield the diphosphetanedione anion 7b. Alkylation of 7b with a second equivalent of the tropylium cation again proceeds very rapidly and can occur at both phosphorus atoms through TS4 or TS5. While negative barriers were calculated on the RI-DSD-PBEP86 potential energy surface in both cases, small barriers were determined on the M06-2X surface. Eventually, the zwitterion 2b is formed with a high thermodynamic driving force (Δ*G* = −196 kJ mol^−1^) as the more stable species.

Alternatively, intermediate 4b can first react with the tropylium ion through TS6, which requires a slightly larger barrier of 64 kJ mol^−1^. No transition states could be located on the potential energy surface for the reaction of 5b with the OCP anion and all potential energy surface scans resulted in a barrierless addition. This indicates that this step will proceed very rapidly under the reaction conditions. Cyclization of phosphaketene 3b finally results in the zwitterion 2b again without a significant barrier. In agreement with the experimental observations (Scheme 3), the computational investigations also indicate that zwitterion 2b is more stable than the phosphaketene 3b, however, the thermodynamic difference seems to be substantially overestimated in the calculations. To better understand this deviation, we first calculated the thermodynamic differences for other substituents on the phosphorus atom (see the ESI for details‡). Regardless of the substituent, a comparable strong preference for the zwitterions was observed in all cases. Similarly, different computational methods (*e.g.*, DLPNO-CCSD(T), B2GP-PLYP, M06-2X, ω97X-V) also resulted in almost identical energy differences in favor of the zwitterion. Finally, we realized that solvation seems to be an important aspect. In the gas phase, both structures 2b and 3b are almost isoenergetic, and with increasing polarity, the zwitterion 2b substantially benefits from solvation, which then leads to an overestimation. Thus, the overestimation can be traced back to issues arising from the charge separation within the zwitterions.

**Scheme 5:**
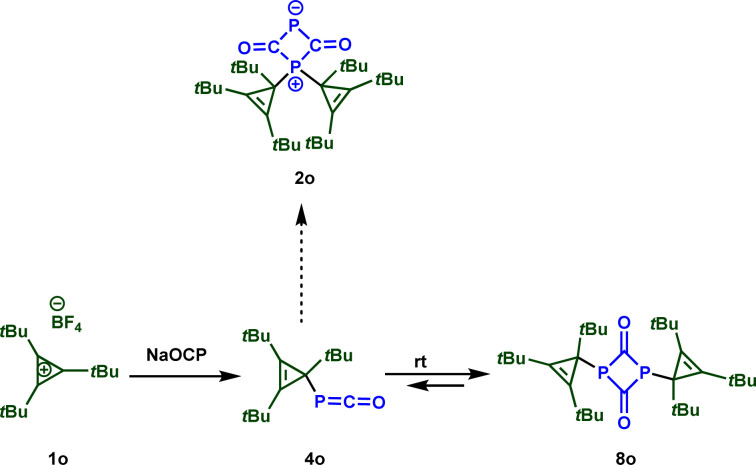
Reaction of sodium phosphaethynolate with 1,2,3-tris-*tert*-butylcyclopropenium 1o.^11*j*^

These results seem to contradict earlier observations by Slootweg and coworkers, who reported a different product for the reaction of sodium phosphaethynolate with 1,2,3-tris-*tert*-butylcyclopropenium tetrafluoroborate 1o (Scheme 5).^11*j*^ Indeed, while the first step of the reaction leads to a phosphaketene, similar to the reaction with our reference electrophiles 1, a fast [2 + 2] cycloaddition yields bis(cyclo-propenyl)diphosphetanedione 8o. The different reactivity of these electrophiles is probably caused by the steric demand within 1o. While the 1,3-disubstituted diphosphetandione 8b is thermodynamically less stable than the zwitterion 2b (see Scheme 4), the situation reverts for the electrophile 1o. According to our calculations, 8o is substantially more stable than 2o (ΔΔ*G* = −48 kJ mol^−1^) (Scheme 5).

**Fig. 3:**
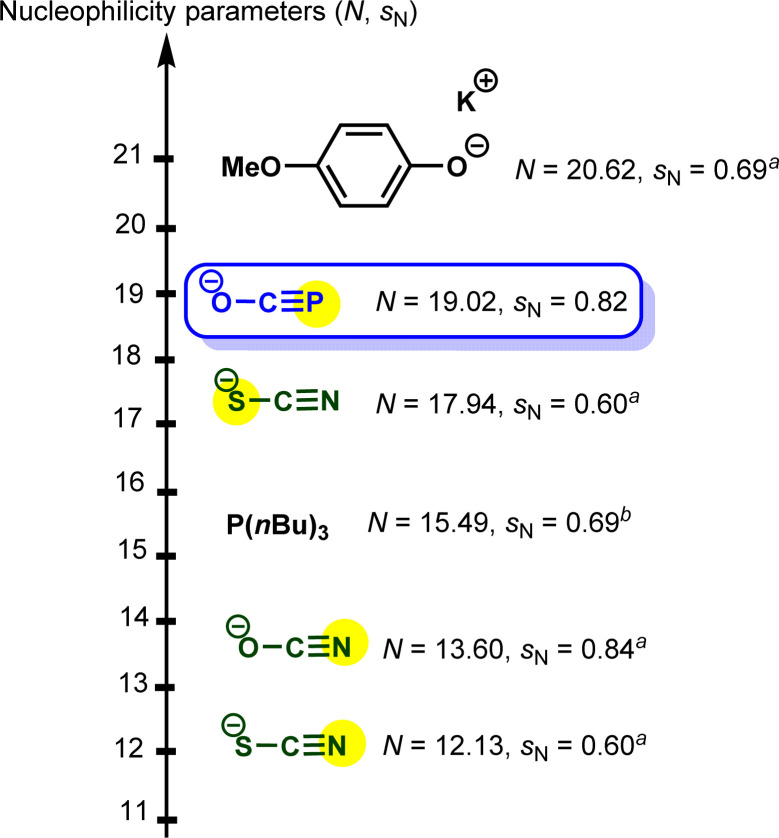
Embedding sodium phosphaethynolate in the Mayr nucleophilicity scale. ^*a*^ Solvent: acetonitrile. ^*b*^ Solvent: dichloromethane.^21–24^

Based on the results highlighted above, one can assign the measured nucleophilicity (*N* = 19.02, *s*_N_ = 0.82) to the phosphorus site of the phosphaethynolate, which is five orders of magnitude more reactive than cyanate anion^21^ and ten times more reactive than the N-terminus of thioisocyanate.^22^ This high reactivity may explain the capability of this anion to react with a wide variety of electrophiles, including weak ones such as carbodiimide (*E* ≈ −20) (Fig. 3).^23,24^

**Scheme 6:**
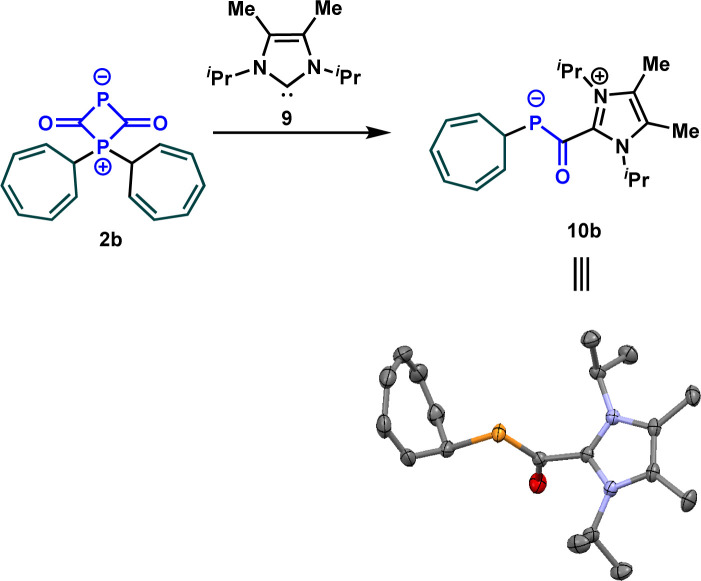
Reaction of the zwitterion 2b with the carbene 9.

After determining the phosphorus nucleophilicity of the OCP anion, we now wondered how this knowledge can be used in synthetically useful transformations. The investigation of the mechanism of the reaction of sodium phosphaethynolate with stabilized carbocations, for instance 1b, revealed the formation of the zwitterion 2b that was detected at −60 °C. To convert the latter into an isolable intermediate, we treated it with the NHC carbene 9. Notably, this resulted in the formation of azolium phosphaenolate 10b in quantitative yield, which can be described as the NHC adduct of 4b. The structure of 10b was confirmed by a single crystal X-ray diffraction experiment (Scheme 6). In the solid state, the C–P and C–O bond lengths are 1.737 and 1.264 Å, respectively. These results are in good agreement with those reported by Stephan, Cummins *et al.*^25^ for acylphosphide anions, where experimental and computational studies revealed that the short C–P and long C–O bond are the consequence of the delocalization of electron density from the phosphide lone pair into π*(C=O) orbital.

**Scheme 7:**
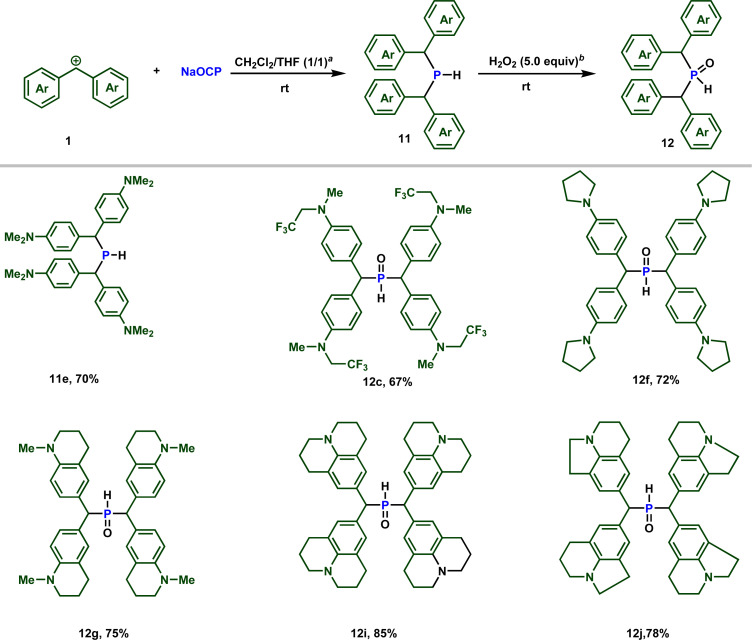
Reactivity of sodium phosphaethynolate towards stabilized carbocations. ^*a*^ 0.5 equiv. of water. ^*b*^ H_2_O_2_ 30% (w/w) in water was used.

Importantly, when the zwitterion 2e is dissolved in (dichloromethane/THF) in the presence of 0.5 equiv. of water, the secondary phosphine 11e is formed, as observed by ^31^P NMR spectroscopy (see ESI‡). Conducting the reaction in the presence of D_2_O resulted in the formation of deuterated phosphine, providing confirmation that the proton originates from water (Scheme 7).

Building upon these observations, we combined different stabilized carbocations 1 with Na(OCP) in THF/dichloromethane (1 : 1) mixture, containing 0.5 equiv. of water. Notably, employing stabilized carbocations (1c, f, g, i, j) led to the formation of secondary phosphines 11 and secondary phosphine oxides 12 in good to excellent yields, as confirmed by ^31^P NMR. While the phosphine 11e is stable to be isolated by column chromatography, the other phosphines oxidize rapidly when exposed to air. Consequently, these were treated with 5 equivalents of H_2_O_2_ and isolated as secondary phosphine oxides 12 in synthetically useful yields (Scheme 7).

**Scheme 8:**
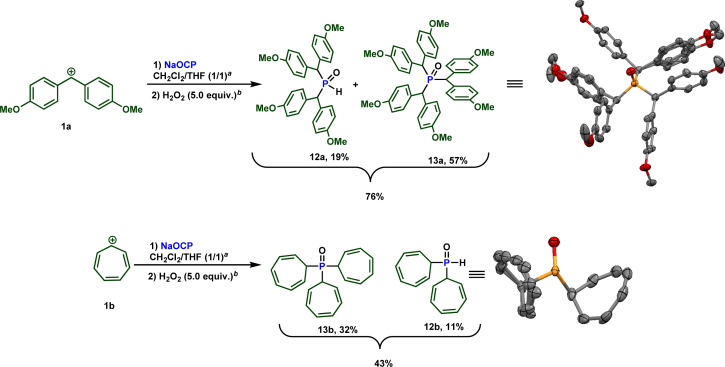
Reactivity of sodium phosphaethynolate towards highly reactive carbocations 1a and 1b. ^*a*^ 0.5 equiv. of water. ^*b*^ H_2_O_2_ 30% (w/w) in water was used.

Due to the high electrophilicity and Lewis acidity of 1a and 1b,^19*a*^ their reactions with the OCP anion produced a mixture of secondary and tertiary phosphines. Clearly, the formed secondary phosphines promptly react with 1a and 1b to give the tertiary phosphines. Both phosphines are oxidized to the phosphine oxides and isolated separately. The overall isolated yields are consistently good to very good (Scheme 8).

It is important to note that while an elegant synthesis of acyl phosphines and related molecules from the phosphaethynolate anion and related structures from [OCP]^−^ has been previously documented by Goicoechea,^11*b*^ Stephan,^26^ and others, direct synthesis of the sterically hindered phosphines 12 at 13 has not been described to our knowledge. Furthermore, traditional methods for obtaining these types of molecules would necessitate the use of strong Brønsted bases. Therefore, this approach offers a more convenient route as it enables access to these compounds under mild and sustainable conditions.

**Scheme 9:**
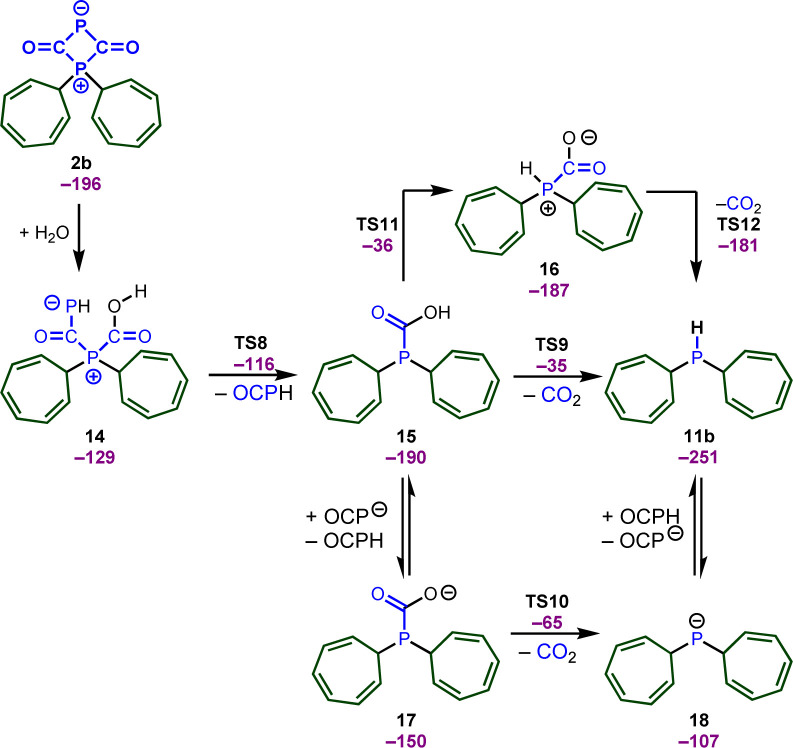
Potential pathways for the formation of secondary phosphines.

We again relied on DFT calculations to gain further insights into the mechanism of this useful transformation (Scheme 9). To simplify the computational studies, we continue to use the tropylium cation 1b as the initial electrophile. Based on our DFT calculations, the formation of the secondary phosphine 11b, CO_2_, and phosphaneylidenemethanone (OCPH) is thermodynamically feasible (Δ*G* = −40 kJ mol^−1^). Mechanistically, we propose that the zwitterion 2b initially reacts with water to yield intermediate 14. No barrier could be detected for the attack of water and the computational studies indicate that the attack of water and the proton transfer from water to the phosphorus atom occurs in a concerted fashion. This intermediate quickly looses OCPH *via*TS8 and affords the phosphanecarboxylic acid 15. A concerted decarboxylation is generally feasible and affords the secondary phosphine 11b in an overall favorable process, but the barrier for this step is very high (TS9, Δ*G*^‡^ = 155 kJ mol^−1^). Thus, a concerted process is very unlikely under the reaction conditions. Instead, the OCP anion could deprotonate the carboxylic acid and the decarboxylation takes place through an anionic transition state TS10 (Δ*G*^‡^ = 125 kJ mol^−1^). This is still a substantial barrier, but might be slightly overestimated due to the involved proton transfer. A more likely scenario is the formation of a zwitterionic phosphanecarboxylic acid 16. Although a formal 1,3 proton shift through TS11 is very unfavorable, another molecule 15 or the OCP anion will facilitate the proton transfer. The zwitterion 16 readily undergoes the decarboxylation (TS12) and affords the secondary phosphane in a very rapid reaction.

We should emphasize that consistent with the computational studies, the formation of OCPH was experimentally confirmed, as its dimer was detected when 2b was reacted with water (0.5 equiv.) at −60 °C (see ESI,‡ pages S93 and S94) (^31^P{^1^H} NMR spectrum at 305.8 and 75.9 ppm with a ^2^*J*_P–P_ coupling constant of 16.0 Hz, as well as a doublet of doublets at 7.32 ppm (^1^*J*_P–H_ = 172, ^3^*J*_P–H_ = 16.0 Hz)). This finding aligns well with Goicoechea's observation that OCPH undergoes dimerization.^27^

**Scheme 10:**
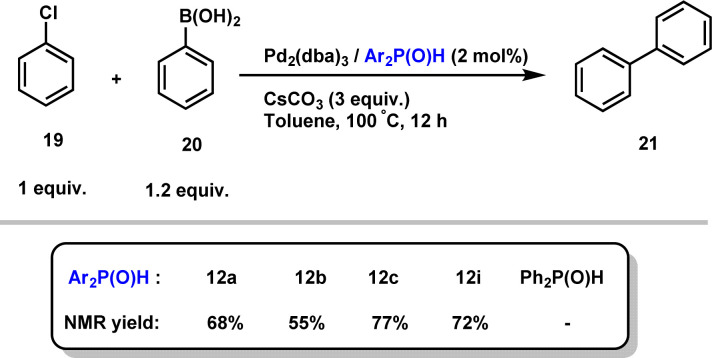
Secondary phosphine oxide/Pd_2_(dba)_3_ catalysed the Suzuki cross-coupling reaction of 19 with 20.

Finally, given that hindered secondary phosphine oxides have proven to be effective ligands for transition metal-catalyzed reactions, as initially demonstrated by Li,^28^ we evaluated their combination with Pd_2_(dba)_3_ in the Suzuki cross-coupling reaction of aryl chloride 19 with phenylboronic acid 20.^29^ Importantly, the desired biphenyl product 21 in good yields, showing the efficiency of our secondary phosphine oxides (Scheme 10). Interestingly, no reaction occurred when diphenylphosphine oxide was used as a ligand, highlighting the crucial role of sterically hindered phosphines.

## Conclusions

In conclusion, we herein provide an experimental quantification of the phosphorus nucleophilicity of the sodium phosphaethynolate. This reactivity is both kinetically and thermodynamically favourable. Mechanistic investigations allowed the identification and full characterization of key intermediates such as the zwitterion 2, and also the optimization of experimental conditions enabling the synthesis of synthetically useful organophosphorus molecules such as sterically hindered secondary phosphines oxides. The latter have shown to be effective ligands in Suzuki cross coupling reaction.

## Data availability

All experimental procedures, details of the calculations, and additional data can be found in the ESI.‡

## Author contributions

T. H. V. N. conducted synthetic and kinetic experiments, while S. C. analysed the kinetic data and carried out experiments using laser-flash photolysis equipment. S. M.-L. conducted the X-ray experiments. M. B. performed all calculations. S. L. conceived and supervised the project. All authors participated in discussing the results, providing comments, and proofreading the manuscript.

## Conflicts of interest

The authors declare no competing interests.

## Supplementary Material

SC-OLF-D4SC03518F-s001

SC-OLF-D4SC03518F-s002

SC-OLF-D4SC03518F-s003

SC-OLF-D4SC03518F-s004

SC-OLF-D4SC03518F-s005

SC-OLF-D4SC03518F-s006

SC-OLF-D4SC03518F-s007

SC-OLF-D4SC03518F-s008

SC-OLF-D4SC03518F-s009

SC-OLF-D4SC03518F-s010

SC-OLF-D4SC03518F-s011

SC-OLF-D4SC03518F-s012

SC-OLF-D4SC03518F-s013

SC-OLF-D4SC03518F-s014

SC-OLF-D4SC03518F-s015

SC-OLF-D4SC03518F-s016

SC-OLF-D4SC03518F-s017

SC-OLF-D4SC03518F-s018

SC-OLF-D4SC03518F-s019

SC-OLF-D4SC03518F-s020

SC-OLF-D4SC03518F-s021

SC-OLF-D4SC03518F-s022

SC-OLF-D4SC03518F-s023

SC-OLF-D4SC03518F-s024

SC-OLF-D4SC03518F-s025

SC-OLF-D4SC03518F-s026

SC-OLF-D4SC03518F-s027

SC-OLF-D4SC03518F-s028

SC-OLF-D4SC03518F-s029

SC-OLF-D4SC03518F-s030

SC-OLF-D4SC03518F-s031

SC-OLF-D4SC03518F-s032

SC-OLF-D4SC03518F-s033

SC-OLF-D4SC03518F-s034

SC-OLF-D4SC03518F-s035

SC-OLF-D4SC03518F-s036

SC-OLF-D4SC03518F-s037

SC-OLF-D4SC03518F-s038

SC-OLF-D4SC03518F-s039

SC-OLF-D4SC03518F-s040

SC-OLF-D4SC03518F-s041

SC-OLF-D4SC03518F-s042

SC-OLF-D4SC03518F-s043

SC-OLF-D4SC03518F-s044

SC-OLF-D4SC03518F-s045

SC-OLF-D4SC03518F-s046

SC-OLF-D4SC03518F-s047

SC-OLF-D4SC03518F-s048

SC-OLF-D4SC03518F-s049

SC-OLF-D4SC03518F-s050

SC-OLF-D4SC03518F-s051

SC-OLF-D4SC03518F-s052

SC-OLF-D4SC03518F-s053

SC-OLF-D4SC03518F-s054

SC-OLF-D4SC03518F-s055

SC-OLF-D4SC03518F-s056

SC-OLF-D4SC03518F-s057

SC-OLF-D4SC03518F-s058

SC-OLF-D4SC03518F-s059

SC-OLF-D4SC03518F-s060

SC-OLF-D4SC03518F-s061

SC-OLF-D4SC03518F-s062

SC-OLF-D4SC03518F-s063

SC-OLF-D4SC03518F-s064

SC-OLF-D4SC03518F-s065

SC-OLF-D4SC03518F-s066

SC-OLF-D4SC03518F-s067

SC-OLF-D4SC03518F-s068

SC-OLF-D4SC03518F-s069

SC-OLF-D4SC03518F-s070

SC-OLF-D4SC03518F-s071

SC-OLF-D4SC03518F-s072

SC-OLF-D4SC03518F-s073

SC-OLF-D4SC03518F-s074

## References

[cit1] Corbridge D. E. C. , Phosphorus 2000. Chemistry, Biochemistry & Technology, Elsevier, Amsterdam, 2002

[cit2] Schipper W. (2014). Eur. J. Inorg. Chem..

[cit3] Büchel K. H. , Moretto H. H. and Woditsch P., Industrial Inorganic Chemistry, Wiley VCH, New York, 2nd edn, 2000, pp. 65–101

[cit4] Montchamp J. L. (2014). Acc. Chem. Res..

[cit5] Scott D. J. (2022). Angew. Chem., Int. Ed..

[cit6] Goicoechea J. M., Grützmacher H. (2018). Angew. Chem., Int. Ed..

[cit7] (b) Dillon K. B. , Mathey F. and Nixon J. F., Phosphorus: The Carbon Copy, Wiley, Chichester, 1998

[cit8] Alidori S., Heift D., Santiso-Quinones G., Benkő Z., Grützmacher H., Caporali M., Gonsalvi L., Rossin A., Peruzzini M. (2012). Chem.–Eur. J..

[cit9] Becker G., Schwarz W., Seidler N., Westerhausen M. (1992). Z. Anorg. Allg. Chem..

[cit10] Puschmann F. F., Stein D., Heift D., Hendriksen C., Gal Z. A., Grützmacher H.
F., Grützmacher H. (2011). Angew. Chem., Int. Ed..

[cit11] Tondreau A. M., Benkő Z., Harmer J. R., Grützmacher H. (2014). Chem. Sci..

[cit12] Heift D., Benkő Z., Grützmacher H. (2014). Dalton Trans..

[cit13] Horváth Á., Lőrincz B. D., Benkő Z. (2023). Chem. Eur. J..

[cit14] Mayr H., Breugst M., Ofial A. R. (2011). Angew. Chem., Int. Ed..

[cit15] Pearson R. G. (1966). Science.

[cit16] Mayr H., Ofial A. R. (2015). SAR QSAR Environ. Res..

[cit17] Mayr H., Müller K.-H., Ofial A. R., Bühl M. (1999). J. Am. Chem. Soc..

[cit18] (d) The database of Mayr reactivity parameters (*N*, *s*_N_, and *E*), https://www.cup.lmu.de/oc/mayr/reaktionsdatenbank2/, accessed 27/05/2024

[cit19] Mayr H., Ammer J., Baidya M., Maji B., Nigst T. A., Ofial A. R., Singer T. (2015). J. Am. Chem. Soc..

[cit20] Szkop K. M., Jupp A. R., Suter R., Grützmacher H., Stephan D. W. (2017). Angew. Chem., Int. Ed..

[cit21] Tishkov A. A., Mayr H. (2005). Angew. Chem., Int. Ed..

[cit22] Loos R., Kobayashi S., Mayr H. (2003). J. Am. Chem. Soc..

[cit23] Liu L., Zhu J., Zhao Y. (2014). Chem. Commun..

[cit24] Li Z., Mayer R. J., Ofial A. R., Mayr H. (2020). J. Am. Chem. Soc..

[cit25] Szkop K. M., Geeson M. B., Stephan D. W., Cummins C. C. (2019). Chem. Sci..

[cit26] Szkop K. M., Jupp A. R., Razumkov H., Stephan D. W. (2020). Dalton Trans..

[cit27] Hinz A., Labbow R., Rennick C., Schulz A., Goicoechea J. M. (2017). Angew. Chem., Int. Ed..

[cit28] Li G. Y. (2002). J. Org. Chem..

[cit29] Shaikh T. M., Weng Ch-M., Hong F.-E. (2012). Coord. Chem. Rev..

