# Supplementary material for: Reactivity of the phosphaethynolate anion with stabilized carbocations: mechanistic studies and synthetic applications
Source: Chem Sci. 2024 Aug 8;15(35):14406–14. doi: 10.1039/d4sc03518f (PMC11331332; doi:10.1039/d4sc03518f)
Supplement: SC-015-D4SC03518F-s040 [file SC-015-D4SC03518F-s040.pdf]

Supplementary Information (SI) for Chemical Science.
This journal is © The Royal Society of Chemistry 2024

Supporting Information

Reactivity of Phosphaethynolate Anion with Stabilized Carbocations: Mechanistic Studies and Synthetic Applications

Thi Hong Van Nguyen,[a] Saloua Chelli,[a] Sonia Mallet-Ladeira,[b] Martin Breugst,*[c] and Sami Lakhdar*[a]

[a] T. H. V. Nguyen, Dr. S. Chelli, Dr. S. Lakhdar
CNRS/Université Paul Sabatier, Laboratoire Hetérochimie Fondamentale et Appliquée (LHFA, UMR5069)
118 Route de Narbonne,31062Cedex 09 Toulouse, France.
Institution
E-mail: sami.lakhdar@univ-tlse3.fr

[b] S. Mallet-Laderia
Institut de Chimie de Toulouse(FR 2599)
118 Route de Narbonne, 31062Cedex 09 Toulouse, France.

[c] Prof. Dr. M. Breugst
Institut für Chemie
Technische Universität Chemnitz, 09111 Chemnitz, Germany
E-mail: martin.breugst@chemie.tu-chemnitz.de

Table of Contents

1. General Information 1
2. Synthesis of sodium phosphaethynolate [Na(OCP)](dioxane)$_x$ 1
3. Product synthesis 2
3.1. General Procedure A 2
3.2. General Procedure B 3
3.3. General Procedure C 3
4. Preparation of phosphines 11-12 3
5. Preparation of intermediates 2e and 3e 10
6. Preparation of 10b 11
7. Procedure for catalytic application of phosphines in Suzuki Cross-coupling reaction 12
8. Procedure for detection of HPCO 12
9. Kinetic experiments 13
9.1. Conventional UV-Vis Spectroscopy 13
9.2. Laser flash photolysis 13
9.3. Determination of the Rate Constants for the reactions of phosphaethynolate anion with carbocations 14
10. Computational details 25
11. Crystallographic data 26
12. References 28
13. Copies of NMR Spectra and HRMS 29

1. General Information

All reactions were conducted in flame-dried glassware under argon atmosphere or inside of the glovebox. Reactions were set up using standard Schlenk technique with a glass vacuum manifold connected to an inlet of dry argon gas. Tetrahydrofuran (THF), methylene chloride (DCM), acetonitrile (CH_3CN) were purified using a MBraun SPS solvent purification system by purging with nitrogen then stocked in a rotaflow with the activated molecular sieves and degassed in prior to use. 1,2-Dimethoxyethane (DME) and dioxane were purchased from TCI Chemical and distilled over sodium/benzophenone, degassed before using. Other solvents were purchased as anhydrous and used as received.

[Na(OCP)](dioxane)$_x$ was prepared by using published method.[1] All the electrophiles were synthesized according to described method in references.[2–4] The phosphonium were prepared following procedure described before.[5] All others reagents were purchased from Sigma Aldrich, TCI Chemical or Thermo Fisher Scientific and used without further purification.

^{1}H NMR spectra were recorded on Bruker 600 Hz, 500 MHz or 300 MHz spectrometer. Chemical shifts are reported in parts per million (ppm) and the spectra are calibrated to the resonance resulting from incomplete deuteration of the solvent (Chloroform-*d*: 7.26 ppm; Methylene Chloride-d_2: 5.32 ppm; Tetrahydrofuran-d_8: 3.58 ppm and 1.73 ppm). ^{13}C DEPT or Jmod NMR spectra were recorded on the same spectrometers with complete proton decoupling. Chemical shifts are reported in ppm with the solvent resonance as the internal standard (Chloroform-*d*: 77.12 ppm; Methylene Chloride-d_2: 53.84 ppm; Tetrahydrofuran-d_8: 67.57 ppm and 25.37 ppm. Data are reported using the abbreviations: s = singlet, d = doublet, t = triplet, q = quartet, p = pentet, h = hextet, m = multiplet, br = broad. Coupling constant(s) are reported in Hz. ^{31}P NMR spectra and ^{19}F NMR spectra were recorded on the same spectrometers as above. ^{1}H-COSY, HSQC and HMBC were used where appropriate to facilitate structural determination.

High resolution mass spectrometry data (HRMS) were obtained on an Agilent 6230 TOF LC/MS equipped with ESI detector in positive mode and on Micromass AutoSpec Ultima Magnetic Sector instrument with EI detector in positive mode.

2. Synthesis of sodium phosphaethynolate [Na(OCP)](dioxane)$_x$

To a 500 mL schlenk flask, red phosphorus (3.5 g, 0.110 mol) and naphthalene (0.75 g, 0.006 mol) was added inside the glovebox. The schlenk flask was then removed from glovebox and flushed three times with argon and subsequently added DME (160 mL) *via* syringe. To this suspension, sodium cube (8.0 g, 0.35 mol) was cut as small as possible under argon flux. The

mixture was stirred at room temperature for at least 12 hours. The resulting black suspension was cooled with an ice bath and *tert*-butanol (21 mL, 0.22 mol) was added dropwise *via* a syringe. Subsequently the reaction mixture was allowed to warmed up to room temperature and stirred for another 1 hour. After that, a solution of ethylene carbonate (10.0 g, 0.12 mol) in DME (50 mL) was added slowly at 0°C. Once the addition is completed, the suspension was stirred at room temperature overnight. The next day, the solvent was removed under vacuum, the remaining solid was dissolved in THF (500 mL) and filtered over Celite® under argon atmosphere. The resulting light-yellow filtrate was concentrated to 20 mL approximately. To this concentrated solution, dioxane (20 mL) was added, resulting the precipitation of colorless product as sodium phosphaethynolate [Na(OCP)](dioxane)$_{x.}$ (where x = 1 – 2.5, determined by ^{1}H NMR using naphthalene as standard) which was collected by filtration and washed with dioxane. The raw product was recrystallized in THF-Dioxane to give pure product in good agreement with literature.[1]

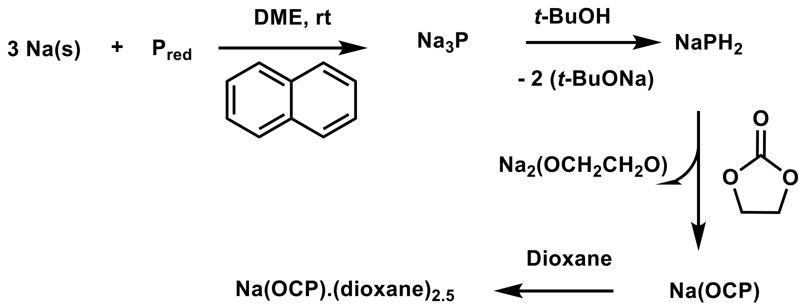

Scheme 1. Synthesis of sodium phosphaethynolate [Na(OCP)](dioxane)$_x$

3. Product synthesis

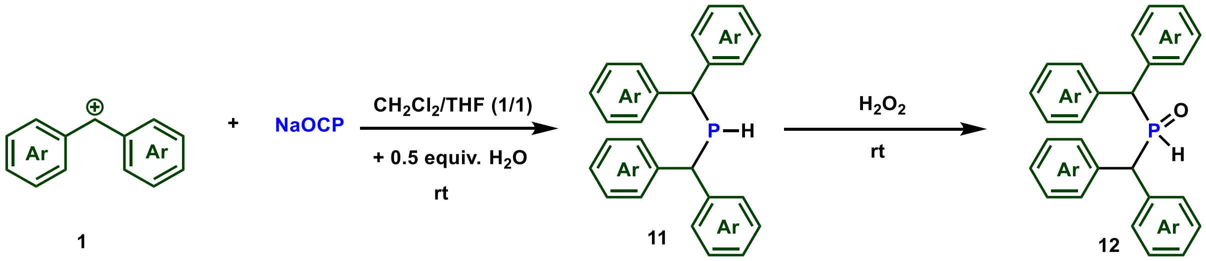

Scheme 2. Product synthesis

3.1.General Procedure A

To a stirred solution of benzhydrylium cation (1.0 equiv., 10^{-2} M) in CH_2Cl_2 under argon at room temperature, was added dropwise a solution of [Na(OCP)](dioxane)$_x$ (1.2 equiv., 10^{-2} M) in THF contains 0.5 equiv. of water. The reaction mixture was allowed to stir for 2 hours then the solvent was removed under reduced pressure. The obtained residue was partially dissolved in CH_2Cl_2 and filtered off to give a yellow solution as the crude product which was

then purified by column chromatography using pentane/ ethyl acetate (7/3) as eluent to give desired product.

3.2. General Procedure B

To a stirred solution of benzhydrol (1.0 equiv., 10^{-2} M) in CH_2Cl_2 under argon at -60 ^oC, was added dropwise a solution of $HBF_4.Et_2O$ (1.2 equiv.). After 5 minutes, the solution of [Na(OCP)](dioxane)$_x$ (1.2 equiv., 10^{-2} M) in THF (contains 0.5 equiv. of water) was added dropwise at -60 ^oC. The reaction mixture was allowed to stir for 3 hours before adding dropwise solution hydrogen peroxide 35% in water (5.0 equiv.). After stirring for another 1 hour at rt, the reaction mixture was washed with water then with brine solution. The aqueous phase was extracted with CH_2Cl_2 (3x). Then the combined organic layers were dried with Na_2SO_4 and the solvent was evaporated under reduced pressure. The crude products were purified by column chromatography using petroleum ether/ ethyl acetate (5/5) as eluent to give desired product.

3.3. General Procedure C

To a solution of benzhydrylium cation (1.0 equiv., 10^{-2} M) in CH_2Cl_2, a solution of [Na(OCP)](dioxane)$_x$] (1.2 equiv., 10^{-2} M) in THF (contains 0.5 equiv. of water) was added dropwise under argon at room temperature. The reaction mixture was stirred at room temperature for 3 hours before adding dropwise solution hydrogen peroxide 35% in water (5.0 equiv.). After stirring for another 1 hour, the reaction mixture was washed with water and then with brine solution. The aqueous phase was extracted with CH_2Cl_2 (3x). Then the combined organic layers were dried with Na_2SO_4 and the solvent was evaporated under reduced pressure. The crude products were purified by column chromatography using petroleum ether/ ethyl acetate (5/5) as eluent to give desired product.

4. Preparation of phosphines 11-12

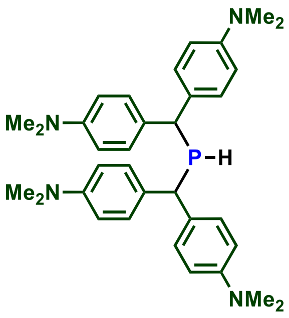

11e, 70%

Following general procedure **A** using tetrafluoroborate of **1e** (0.20 mmol, 68.0 mg, 1.0 equiv.), [Na(OCP)](dioxane)$_{2.5}$ (0.24 mmol, 72.5 mg, 1.2 equiv.), and H_2O (0.10 mmol, 1.8

mg) in 10 mL THF/CH_2Cl_2 (1/1), purifying via silica column chromatography using pentane / ethyl acetate (7/3) as eluent gave product **11e** in 70% yield as a white solid.

^{31}P NMR (121 MHz, Chloroform-*d*) δ -22.3 (d, *J* = 201.5 Hz).

^{31}P{H} NMR (121 MHz, Chloroform-*d*) δ -22.5 (s).

^{1}H NMR (300 MHz, Chloroform-*d*) δ 7.16 (d, *J* = 6 Hz, 4H), 7.08 (d, *J* = 6 Hz, 4H), 6.67 (d, *J* = 3.2 Hz, 4H), 6.64 (d, *J* = 3.2 Hz, 4H), 4.20 and 3.53 (td, *J* = 201.5, 7.6 Hz, 1H), 3.92 (dd, *J* = 7.7, 2.2 Hz, 2H), 2.90 (s, 12H), 2.90 (s, 12H).

JMOD ^{13}C NMR (75 MHz, Chloroform-*d*) δ 149.2, 149.0, 132.2 (d, *J* = 2.5 Hz), 130.9 (d, *J* = 12.4 Hz), 129.4 (d, *J* = 9.9 Hz,), 129.0 (d, *J* = 3.6 Hz), 113.0, 43.8, 43.6, 41.0, 40.9.

HRMS (ESI) m/z calcd for $C_{34}H_{44}N_4P$ $(M+H)^+$: 539.3304, found : 539.3286

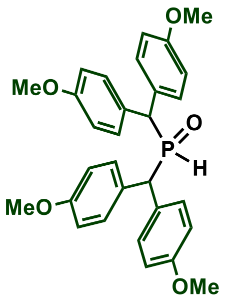

12a, 19%

Following general procedure **B** using 4,4-dimethoxybenzhydrol (0.20 mmol, 50 mg, 1.0 equiv.), HBF_4 (0.24 mmol, 38.8 mg, 1.2 equiv.), [Na(OCP)](dioxane)$_{2.5}$ (0.24 mmol, 72.5 mg, 1.2 equiv.) and H_2O (0.10 mmol, 1.8 mg) in 10 mL THF/CH_2Cl_2 (1/1) in 10 mL THF/CH_2Cl_2 (1/1), purifying via silica column chromatography using pentane / ethyl acetate (7/3 to 5/5) as eluent gave product **12a** by 19% yield as a white solid.

^{31}P NMR (162 MHz, Methylene Chloride-*d*$_2$) δ 41.1 (dt, *J* = 460.8, 14.4 Hz).

^{31}P{H} NMR (162 MHz, Methylene Chloride-*d*$_2$) δ 41.1 (s).

^{1}H NMR (500 MHz, Methylene Chloride-*d*$_2$) δ 7.24 (dt, *J* = 460.8 Hz, 3.3 Hz, 1H), 7.30 (d, *J* = 8.7 Hz, 4H), 7.16 (dd, *J* = 8.7, 1.5 Hz, 4H), 6.91 – 6.82 (m, 8H), 4.00 (d, *J* = 3.4 Hz, 1H), 3.98 (d, *J* = 3.4 Hz, 1H), 3.79 (s, 12H).

JMOD ^{13}C NMR (126 MHz, Methylene Chloride-*d*$_2$) δ 159.5 (d, *J* = 2.2 Hz), 159.4 (d, *J* = 1.5 Hz), 131.0 (d, *J* = 6.9 Hz), 130.4 (d, *J* = 6.7 Hz), 129.5 (d, *J* = 2.8 Hz), 128.5 (d, *J* = 4.6 Hz), 114.8 (d, *J* = 1.6 Hz), 114.5, 55.7 (d, *J* = 4.7 Hz), 49.3, 48.9.

HRMS (DCI) m/z calcd for $C_{30}H_{31}O_5P$ $(M)^+$: 502.1909, found : 502.1922

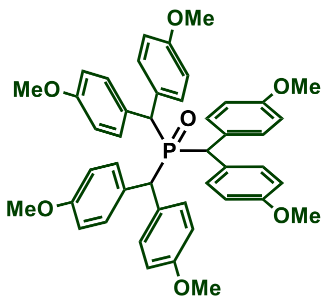

13a, 57%

Following general procedure **B** using 4,4-dimethoxybenzhydrol (0.20 mmol, 50.0 mg, 1.0 equiv.), HBF_4 (0.24 mmol, 38.8 mg, 1.2 eq), [Na(OCP)](dioxane)$_{2.5}$ (0.24 mmol, 72.5 mg, 1.2 equiv.) and H_2O (0.10 mmol, 1.8 mg) in 10 mL THF/CH_2Cl_2 (1/1), purifying via silica column chromatography using pentane / ethyl acetate (7/3 to 5/5) as eluent gave product **13a** by 57% yield as a white solid.

^{31}P NMR (162 MHz, Methylene Chloride-d_2) δ 46.2 (q, J = 12.0 Hz).

^{31}P{H} NMR (162 MHz, Methylene Chloride-d_2) δ 46.2 (s).

^{1}H NMR (500 MHz, Methylene Chloride-d_2) δ 7.09 (d, J = 10.0 Hz, 12H), 6.57 (d, J =10.0 Hz, 12H), 4.23 (d, J = 10.0 Hz, 3H), 3.72 (s, 18H).

JMOD ^{13}C NMR (126 MHz, Methylene Chloride-d_2) δ 158.5, 131.1 (d, J = 3.3 Hz), 130.7 (d, J = 5.7 Hz), 113.9, 55.4, 52.5 (d, J = 55.7 Hz).

HRMS (ESI) m/z calcd for $C_{45}H_{46}O_7P$ $(M+H)^+$: 729.2981, found : 729.3049

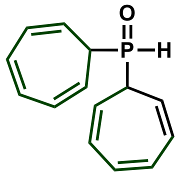

12b, 11%

Following general procedure **C** using tropylium tetrafluoroborate **1b** (0.20 mmol, 35.5 mg, 1.0 equiv.), [Na(OCP)](dioxane)$_{2.5}$ (0.24 mmol, 72.5 mg, 1.2 equiv.), H_2O (0.10 mmol, 1.8 mg) in 10 mL THF/CH_2Cl_2 (1/1) and H_2O_2 (1.0 mmol, 34.0 mg, 5.0 equiv.), purifying via silica column chromatography using petroleum ether / ethyl acetate (6/4) as eluent gave product **12b** by 11% yield as a white solid.

^{31}P NMR (121 MHz) δ 23.2 (dq, J = 482.9, 11.4 Hz).

^{31}P{H} NMR (121 MHz, Chloroform-d) δ 23.2 (s).

^{1}H NMR (300 MHz, Chloroform-*d*) δ 6.99 (dt, *J* = 482.9, 4.2 Hz, 1H), 6.68 – 6.48 (m, 4H), 6.43 (dd, *J* = 9.7, 5.7 Hz, 2H), 6.32 (dd, *J* = 9.5, 5.7 Hz, 2H), 5.48 (dt, *J* = 9.7, 7.5 Hz, 2H), 5.37 – 5.19 (dt, *J* = 9.7, 7.5 Hz, 2H), 2.76 (dtd, *J* = 11.9, 7.5, 4.2 Hz, 2H).

JMOD ^{13}C NMR (75 MHz, Chloroform-*d*) δ 131.5 (d, *J* = 54.3 Hz), 129.3 (d, *J* = 3.4 Hz), 129.2 (d, *J* = 2.4 Hz), 116.4 (d, *J* = 1.9 Hz), 116.1 (d, *J* = 5.1 Hz), 38.7 (d, *J* = 69.2 Hz).

HRMS (DCI) m/z calcd for $C_{14}H_{16}OP$ $(M+H)^+$: 231.0939, found : 231.0944

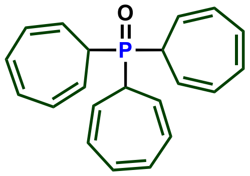

13b, 32%

Following general procedure **C** using tropylium tetrafluoroborate **1b** (0.20 mmol, 35.5 mg, 1.0 equiv.), [Na(OCP)](dioxane)$_{2.5}$ (0.24 mmol, 72.5 mg, 1.2 equiv.), H_2O (0.10 mmol, 1.8 mg) in 10 mL THF/CH_2Cl_2 (1/1) and H_2O_2 (1.0 mmol, 34.0 mg, 5.0 equiv.) purifying via silica column chromatography using petroleum ether / ethyl acetate (6/4) as eluent gave product **13b** by 32% yield as a white solid.

^{31}P NMR (202 MHz, Methylene Chloride-*d*$_2$) δ 45.5 (m).

^{31}P{H} NMR (202 MHz, Methylene Chloride-*d*$_2$) δ 45.4 (s).

^{1}H NMR (500 MHz, Methylene Chloride-*d*$_2$) δ 6.58 (dd, *J* = 4.0, 2.7 Hz, 6H), 6.35 – 6.32 (m, 6H), 5.14 (ddd, *J* = 10.5, 8.5, 6.5 Hz, 6H), 2.03 (q, *J* = 6.6 Hz, 3H).

JMOD ^{13}C NMR (126 MHz, Methylene Chloride-*d*$_2$) δ 130.5, 128.1 (d, *J* = 12.0 Hz), 106.2 (d, *J* = 1.9 Hz), 36.2 (d, *J* = 74.1 Hz).

HRMS (DCI) m/z calcd for $C_{21}H_{22}OP$ $(M+H)^+$: 321.1408, found : 321.1394

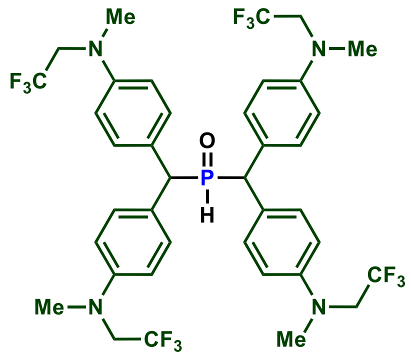

12c, 67%

Following general procedure **C** using tetrafluoroborate of **1c** (0.20 mmol, 95.2 mg, 1.0 equiv.), [Na(OCP)](dioxane)$_{2.5}$ (0.24 mmol, 72.5 mg, 1.2 equiv.), H_2O (0.10 mmol, 1.8 mg) in 10 mL THF/CH_2Cl_2 (1/1) and H_2O_2 (1.0 mmol, 34.0 mg, 5.0 equiv.), purifying via silica column chromatography using petroleum ether / ethyl acetate (6/4) as eluent gave product **12c** by 67 % yield as a white solid.

^{31}P NMR (202 MHz, Methylene Chloride-d_2) δ 41.2 (dt, J = 457.1, 14.1 Hz).

^{31}P{H} NMR (202 MHz, Methylene Chloride-d_2) δ 41.2 (s).

^{19}F NMR (471 MHz, Methylene Chloride-d_2) δ -70.9 (dt; J = 19.1, 8.9 Hz).

^{1}H NMR (500 MHz, Methylene Chloride-d_2) δ 7.21 (dt, J = 457.1, 3.3 Hz, 1H), 7.26 (dd, J = 8.9, 1.4 Hz, 4H), 7.13 (dd, J = 8.9, 1.4 Hz, 4H), 6.78 – 6.74 (m, 8H), 3.95 (d, J = 3.4 Hz, 1H), 3.92 (d, J = 3.2 Hz, 1H), 3.88 (qd, J = 9.1, 1.9 Hz, 8H), 3.03 (s, 12H).

JMOD ^{13}C NMR (126 MHz, Methylene Chloride-d_2) δ 148.4 (d, J = 2.0 Hz), 148.2, 130.7 (d, J = 6.8 Hz), 130.1 (d, J = 6.5 Hz), 129.5 (d, J = 4.6 Hz), 127.3 (dd, J = 5.7, 3.7 Hz), 126.2 (d, J = 4.6 Hz), 125.0 (d, J = 4.8 Hz), 122.8 (d, J = 4.7 Hz), 113.4, 113.2, 55.1 – 54.0 (m), 49.1, 48.6, 39.5 (d, J = 2.7 Hz), 30.1.

HRMS (DCI) m/z calcd for $C_{38}H_{40}F_{12}N_4OP$ $(M+H)^+$: 827.2748, found : 827.2770

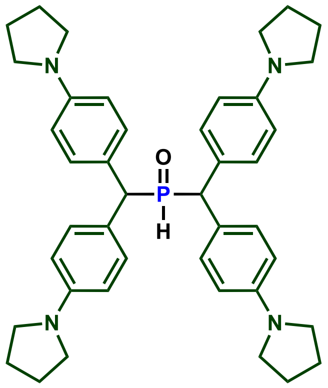

12f, 72%

Following general procedure **C** using tetrafluoroborate of **1f** (0.20 mmol, 78.5 mg, 1.0 equiv.), [Na(OCP)](dioxane)$_{2.5}$ (0.24 mmol, 72.5 mg, 1.2 equiv.), H_2O (0.10 mmol, 1.8 mg) in 10 mL THF/CH_2Cl_2 (1/1) and H_2O_2 (1.0 mmol, 34.0 mg, 5.0 equiv.), purifying via silica column chromatography using petroleum ether / ethyl acetate (3/7) as eluent gave product **12f** by 72 % yield as a white solid.

^{31}P NMR (202 MHz, Methylene Chloride-d_2) δ 43.0 (dt, J = 452.6, 14.6 Hz).

^{31}P{H} NMR (202 MHz, Methylene Chloride-d_2) δ 43.0 (s).

^{1}H NMR (500 MHz, Methylene Chloride-d_2) δ 7.15 (dt, J = 452.6, 3.6 Hz, 1H), 7.21 (d, J = 8.3 Hz, 4H), 7.05 (d, J = 8.3 Hz, 4H), 6.53 – 6.50 (m, 8H), 3.90 (d, J = 3.6 Hz, 1H), 3.87 (d, J = 3.6 Hz, 1H), 3.27 – 3.24 (m, 16H), 2.11 – 1.85 (m, 16H).

JMOD ^{13}C NMR (126 MHz, Methylene Chloride-d_2) δ 147.7 (d, J = 1.38 Hz), 147.6, 130.6 (d, J = 6.8 Hz), 130.0 (d, J = 6.4 Hz), 124.7 (d, J = 2.3 Hz), 123.7 (d, J = 4.3 Hz), 112.3, 112.1, 48.9, 48.8, 48.1, 25.8.

HRMS (DCI) m/z calcd for $C_{42}H_{51}N_4OP$ $(M)^+$: 658.3808, found : 658.3801

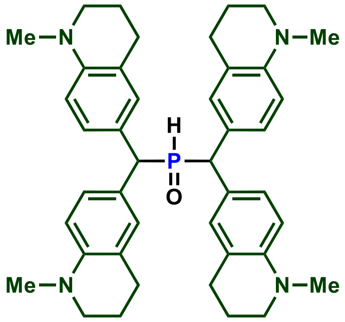

12g, 55%

Following general procedure **C** using tetrafluoroborate of **1g** (0.20 mmol, 78.5 mg, 1.0 equiv.), [Na(OCP)](dioxane)$_{2.5}$ (0.24 mmol, 72.5 mg, 1.2 equiv.), H_2O (0.10 mmol, 1.8 mg) in 10 mL THF/CH_2Cl_2 (1/1) and H_2O_2 (1.0 mmol, 34.0 mg, 5.0 equiv.), purifying via silica column chromatography using petroleum ether / ethyl acetate (3/7) as eluent gave product **12g** by 55 % yield as a white solid.

^{31}P NMR (202 MHz, Methylene Chloride-d_2) δ 42.1 (dt, J = 451.9, 14.5 Hz).

^{31}P{H} NMR (202 MHz, Methylene Chloride-d_2) δ 42.1 (s).

^{1}H NMR (500 MHz, Methylene Chloride-d_2) δ 7.14 (dt, J = 451.9, 3.5 Hz, 1H), 7.07 (dt, J = 8.7, 1.7 Hz, 2H), 6.92 (dt, J = 8.4, 1.9 Hz, 2H), 6.87 (d, J = 1.3 Hz, 2H), 6.74 (d, J = 1.2 Hz, 2H), 6.52 (t, J = 8.0 Hz, 4H), 3.79 (d, J = 3.5 Hz, 1H), 3.76 (d, J = 3.5 Hz, 1H), 3.28 – 3.10 (m, 8H), 2.86 (d, J = 4.0 Hz, 12H), 2.71 (q, J = 7.0 Hz, 8H), 1.95 (m, 8H).

JMOD ^{13}C NMR (126 MHz, Methylene Chloride-d_2) δ 146.4 (d, J = 1.7 Hz), 146.3, 130.3 (d, J = 7.2 Hz), 129.7 (d, J = 6.7 Hz), 128.3 (d, J = 6.8 Hz), 127.6 (d, J = 6.4 Hz), 125.7 (d, J = 2.5 Hz), 124.6 (d, J = 4.2 Hz), 123.6, 123.4, 111.4, 111.3, 51.6 (d, J = 5.9 Hz), 49.2, 48.7, 39.3 (d, J = 4.0 Hz), 28.2, 22.9 (d, J = 7.1 Hz).

HRMS (DCI) m/z calcd for $C_{42}H_{51}N_4OP$ $(M)^+$: 658.3801, found : 658.3774

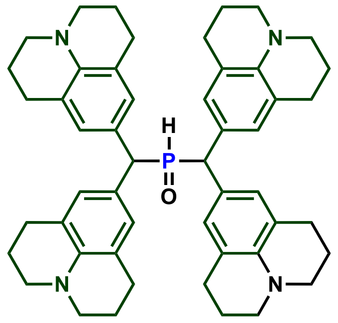

12i, **85%**

Following general procedure **C** using tetrafluoroborate of **1i** (0.20 mmol, 89.0 mg, 1.0 equiv.), $[Na(OCP)](dioxane)_{2.5}$ (0.24 mmol, 72.5 mg, 1.2 equiv.), H_2O (0.10 mmol, 1.8 mg) in 10 mL THF/CH_2Cl_2 (1/1) and H_2O_2 (1.0 mmol, 34.0 mg, 5.0 equiv.), purifying via silica column chromatography using petroleum ether / ethyl acetate (3/7) as eluent gave product **12i** by 85 % yield as a white solid.

^{31}P NMR (243 MHz, Methylene Chloride-d_2) δ 42.0 (dt, J = 450.8, 14.6 Hz).
^{31}P{H} NMR (243 MHz, Methylene Chloride-d_2) δ 42.0 (s).
^{1}H NMR (600 MHz, Methylene Chloride-d_2) δ 7.09 (dt, J = 450.8, 3.5 Hz, 1H), 6.71 (s, 4H), 6.59 (s, 4H), 3.66 (d, J = 3.5 Hz, 1H), 3.64 (d, J = 3.5 Hz, 1H), 3.11 (ddd, J = 7.0, 5.3, 2.3 Hz, 16H), 2.70 (dt, J = 12.5, 6.5 Hz, 16H), 1.94 (h, J = 6.0 Hz, 16H).
JMOD ^{13}C NMR (151 MHz, Methylene Chloride-d_2) δ 142.4 (d, J = 1.9 Hz), 142.3, 128.2 (d, J = 7.0 Hz), 127.5 (d, J = 6.4 Hz), 125.5 (d, J = 2.3 Hz), 124.3 (d, J = 4.4 Hz), 122.2, 122.0, 50.4 (d, J = 7.5 Hz), 49.3, 48.9, 28.1, 22.6 (d, J = 10.1 Hz).
HRMS (DCI) m/z calcd for $C_{50}H_{59}N_4OP$ $(M)^+$: 762.4427, found : 762.4393

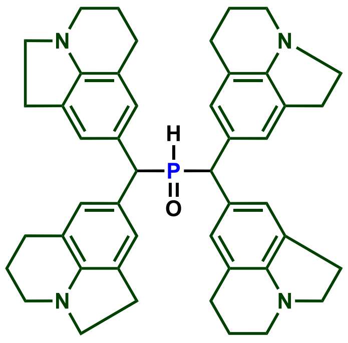

12j,78%

Following general procedure **C** using tetrafluoroborate of **1j** (0.20 mmol, 83.3 mg, 1.0 equiv.), [Na(OCP)](dioxane)$_{2.5}$ (0.24 mmol, 72.5 mg, 1.2 equiv.), H_2O (0.10 mmol, 1.8 mg) in 10 mL THF/CH_2Cl_2 (1/1) and H_2O_2 (1.0 mmol, 34.0 mg, 5.0 equiv.), purifying via silica column chromatography using petroleum ether / ethyl acetate (3/7) as eluent gave product **12j** by 78 % yield as a white solid.

^{31}P NMR (202 MHz, Methylene Chloride-d_2) δ 42.6 (dt, J = 451.6, 14.5 Hz).
^{31}P{H} NMR (202 MHz, Methylene Chloride-d_2) δ 42.6 (s).
^{1}H NMR (300 MHz, Methylene Chloride-d_2) δ 7.16 (dt, J = 451.6, 3.4 Hz, 1H), 6.92 (s, 2H), 6.79 (s, 2H), 6.74 (s, 2H), 6.62 (s, 2H), 3.80 (dd, J = 14.5, 3.4 Hz, 2H), 3.22 (td, J = 6.0, 1.4 Hz, 8H), 2.96 – 2.92 (m, 8H), 2.87 – 2.81 (m, 8H), 2.62 (m, 8H), 2.04 (m, 8H).
JMOD ^{13}C NMR (126 MHz, Methylene Chloride-d_2) δ 149.8 (d, J = 2.0 Hz), 149.6, 129.7, 129.4, 128.6 (d, J = 2.5 Hz), 127.8 (d, J = 7.6 Hz), 127.4 (d, J = 4.2 Hz), 127.2 (d, J = 7.0 Hz), 123.5 (d, J = 6.9 Hz), 122.8 (d, J = 6.5 Hz), 119.8, 119.7, 55.6 (d, J = 4.0 Hz), 50.6, 50.1, 47.7 (d, J = 9.5 Hz), 29.1 (d, J = 5.7 Hz), 24.4 (d, J = 2.6 Hz), 23.6 (d, J = 7.3 Hz).
HRMS (DCI) m/z calcd for $C_{46}H_{51}N_4OP$ $(M)^+$: 706.3801, found : 706.3788

5. Preparation of intermediates 2e and 3e

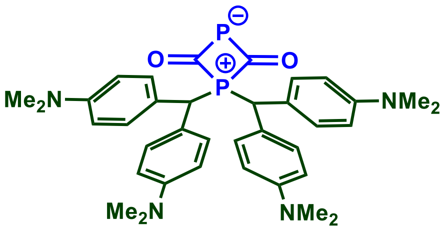

2e

4,4-dimethylaminobenzhydrylium tetrafluoroborate **1e** (0.075 mmol, 25.5 mg, 1.0 equiv.) and CD_2Cl_2 (250 µL) was successively added into a screwed cap NMR tube and filled with argon. When **1e** was completely dissolved, the NMR tube was then cooled to -60°C. After that, a solution of [Na(OCP)](dioxane)$_{2.5}$ (0.09 mmol, 27.2 mg, 1.2 equiv.) in THF-d_8 (250 µL) was added to the NMR tube and kept at this temperature for 2 minutes. Gently shake the NMR tube without removing it of the cold bath. Monitored the NMR at -60°C. The results obtained in good agreement with literature (see S80 – S85).[6]

^{31}P{H} NMR (162 MHz, THF-d_8 and methylene chloride-d_2) δ 341.8 (d, J = 36.7 Hz), 121.2 (d, J = 37.1 Hz).

^{1}**H NMR** (400 MHz, THF-d_8 and methylene chloride-d_2) δ 7.05 (d, J = 8.2 Hz, 8H), 6.63 (d, J = 8.2 Hz, 8H), 4.40 (d, J = 14.4 Hz, 2H), 2.89 (s, 24H).
JMOD ^{13}**C NMR** (101 MHz, THF-d_8 and methylene chloride-d_2) δ 207.4 (dd, J = 62.3, 19.9 Hz), 150.3, 130.7, 121.3, 112.7, 51.0 (d, J = 12.8 Hz), 40.7.

Compound **3e** was observed by NMR studies when the NMR tube contained compound **2e** was warmed up to -20°C for 15 minutes.

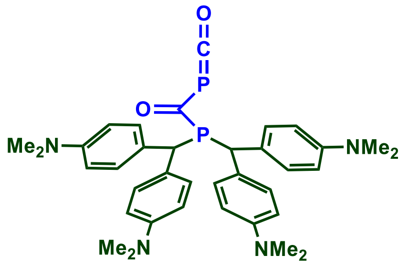

3e

^{31}**P{H} NMR** (162 MHz, THF-d_8) δ 21.2 (d, J = 167.8 Hz), -276.2 (d, J = 167.4 Hz).

6. Preparation of 10b

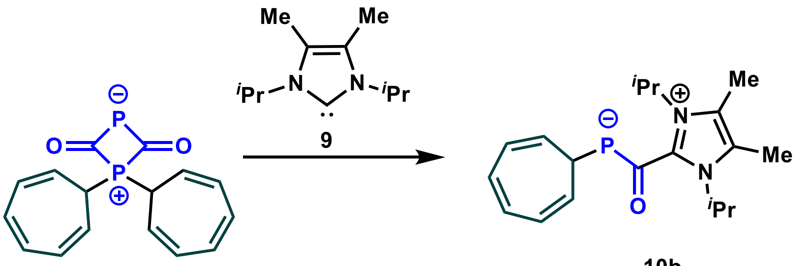

Scheme 3. Preparation of **10b**

To a stirred solution of tropylium tetrafluoroborate **1b** (1.00 mmol, 177.9 mg, 1.0 equiv.) in dry CH_2Cl_2 (5 mL) under argon at -80°C, was added dropwise a solution of [Na(OCP)](dioxane)$_{2.5}$ (1.20 mmol, 362.4 mg, 1.2 equiv.) in dry THF (5 mL) . The reaction mixture was allowed to stir for 15 minutes then followed by adding dropwise the solution of carbene **9** (3.00 mmol, 540.5 mg, 3.0 equiv.) in CH_2Cl_2 (5 mL). The resulting mixture was stirred for an additional 30 minutes at -80°C then slowly warmed up to room temperature over 1 hour. After the reaction completed as monitored by ^{31}P NMR, the solvent was removed under vacuo and the residue was washed with pentane (10 mL) and extracted with diethyl ether (20 mL) to remove the maximum of sodium tetrafluoroborate. The crude of reaction was

recrystallized in CH_2Cl_2/pentane to give red crystals of **10b** which was then identified by X-ray diffraction.

^{31}P{H} NMR (121 MHz, CD_2Cl_2) δ 95.5 (s).
^{31}P NMR (121 MHz, Methylene Chloride-d_2) δ 95.50 (q, J = 5.3 Hz).
^{1}H NMR (300 MHz, Methylene Chloride-d_2) δ 6.36 (t, J = 3.4 Hz, 2H), 6.07 – 5.90 (m, 2H), 5.22 – 5.13 (m, 2H), 3.63 – 3.55 (m, 1H), 3.46 – 3.39 (m, 2H), 2.25 (s, 6H), 1.52 (d, J = 7.1 Hz, 12H).
^{13}C NMR (126 MHz, Methylene Chloride-d_2) δ 197.2 (d, J = 58.0 Hz), 149.2 (d, J = 45.4 Hz), 133.7, 131.2, 125.9, 125.1, 51.7, 42.5 (d, J = 55.4 Hz), 22.8, 8.7.
HRMS (DCI) m/z calcd for $C_{19}H_{27}N_2OP$ $(M)^+$: 330.1861, found : 330.1866.

7. Procedure for catalytic application of phosphines in Suzuki Cross-coupling reaction

A 20 mL schlenk tube was placed under argon atmosphere and was charged with $Pd_2(dba)_3$ (2 mol %), appropriate phosphine (2 mol %) and phenyl boronic acid (1.2 equiv.). Then a solution of Cs_2CO_3 (3 equiv.) in toluene-d_8 (10^{-1} M) was added via syringe, followed by the addition of chlorobenzene (1 equiv.). The resulting suspension was then heated at 90°C for 12 hours. The yield (%) was calculated based on ^{1}H-NMR using *t*-BuOH as reference standard (see S91).

8. Procedure for detection of HPCO

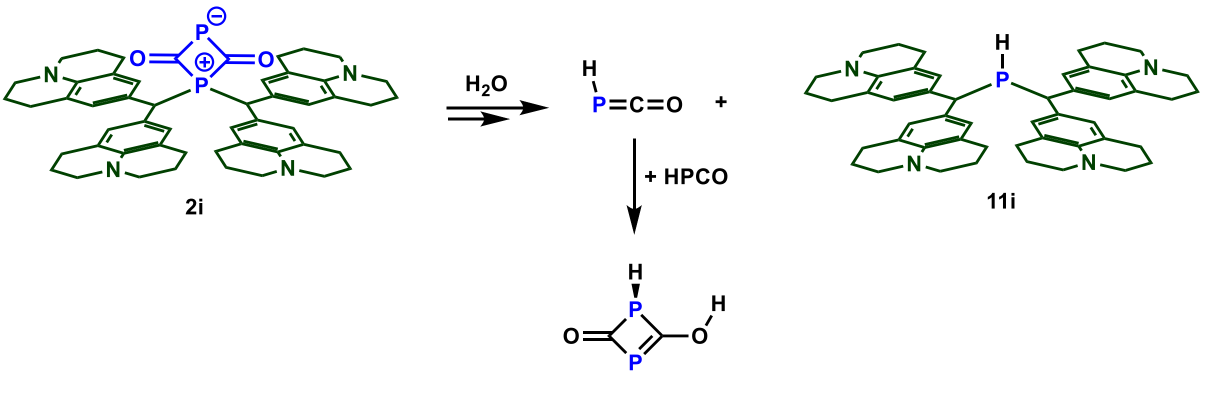

Scheme 4. Formation of HPCO in presence of water.

To a Wilmad® quick pressure valve NMR tube 5 mm diam., **1i** (20 mg, 0.045 mmol) and 250 µL of methylene chloride-d_2 was added. The NMR tube was connected to argon and cooled to -60°C, subsequently a solution of sodium phosphaethynolate [Na(OCP)](dioxane)$_{2.5}$

(14 mg, 0.045 mmol) in tetrahydrofuran-d_8 (250 µL) was added slowly *via* syringe. Once the temperature of mixture was stabilized, the NMR tube was quickly sealed with a cap and shaken carefully at this temperature until homogeneous. The NMR tube was reconnected to argon and argon-purged water (0.5 equiv., 0.023 mmol) was added. The NMR tube was kept at -60°C and subjected to the spectrometer and recorded at this temperature. $^{31}P\{H\}$ and ^{1}H NMR obtained in good agreement with literature (see S93–S94).[7]

9. Kinetic experiments

The kinetics of all investigated reactions were followed photometrically using Conventional UV-Vis-Spectroscopy and Laser Flash Photolysis. The temperature of all solutions was kept constant (20 ± 0.2°C) during all kinetic studies by using a circulating bath thermostat. At least tenfold excess of nucleophile was used in all reactions studied, so that pseudo-first-order conditions were achieved.

The kinetic experiments were performed by mixing acetonitrile solutions of the electrophiles with the minimum tetrahydrofuran solutions of [Na(OCP)](dioxane)$_{2.5}$ and monitoring the decrease of absorbances at wavelength close to the absorption maxima of electrophiles. The phosphaethynolate anion were always employed as major component (high excess) in the reactions with the electrophiles resulting in first-order kinetics. First order rate constants k_{obs} (s^{-1}) were calculated by fitting the single exponential $A_t = A_0 \exp(-k_{obs}t) + C$ (exponential decrease) to the observed time– dependent absorbance (averaged from at least 3 kinetic runs for each nucleophile concentration). The second-order rate constant (k_2) were obtained using the slopes of the linear plots of k_{obs} against the nucleophile concentration.

9.1. Conventional UV-Vis Spectroscopy

The rates of slow reaction ($t_{1/2}$ > 15-20 s) were determined by using Agilent Cary 60 UV-Vis equipped by Agilent Torlon Fiber optic probe.

9.2. Laser flash photolysis

Solutions of the precursor phosphonium salts with A_{266nm} ~ 0.5-1.0 (ca. 1 x 10^{-4} M) were irradiated with a 7 ns pulse (266 nm, 30-40 mJ/pulse) from a quadrupled Nd/YAG laser using a xenon short-arc lamp as probe light (Edinburgh LP980 Transient Absorption Spectrometer).

9.3. Determination of the Rate Constants for the reactions of phosphaethynolate anion with carbocations

Table 1. Kinetics of reactions of Na(OCP) with **1n** in acetonitrile at 20°C (λ = 491 nm)

[**1n**] mol L^{-1}	[NaOCP] mol L^{-1}	[NaOCP] / [**1n**]	k_{obs} (s^{-1})
1.22×10^{-4}	3.52×10^{-3}	29	6.13×10^{-2}
	4.03×10^{-3}	33	8.88×10^{-2}
	4.53×10^{-3}	37	1.15×10^{-1}
	5.04×10^{-3}	41	1.39×10^{-1}
	6.04×10^{-3}	50	1.66×10^{-1}

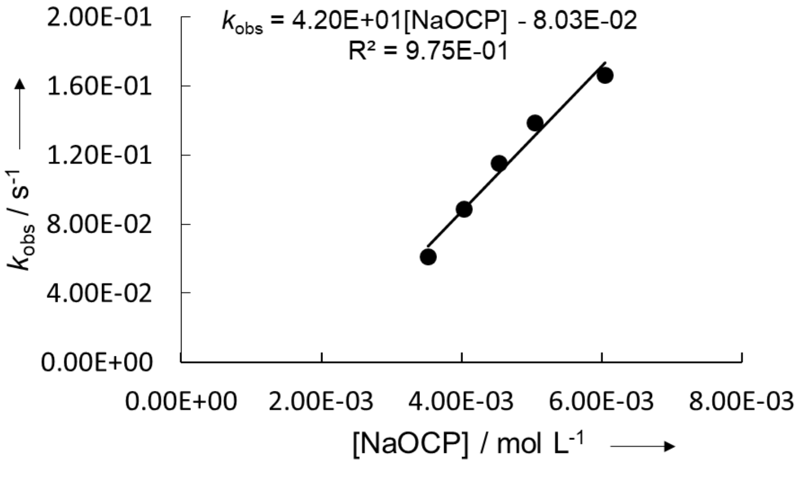

k_2 = 4.20 x 10^1 L mol^{-1} s^{-1}

Table 2. Kinetics of reactions of Na(OCP) with **1m** in acetonitrile at 20°C (λ = 395 nm)

[**1m**] mol L^{-1}	[NaOCP] mol L^{-1}	[NaOCP] / [**1m**]	k_{obs} (s^{-1})
1.26×10^{-4}	1.48×10^{-3}	12	9.90×10^{-3}
	1.65×10^{-3}	13	3.57×10^{-3}
	1.85×10^{-3}	15	9.29×10^{-3}
	2.47×10^{-3}	20	1.94×10^{-2}
	2.88×10^{-3}	23	3.22×10^{-1}

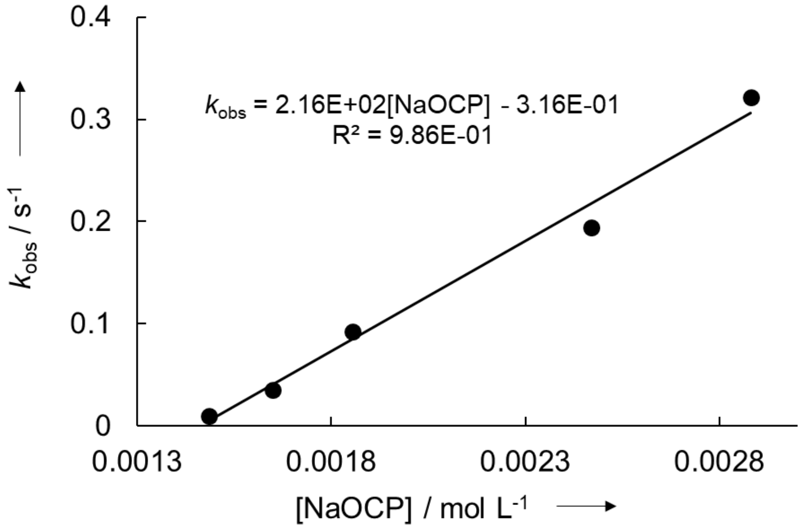

$\boldsymbol{k_2}$ **= 2.16 x 10^{2} L mol^{-1} s^{-1}**

Table 3. Kinetics of reactions of Na(OCP) with **1l** in acetonitrile at 20°C (λ = 385 nm)

[**1l**] mol L^{-1}	[NaOCP] mol L^{-1}	[NaOCP] / [**1l**]	k_{obs} (s^{-1})
4.00×10^{-5}	5.00×10^{-4}	13	2.20×10^{-1}
	6.00×10^{-4}	15	2.33×10^{-1}
	8.00×10^{-4}	20	3.06×10^{-1}

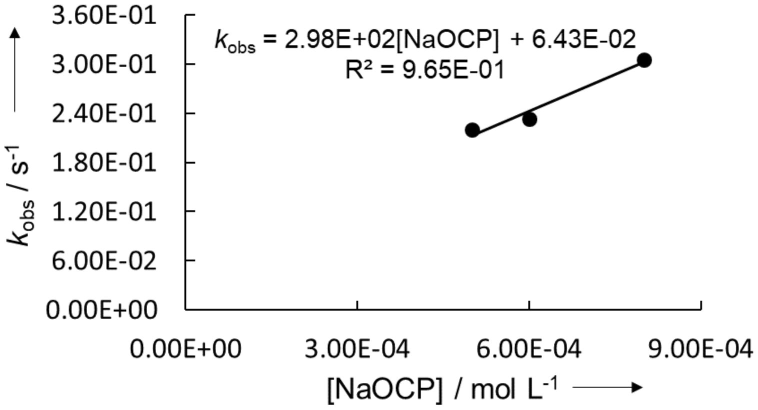

$\boldsymbol{k_2 = 2.98 \times 10^2}$ **L mol^{-1} s^{-1}**

Table 4. Kinetics of reactions of Na(OCP) with **1k** in acetonitrile at 20°C (λ = 480 nm)

[**1k**] mol L^{-1}	[NaOCP] mol L^{-1}	[NaOCP] / [**1k**]	k_{obs} (s^{-1})
3.00 x 10^{-5}	5.57 x 10^{-4}	19	7.68 x 10^{0}
	6.97 x 10^{-4}	23	1.30 x 10^{1}
	9.75 x 10^{-4}	33	1.44 x 10^{1}
	1.11 x 10^{-3}	37	1.54 x 10^{1}

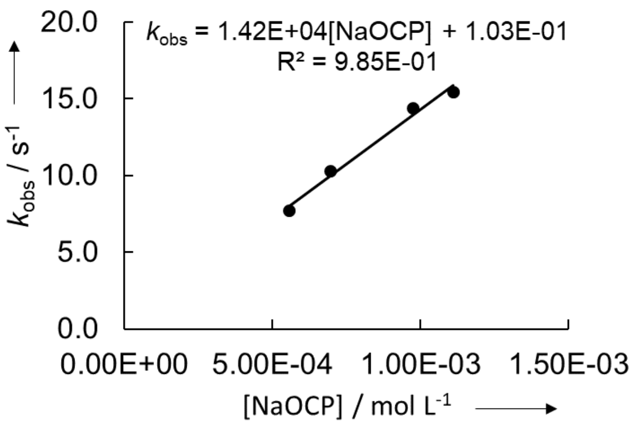

k_2 = 1.42 x 10^4 L mol^{-1} s^{-1}

Table 5. Kinetics of reactions of Na(OCP) with **1j** in acetonitrile at 20°C (λ = 635 nm)

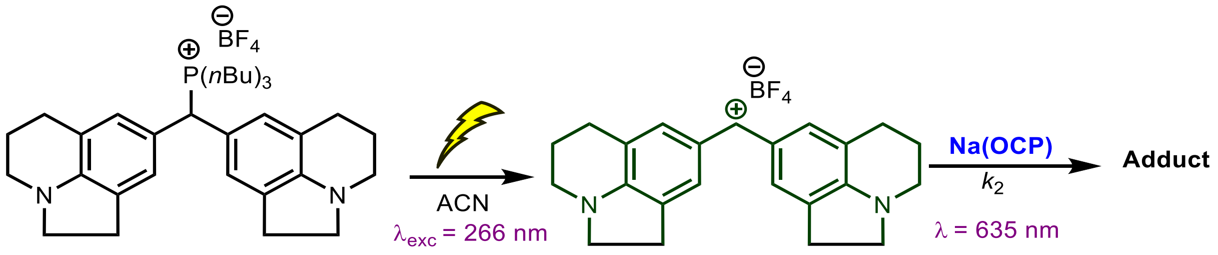

[**1j**-P(nBu)$_3$$^+BF_4$$^-$] mol L^{-1}	[NaOCP] mol L^{-1}	[NaOCP] / [**1j**-P(nBu)$_3$$^+BF_4$$^-$]	k_{obs} (s^{-1})
3.07 x 10^{-5}	4.00 x 10^{-4}	13	6.06 x 10^{3}
	6.00 x 10^{-4}	20	8.47 x 10^{3}
	8.00 x 10^{-4}	26	1.22 x 10^{4}
	1.00 x 10^{-3}	33	1.52 x 10^{4}
	1.20 x 10^{-3}	39	2.04 x 10^{4}

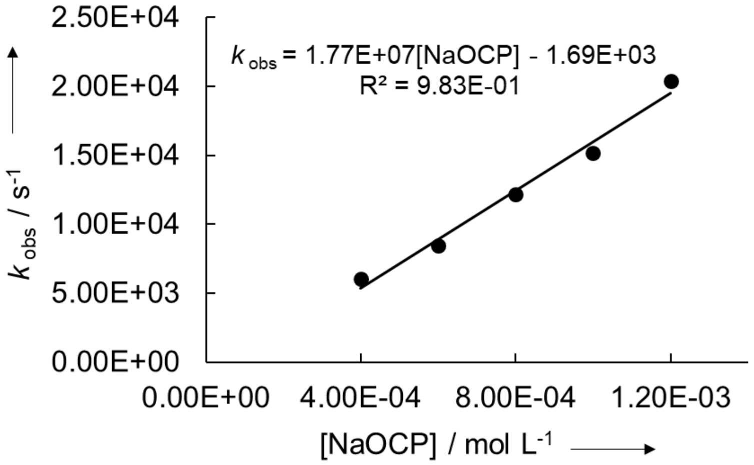

k_2 **= 1.77 x 10^7 L mol^{-1} s^{-1}**

Table 6. Kinetics of reactions of Na(OCP) with **1i** in acetonitrile at 20°C (λ = 635 nm)

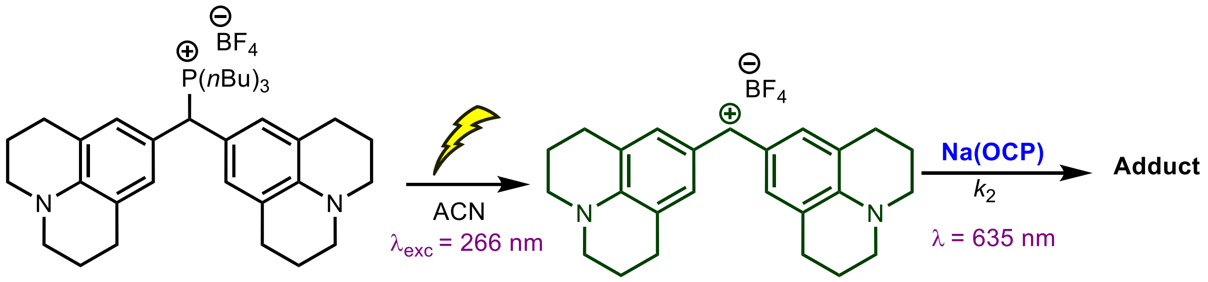

[**1i**-P(nBu)$_3^+$BF$_4^-$] mol L^{-1}	[NaOCP] mol L^{-1}	[NaOCP] / [**1i**-P(nBu)$_3^+$BF$_4^-$]	k_{obs} (s^{-1})
4.35 x 10^{-5}	8.00 x 10^{-4}	18	2.00 x 10^{4}
	9.00 x 10^{-4}	21	2.85 x 10^{4}
	1.00 x 10^{-3}	23	4.26 x 10^{4}
	1.20 x 10^{-3}	28	6.23 x 10^{4}

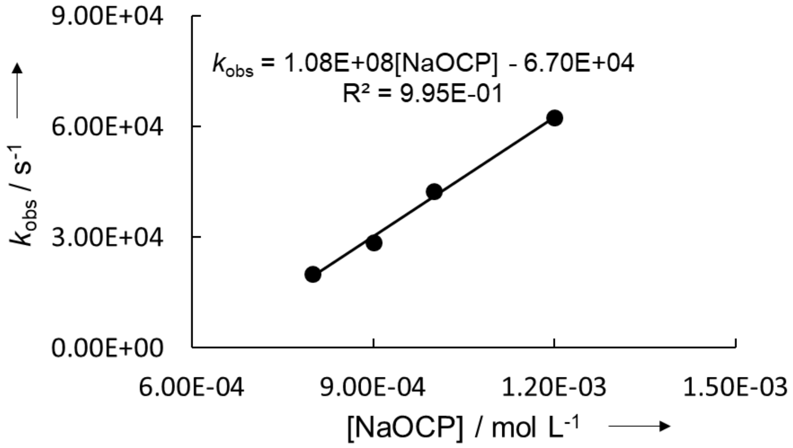

k_2 **= 1.08 x 10^{8} L mol^{-1} s^{-1}**

Table 7. Kinetics of reactions of Na(OCP) with **1h** in acetonitrile at 20°C (λ = 616 nm)

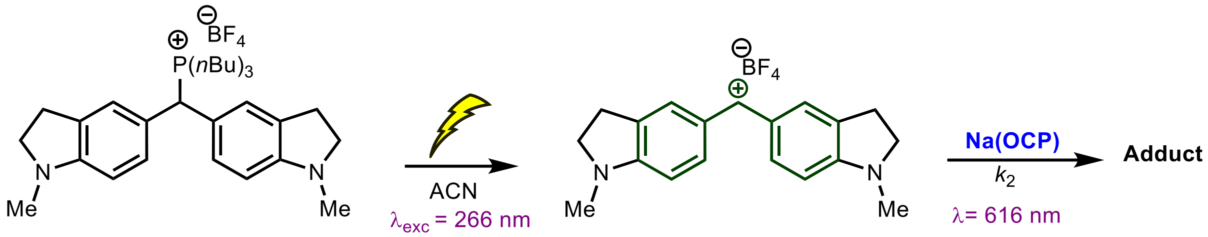

[**1h**-P(nBu)$_3^+$BF$_4^-$] mol L^{-1}	[NaOCP] mol L^{-1}	[NaOCP] / [**1h**-P(nBu)$_3^+$BF$_4^-$]	k_{obs} (s^{-1})
1.32 x 10^{-5}	2.14 x 10^{-4}	16	1.44 x 10^{4}
	4.28 x 10^{-4}	32	6.44 x 10^{4}
	5.35 x 10^{-4}	41	9.56 x 10^{4}
	6.42 x 10^{-4}	48	1.11 x 10^{5}
	8.56 x 10^{-4}	65	1.58 x 10^{5}

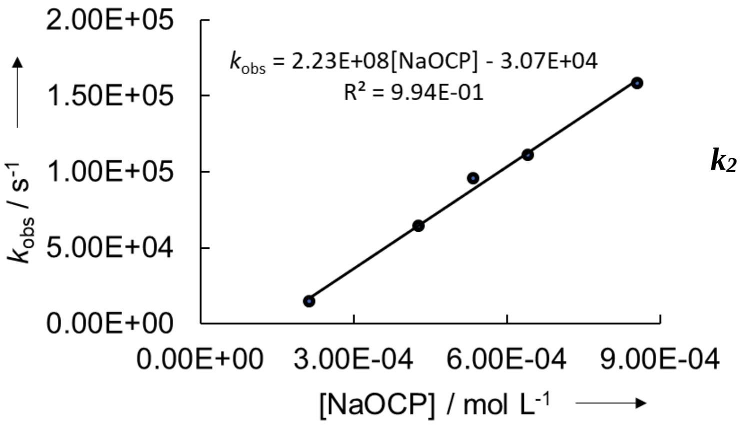

$\boldsymbol{k_2}$ **= 2.23 x 10^8 L mol^{-1} s^{-1}**

Table 8. Kinetics of reactions of Na(OCP) with **1d** in acetonitrile at 20°C (λ = 620 nm)

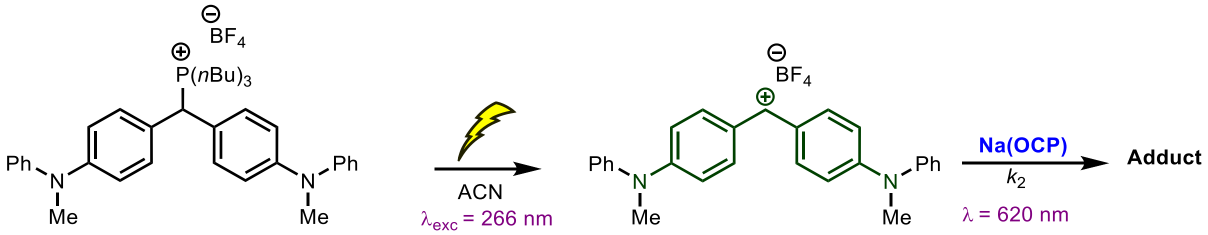

[**1d** -P(*n*Bu)$_3^+$BF$_4^-$] mol L^{-1}	[NaOCP] mol L^{-1}	[NaOCP] / [**1d** -P(*n*Bu)$_3^+$BF$_4^-$]	k_{obs} (s^{-1})
7.20 x 10^{-5}	4.56 x 10^{-4}	6	1.13 x 10^{5}
	6.84 x 10^{-4}	10	1.36 x 10^{5}
	9.12 x 10^{-4}	13	1.53 x 10^{5}
	1.14 x 10^{-3}	16	1.88 x 10^{5}

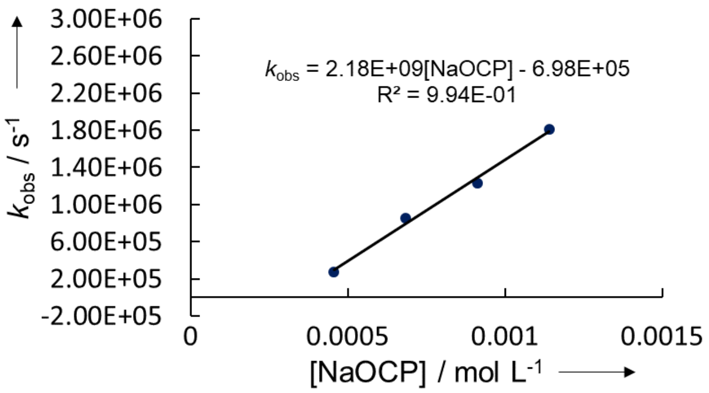

k_2 **= 2.18 x 10^9 L mol^{-1} s^{-1}**

Table 9. Kinetics of reactions of Na(OCP) with **1c** in acetonitrile at 20°C (λ = 620 nm)

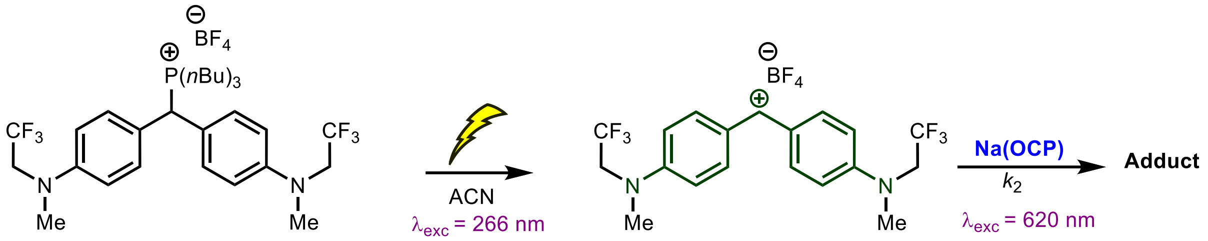

[**1c**-P(nBu)$_3^+$BF$_4^-$] mol L^{-1}	[NaOCP] mol L^{-1}	[NaOCP] / [**1c**-P(nBu)$_3^+$BF$_4^-$]	k_{obs} (s^{-1})
2.40 x 10^{-5}	2.02 x 10^{-4}	9	6.28 x 10^5
	3.03 x 10^{-4}	13	9.80 x 10^5
	4.04 x 10^{-4}	17	1.14 x 10^6

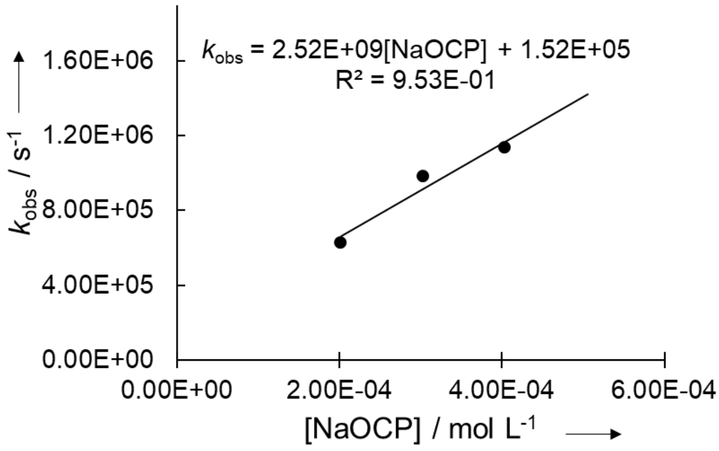

$\boldsymbol{k_2}$ **= 2.52 x 10^9 L mol^{-1} s^{-1}**

Table 10. Kinetics of reactions of Na(OCP) with **1n** in acetonitrile in presence of 15-crown-5 at 20°C (λ = 491 nm)

[**1n**] mol L^{-1}	[NaOCP] mol L^{-1}	[15-crown-5] mol L^{-1}	[NaOCP] / [**1n**]	k_{obs} (s^{-1})
1.92 x 10^{-5}	1.73 x 10^{-3}	1.04 x 10^{-2}	90	4.03 x 10^{-2}
	1.14 x 10^{-3}	6.84 x 10^{-3}	59	1.93 x 10^{-2}
	9.09 x 10^{-4}	5.45 x 10^{-3}	47	6.70 x 10^{-3}
	6.82 x 10^{-4}	4.09 x 10^{-3}	35	7.30 x 10^{-3}

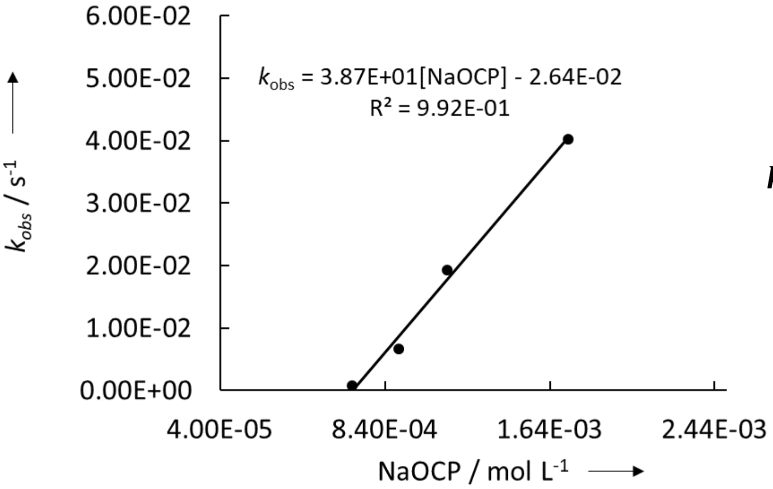

***k*$_2$ = 3.87 x 10^1 L mol^{-1} s^{-1}**

Table 11. Determination of the parameters *N* and s_N for Na(OCP) in acetonitrile at 20°C.

Electrophile	*E*	k_2 (L mol^{-1} s^{-1})	log k_2
**1n**	-17.18	4.20 x 10^1	1,62
**1m**	-16,38	2.16 x 10^2	2,33
**1l**	-15,83	2.98 x 10^2	2,47
**1k**	-13,56	1.42 x 10^4	4,15
**1j**	-10,04	1.77 x 10^7	7,25
**1i**	-9,45	1.08 x 10^8	8,03
**1h**	-8,76	2.23 x 10^8	8,35
**1d**	-5,89	2.18 x 10^9	9,34
**1c**	-3,85	2.52 x 10^9	9,40

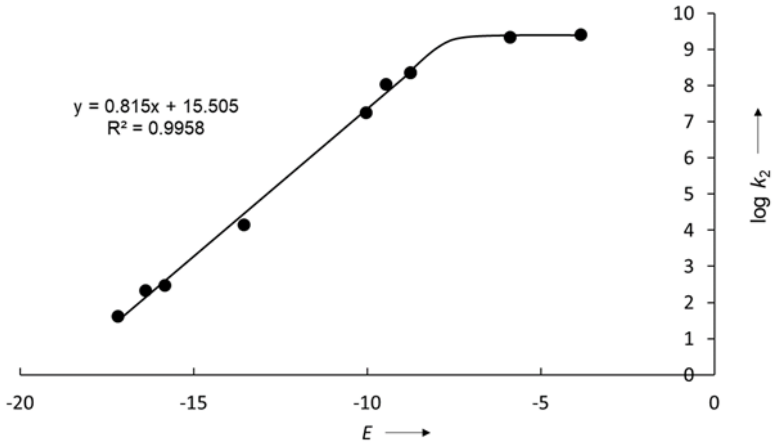

***N* = 19.02**
s_N = 0.82

10. Computational details

The computational investigations started with a thorough conformational sampling of each intermediate and transition state with Grimme's extended tight-binding method (xTB) [8] and the meta-dynamics package Conformer Rotamer Ensemble Sampling Tool (CREST).[9] Default parameters for THF solution were used together with the GFN-FF force field.[10] For very flexible molecules, the number of conformers was subsequently reduced to 30 representative structures by means of a clustering analysis.

Those structures were then optimized using the M06-2X functional [11] in combination with Grimme's D3 correction (zero damping),[12] the 6-31+G(d,p) basis set, and the SMD solvation model for THF.[13] The SuperFineGrid was used within the Gaussian program [14] for the numerical integration of the density. Vibrational analysis verified that each structure was a minimum or a transition state. Following the intrinsic reaction coordinates (IRC) confirmed that all transition states connected the corresponding minima on the potential energy surface. Thermal corrections were obtained from unscaled harmonic vibrational frequencies at the same level of theory for a standard state of 1 mol L^{-1} and 298.15 K. Entropic contributions to free energies were obtained from partition functions evaluated with Grimme's quasi-harmonic approximation.[15] This method employs the free-rotor approximation for all frequencies below 100 cm^{-1}, the rigid-rotor-harmonic-oscillator approximation for all frequencies above 100 cm^{-1}, and a damping function to interpolate between the two expressions.

Accurate electronic energies were obtained for the optimized structures relying within the ORCA program [16] on the DSD-PBEP86 double-hybrid functional,[17] Grimme's D3 correction with Becke-Johnson damping,[18] the def2-QZVPP in combination with the corresponding auxiliary basis sets,[19] and the SMD solvation model for THF.

The final Cartesian coordinates and calculated energies for the most stable structures are provided as an additional archive file.

Table 12. Control Calculations and Validation of the Computational Method

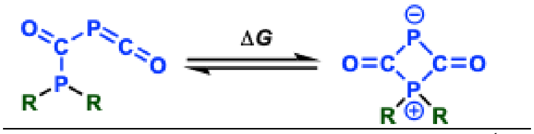

R = Me	–36 kJ mol^{-1}
R = Ph	–21 kJ mol^{-1}
R = C_7H_7	–39 kJ mol^{-1}
R = $CH(C_6H_4NMe_2)_2$	–42 kJ mol^{-1}

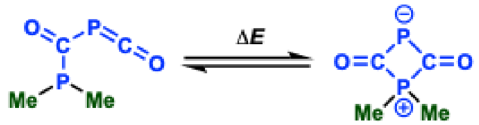

DLPNO-CCSD(T)	–25 kJ mol^{-1}
DSD-BLYP	–32 kJ mol^{-1}
B2PLYP	–32 kJ mol^{-1}
B2GP-PLYP	–34 kJ mol^{-1}
M06-2X	–17 kJ mol^{-1}
B3LYP	–32 kJ mol^{-1}
ωB97X-D3	–43 kJ mol^{-1}
ωB97X-V	–37 kJ mol^{-1}
PW6B95	–38 kJ mol^{-1}
DSD-BLYP Gas	+1 kJ mol^{-1}
DSD-BLYP Benzene	–20 kJ mol^{-1}
DSD-BLYP THF	–32 kJ mol^{-1}
DSD-BLYP Water	–49 kJ mol^{-1}
DSD-BLYP Pentane	–16 kJ mol^{-1}

11. Crystallographic data

Crystallographic data were collected at low temperature (193(2) K) on a Bruker APEX II Quazar diffractometer equipped with a 30 W air-cooled microfocus for **13a** and on a Bruker D8 VENTURE diffractometer equipped with a PHOTON III detector for **10b** and **12b**, using MoK_α radiation (λ = 0.71073 Å). Phi and Omega scans were performed for data collection. An empirical absorption correction was applied [20] and the structures were solved by intrinsic phasing method (ShelXT) [21]. All non-hydrogen atoms were refined anisotropically by means of least-squares procedures on F^2 with ShelXL [22]. For **13a**, the investigated crystal was twinned, the twin law was identified with the program PLATON [23] and the HKLF5 file was used for structure refinement. The domain fractions were refined to 0.74 and 0.26.

Table 13. Crystal Data, Data Collection, and Structure Refinement for **10b**, **13a** and **12b**.

ID	**10b**	**13a**	**12b**
formula	$C_{19}H_{27}N_2OP$	$C_{45}H_{45}O_7P$	$C_{14}H_{15}OP$
M_r	415.32	728.78	230.23
crystal system	triclinic	triclinic	monoclinic
space group	*P* -1	*P* -1	C2/c
a (Å)	10.8087(8)	6.8013(13)	18.3122(8)
b (Å)	11.2182(8)	13.832(3)	5.9341(2)
c (Å)	11.3752(8)	20.961(4)	22.4465(11)
α (°)	61.101(2)	102.003(5)	90
β (°)	89.292(3)	99.318(6)	97.4662(18)
γ (°)	66.734(2)	98.164(7)	90
V ($Å^3$)	1081.66(14)	1871.5(6)	2418.50(18)
Z	2	2	8
ρ_{calc} (mg m^{-3})	1.275	1.293	1.265
μ (mm^{-1})	0.386	0.126	0.203
F(000)	440	772	976
crystal size (mm^3)	0.24 x 0.20 x 0.07	0.12 x 0.12 x 0.02	0.24 x 0.18 x 0.16
T/K	193(2)	193(2)	193(2)
measd reflns	22682	49470	37622
Unique reflns (Rint)	5322 (0.0325)	49470 (0.1230)	2960 (0.0286)
Data/restraints/parameters	5322 / 0 / 241	49470 / 589 / 631	2960 / 222 / 204
GOF on F^2	1.115	1.021	1.133
R_1[a] [I>2σ(I)]	0.0594	0.0970	0.0445
wR_2[b] [all data]	0.1698	0.2388	0.1009

[a] $R_1 = \Sigma||F_o| - |F_c||/ \Sigma |F_o|$. [b] $wR_2 = [\Sigma[w(F_o^2 - F_c^2)^2] / \Sigma [w(F_o^2)^2]]^{1/2}$.

12. References

[1] a) F. F Puschmann, D. Stein, D. Heift, C. Hendriksen, Z.A. Gal, H.F. Grützmacher, H. Grützmacher, *Angew.Chem.Int.Ed.* **2011**, *50*, 8420–8423. b) D. Heift, Z. Benkő, H. Grützmacher, *Dalton Trans.* **2014**, *43*, 831–840.

[2] a) H. Mayr, T. Bug, M. F. Gotta, N. Hering, B. Irrgang, B. Janker, B. Kempf, R. Loos, A. R. Ofial, G. Remennikov, H. Schimmel, *J. Am. Chem. Soc.* **2001**, *123*, 9500–9512. b) R.J. Mayer, N. Hampel, P. Mayer, A. R. Ofial, H. Mayr, *Eur. J. Org. Chem.* **2009**, 412–421.

[3] D. Richter, N. Hampel, T. Singer, A. R. Ofial, H. Mayr, *Eur. J. Org. Chem.* **2009**, 3203–3211.

[4] S. T. A. Berger, F. H. Seeliger, F. Hofbauer, H. Mayr, *Org. Biomol. Chem.* **2007**, *5*, 3020.

[5] B. Kempf, H. Mayr, *Chemistry – A European Journal* **2005**, *11*, 917–927.

[6] M. S. Kelvin, A. R. Jupp, R. Suter, H. Grützmacher, D. W. Stephan, *Angew.Chem.Int.Ed.* **2017**, *56*, 14174–14177.

[7] A. Hinz, M. Sc. R. Labbow, C. Rennick, A. Schulz, J. M. Goicoechea, *Angew.Chem.Int.Ed.* **2017**, *56*, 3911–3915.

[8] C. Bannwarth, E. Caldeweyher, S. Ehlert, A. Hansen, P. Pracht, J. Seibert, S. Spicher, S. Grimme, *Wiley Interdiscip. Rev.: Comput. Mol. Sci.* **2021**, *11*, e1493.

[9] P. Pracht, F. Bohle, S. Grimme, *Phys. Chem. Chem. Phys.* **2020**, *22*, 7169–7192.

[10] S. Spicher, S. Grimme, *Angew.Chem.Int.Ed.* **2020**, *59*, 15665–15673.

[11] Y. Zhao, D. G. Truhlar, *Theor. Chem. Acc.* **2008**, *120*, 215–241.

[12] S. Grimme, J. Antony, S. Ehrlich, H. Krieg, *J. Chem. Phys.* **2010**, *132*, 154104.

[13] A. V. Marenich, C. J. Cramer, D. G. Truhlar, *J. Phys. Chem. B* **2009**, *113*, 6378–6396.

[14] Gaussian16, Revision C.01. M. J. Frisch, G. W. Trucks, H. B. Schlegel, G. E. Scuseria, M. A. Robb, J. R. Cheeseman, G. Scalmani, V. Barone, G. A. Petersson, H. Nakatsuji, X. Li, M. Caricato, A. V. Marenich, J. Bloino, B. G. Janesko, R. Gomperts, B. Mennucci, H. P. Hratchian, J. V. Ortiz, A. F. Izmaylov, J. L. Sonnenberg, Williams, F. Ding, F. Lipparini, F. Egidi, J. Goings, B. Peng, A. Petrone, T. Henderson, D. Ranasinghe, V. G. Zakrzewski, J. Gao, N. Rega, G. Zheng, W. Liang, M. Hada, M. Ehara, K. Toyota, R. Fukuda, J. Hasegawa, M. Ishida, T. Nakajima, Y. Honda, O. Kitao, H. Nakai, T. Vreven, K. Throssell, J. A. Montgomery Jr., J. E. Peralta, F. Ogliaro, M. J. Bearpark, J. J. Heyd, E. N. Brothers, K. N. Kudin, V. N. Staroverov, T. A. Keith, R. Kobayashi, J. Normand, K. Raghavachari, A. P. Rendell, J. C. Burant, S. S. Iyengar, J. Tomasi, M. Cossi, J. M. Millam, M. Klene, C. Adamo, R. Cammi, J. W. Ochterski, R. L. Martin, K. Morokuma, O. Farkas, J. B. Foresman, D. J. Fox,Wallingford, CT, **2016**.

[15] S. Grimme, *Chem. Eur. J.* **2012**, *18*, 9955–9964.

[16] a) F. Neese, *Wiley Interdiscip. Rev. Comput. Mol. Sci.* **2022**, *12*, e1606; b) F. Neese, *Wiley Interdiscip. Rev. Comput. Mol. Sci.* **2018**, *8*, e1327.

[17] S. Kozuch, J. M. L. Martin, *Phys. Chem. Chem. Phys.* **2011**, *13*, 20104–20107.

[18] S. Grimme, S. Ehrlich, L. Goerigk, *J. Comput. Chem.* **2011**, *32*, 1456–1465.

[19] a) F. Weigend, R. Ahlrichs, *Phys. Chem. Chem. Phys.* **2005**, *7*, 3297–3305; b) F. Weigend, *Phys. Chem. Chem. Phys.* **2006**, *8*, 1057–1065; c) A. Hellweg, C. Hättig, S. Höfener, W. Klopper, *Theor. Chem. Acc.* **2007**, *117*, 587–597.

[20] Bruker, SADABS, Bruker AXS Inc., Madison, Wisconsin, USA, **2008**.

[21] ShelXT, G. M. Sheldrick, University of Göttingen, *Acta Crystallogr. Sect. A*, **2015**, *71*, 3–8.

[22] ShelXT, G. M. Sheldrick, University of Göttingen, *Acta Crystallogr. Sect. C*, **2015**, *71*, 3–8.

[23] PLATON, A. L. Spek, University of Utrecht, *Acta Crystallogr. Sect. C*, **2015**, *71*, 9–18.

13. Copies of NMR Spectra and HRMS

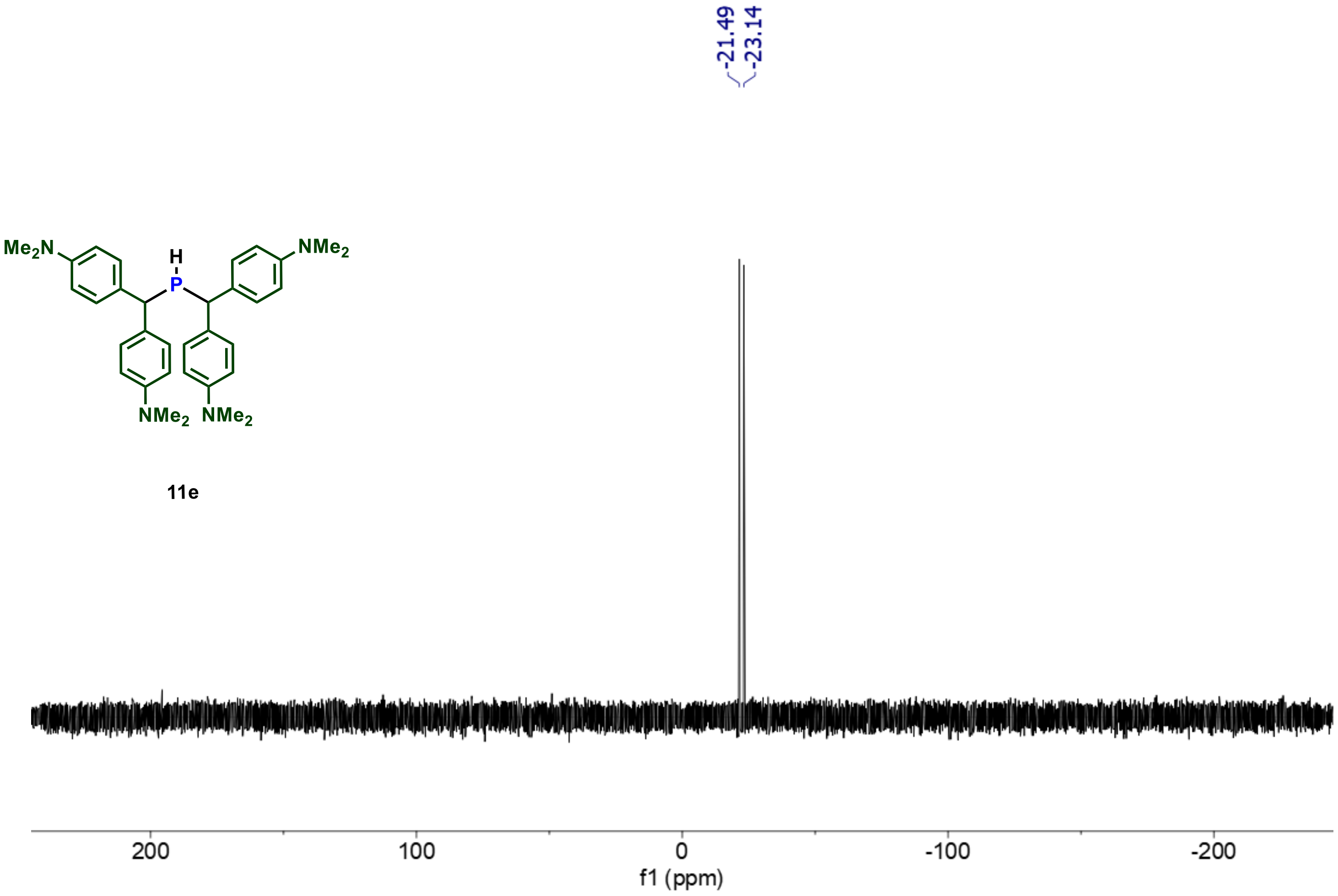

Figure 1. ^{31}P NMR (121 MHz, Chloroform-*d*) of **11e**

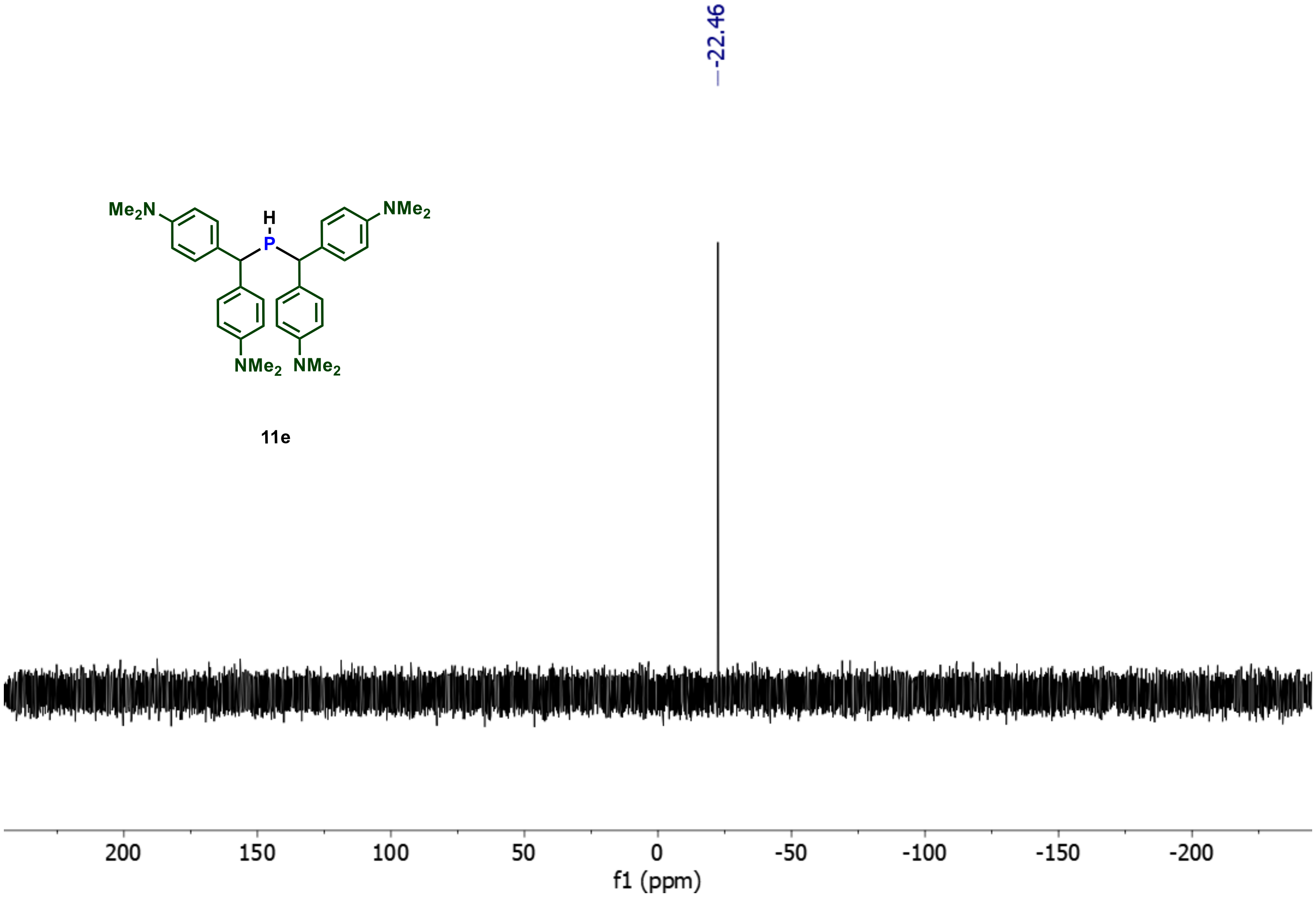

Figure 2. ^{31}P{H} NMR (121 MHz, Chloroform-*d*) of **11e**

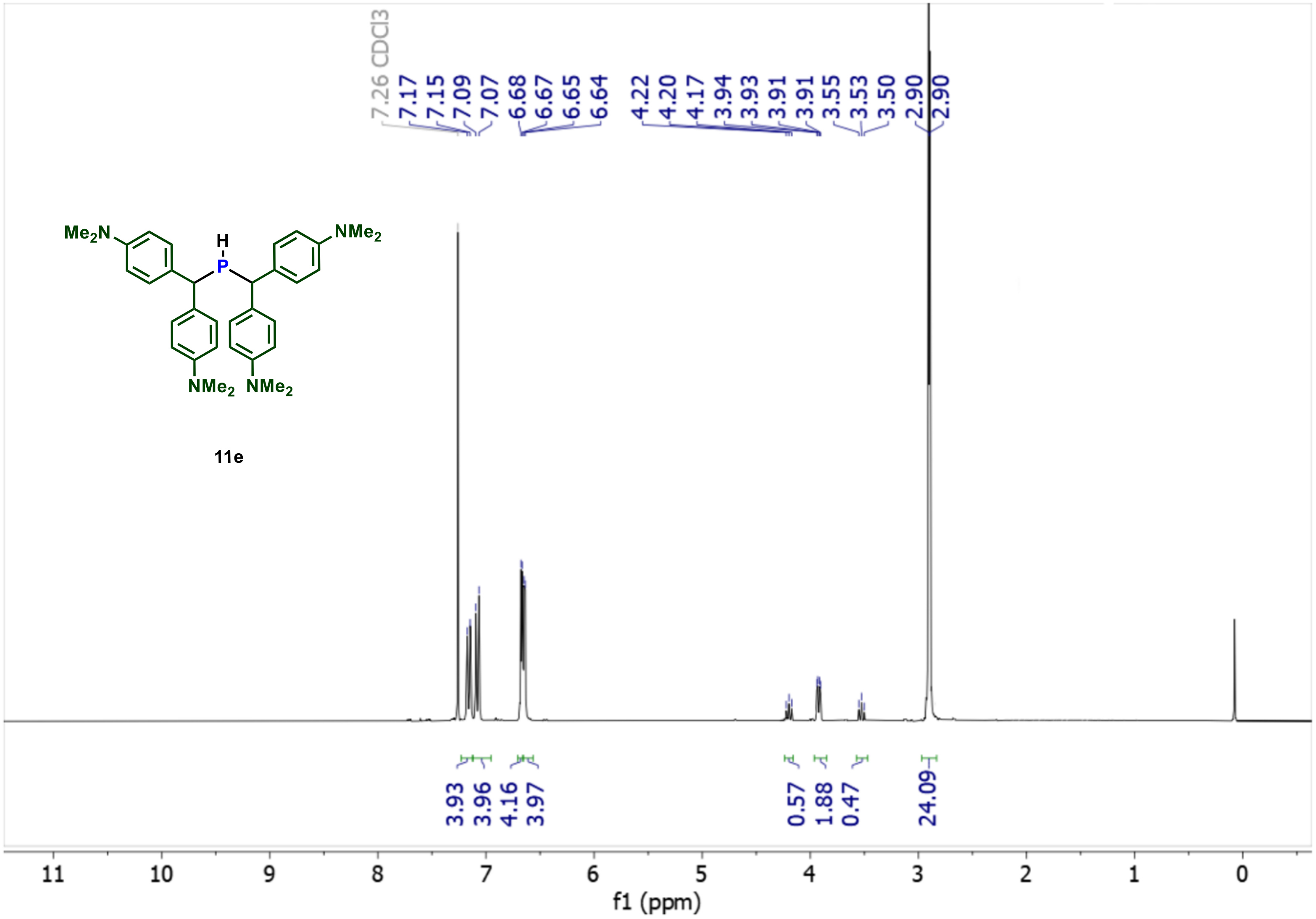

Figure 3. ^{1}H NMR (300 MHz, Chloroform-*d*) of **11e**

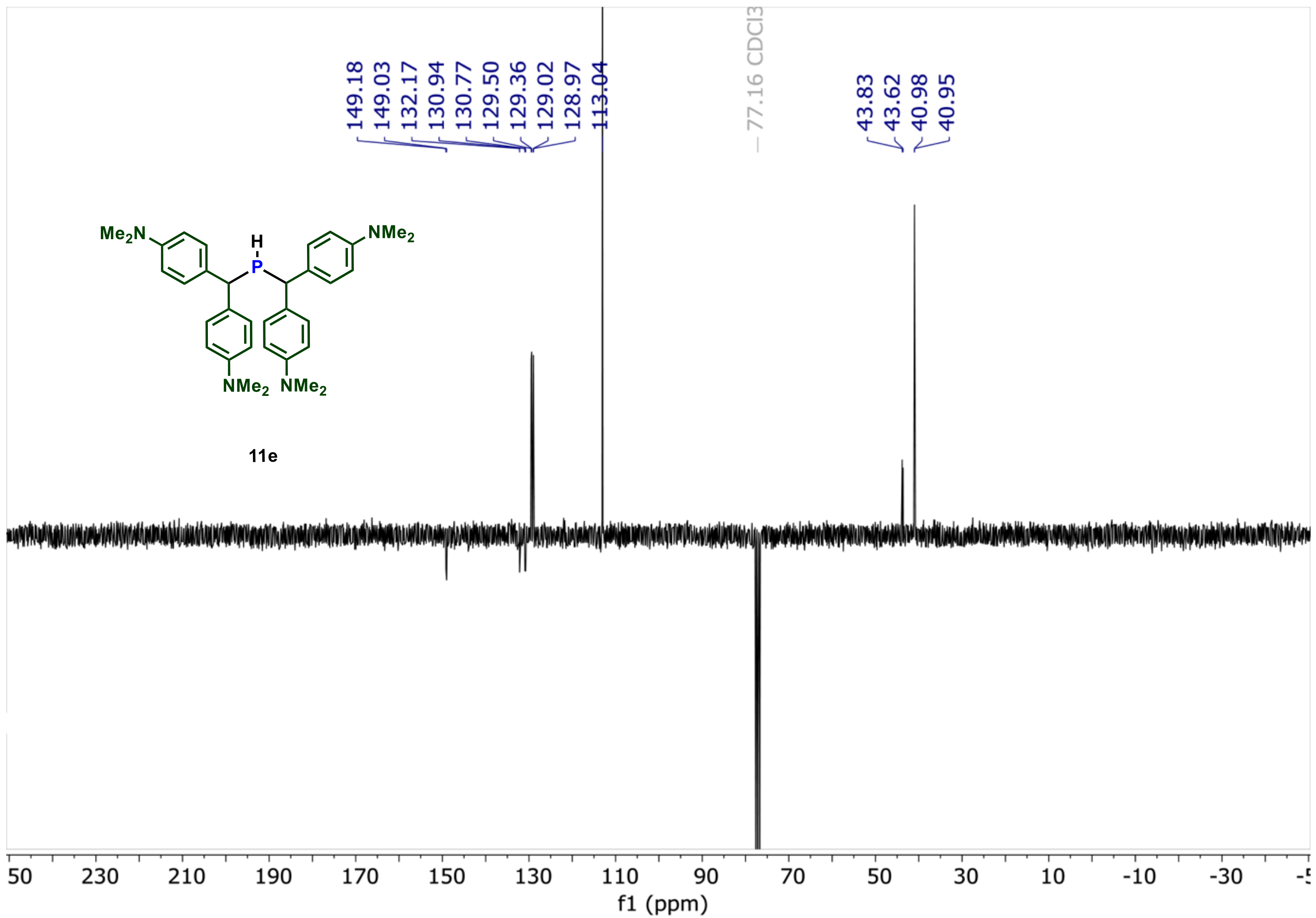

Figure 4. JMOD ^{13}C NMR (75 MHz, Chloroform-*d*) of **11e**

Single Mass Analysis

Tolerance = 5.0 PPM / DBE: min = -1.5, max = 80.0
Element prediction: Off

Monoisotopic Mass, Even Electron Ions
2077 formula(e) evaluated with 11 results within limits (all results (up to 1000) for each mass)
Elements Used:
C: 0-100 H: 0-100 N: 0-10 O: 0-10 P: 0-2

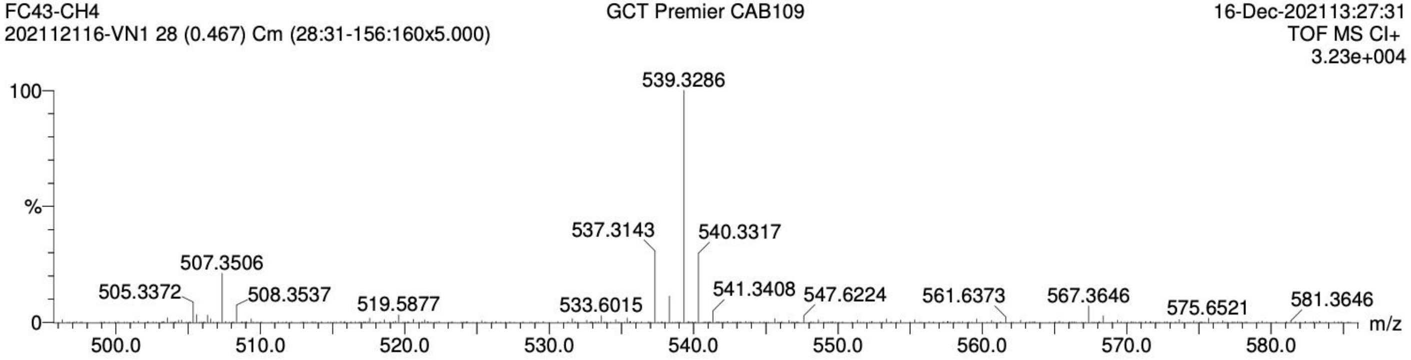

Minimum:				-1.5		
Maximum:		1.4	5.0	80.0		

Mass	Calc. Mass	mDa	PPM	DBE	i-FIT	Formula
539.3286	539.3290	-0.4	-0.7	10.5	403.0	C33 H48 O4 P
	539.3282	0.4	0.7	-1.5	209.4	C17 H48 N8 O9 P
	539.3280	0.6	1.1	6.5	48.6	C27 H49 N4 O3 P2
	539.3292	-0.6	-1.1	2.5	44.3	C23 H47 N4 O10
	539.3274	1.2	2.2	15.5	676.1	C35 H43 N2 O3
	539.3304	-1.8	-3.3	15.5	539.9	C34 H44 N4 P
	539.3267	1.9	3.5	1.5	28.3	C26 H53 O7 P2
	539.3306	-2.0	-3.7	7.5	27.8	C24 H43 N8 O6
	539.3265	2.1	3.9	3.5	67.8	C19 H43 N10 O8
	539.3263	2.3	4.3	11.5	177.9	C29 H44 N6 O2 P
	539.3312	-2.6	-4.8	-1.5	275.2	C16 H49 N10 O6 P2

Figure 5. **HRMS** (DCI) for **11e**

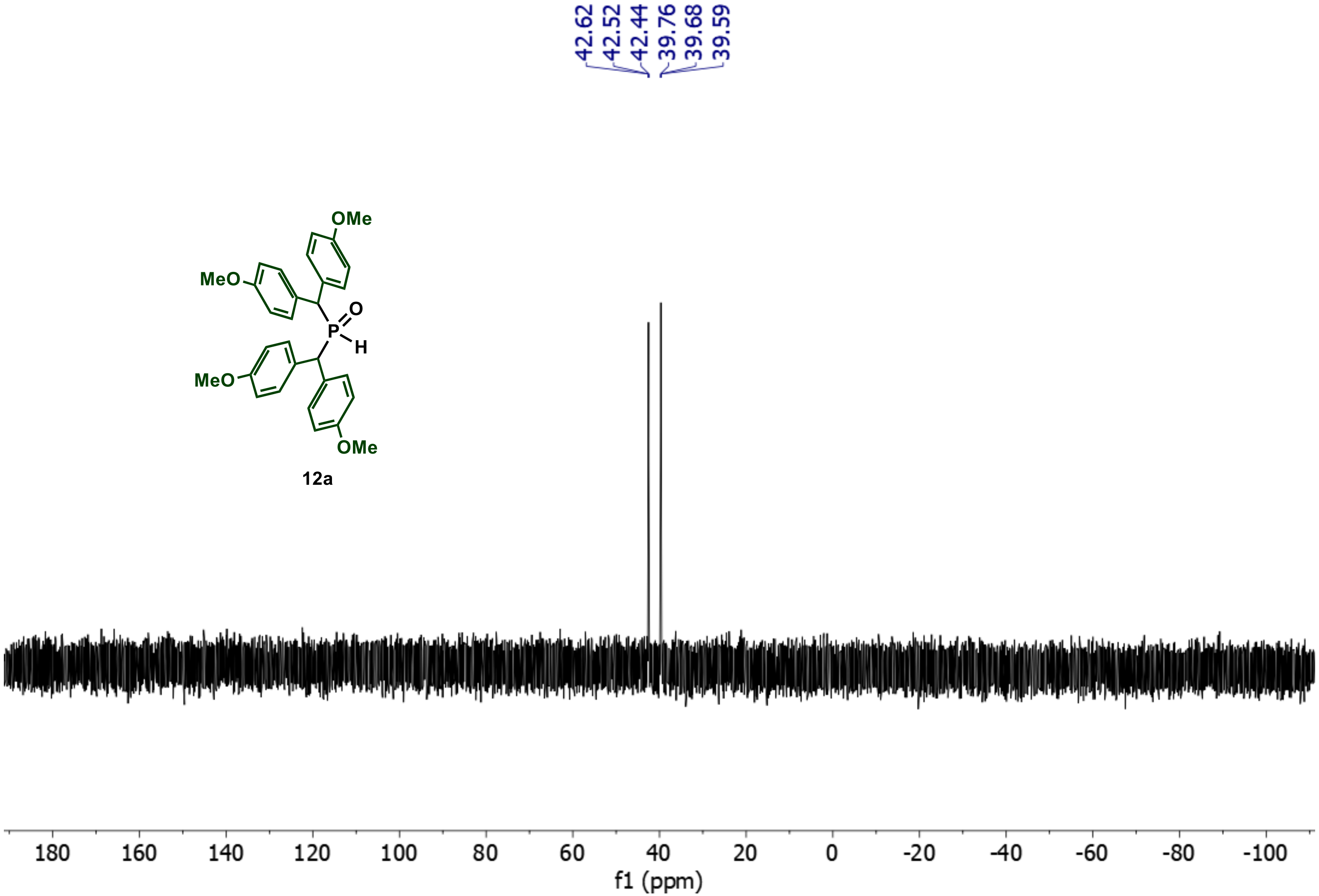

Figure 6. ^{31}P NMR (162 MHz, Methylene Chloride-d_2) of **12a**

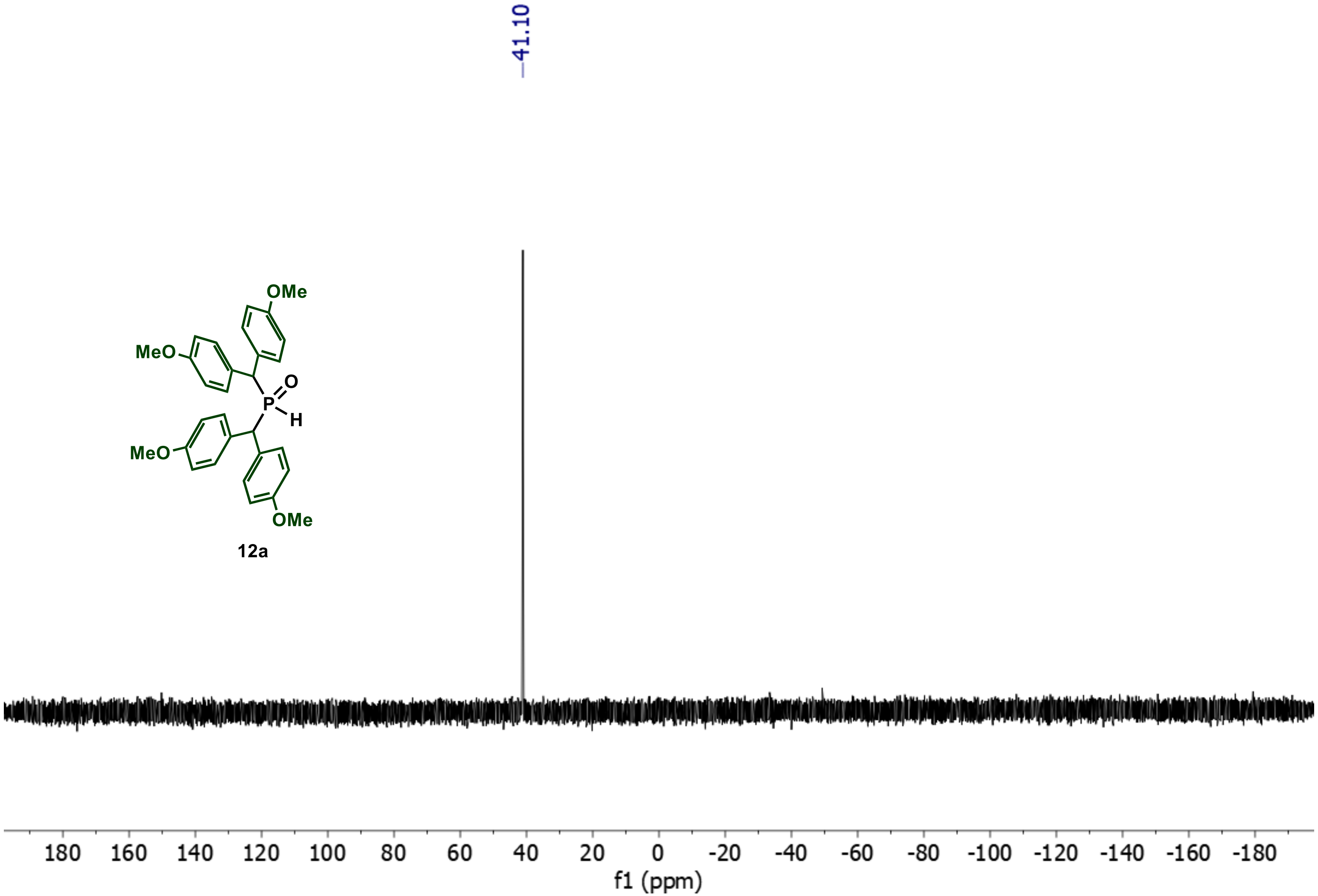

Figure 7. $^{31}P\{H\}$ NMR (162 MHz, Methylene Chloride-d_2) of **12a**

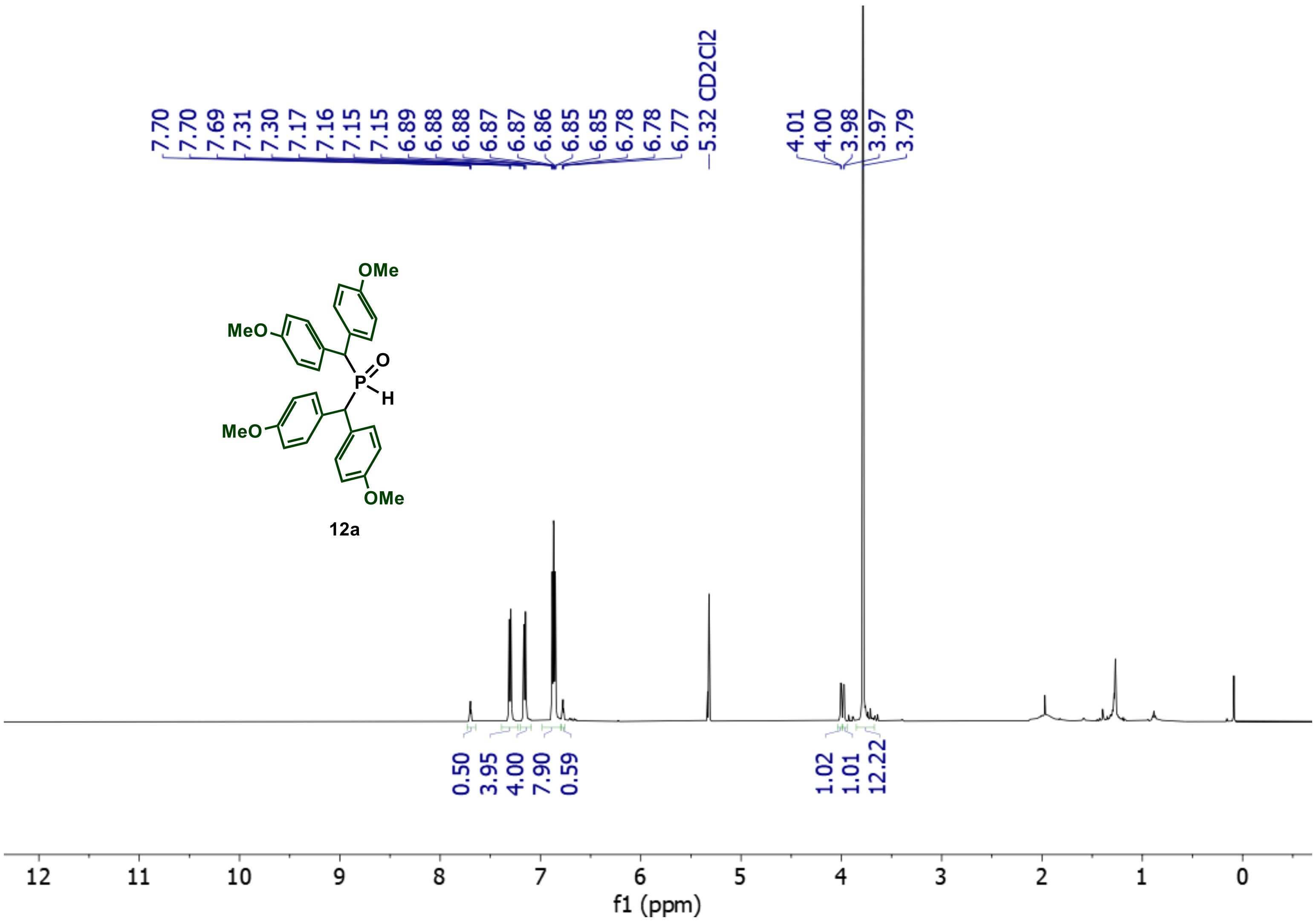

Figure 8. ^{1}H NMR (500 MHz, Methylene Chloride-d_2) of **12a**

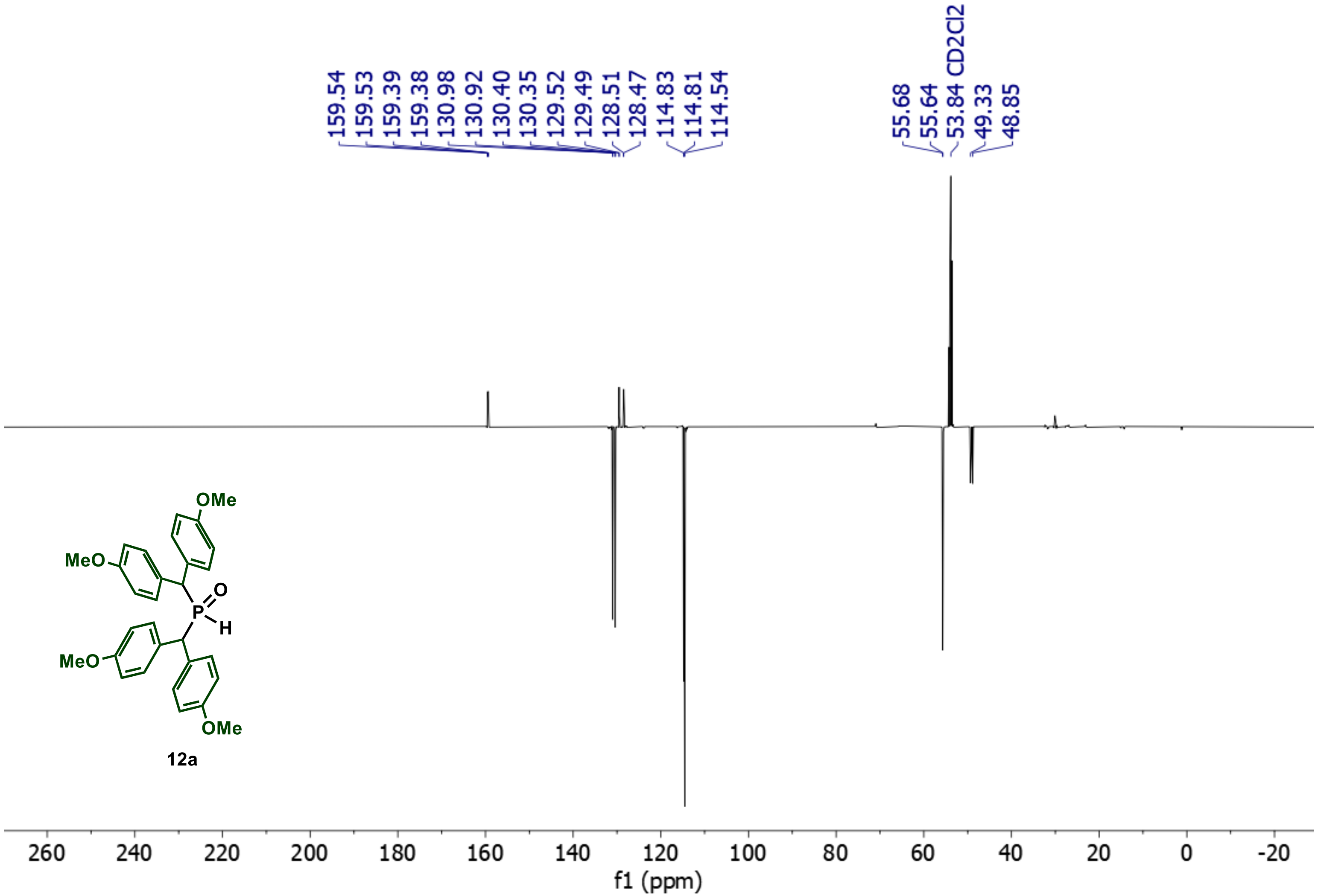

Figure 9. JMOD ^{13}C NMR (126 MHz, Methylene Chloride-d_2) of **12a**

Elemental Composition Report

Single Mass Analysis

Tolerance = 5.0 PPM / DBE: min = -1.5, max = 80.0
Element prediction: Off

Monoisotopic Mass, Odd and Even Electron Ions
77 formula(e) evaluated with 2 results within limits (all results (up to 1000) for each mass)
Elements Used:
C: 0-100 H: 0-100 O: 0-5 P: 0-1

DCI-CH4 GCT Premier CAB109 25-Oct-202210:09:25
20221025-VN151-F5 27 (0.450) Cm (25:34-80:88x5.000) TOF MS CI+
4.53e+003

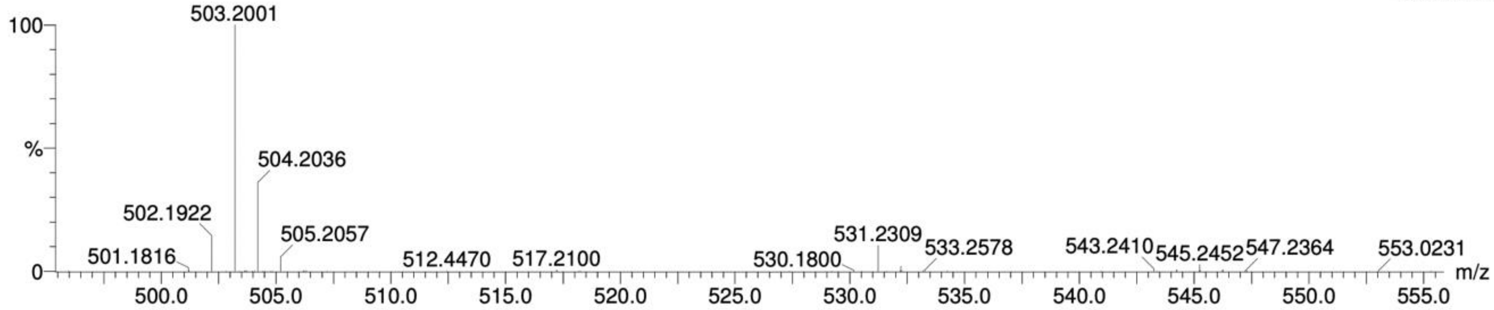

Minimum:				-1.5		
Maximum:		1.5	5.0	80.0		
Mass	Calc. Mass	mDa	PPM	DBE	i-FIT	Formula
502.1922	502.1933	-1.1	-2.2	25.0	2690.0	C37 H26 O2
	502.1909	1.3	2.6	16.0	2776.0	C30 H31 O5 P

Figure 10. **HRMS** (DCI) for **12a**

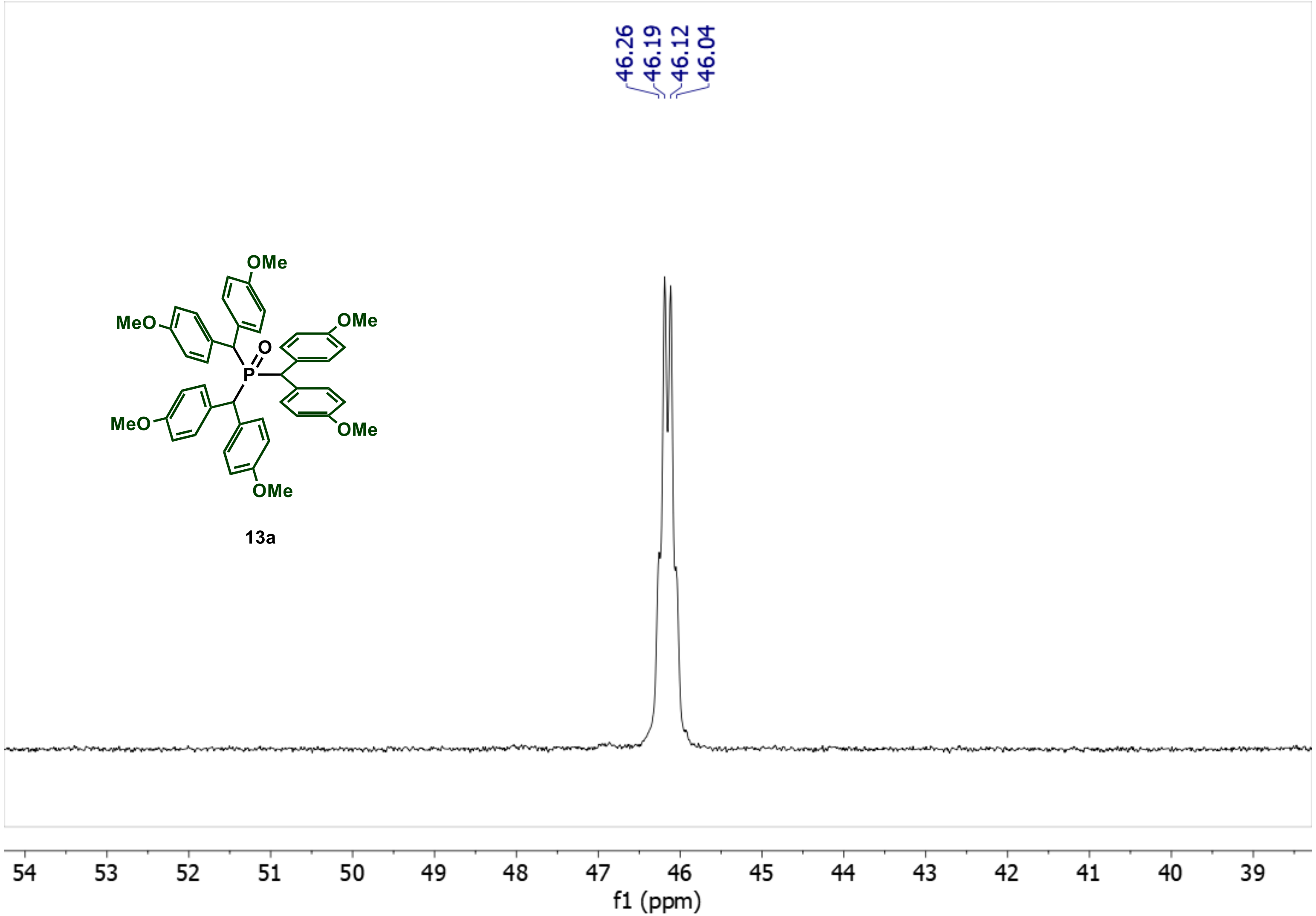

Figure 11. ^{31}P NMR (162 MHz, Methylene Chloride-d_2) of **13a**

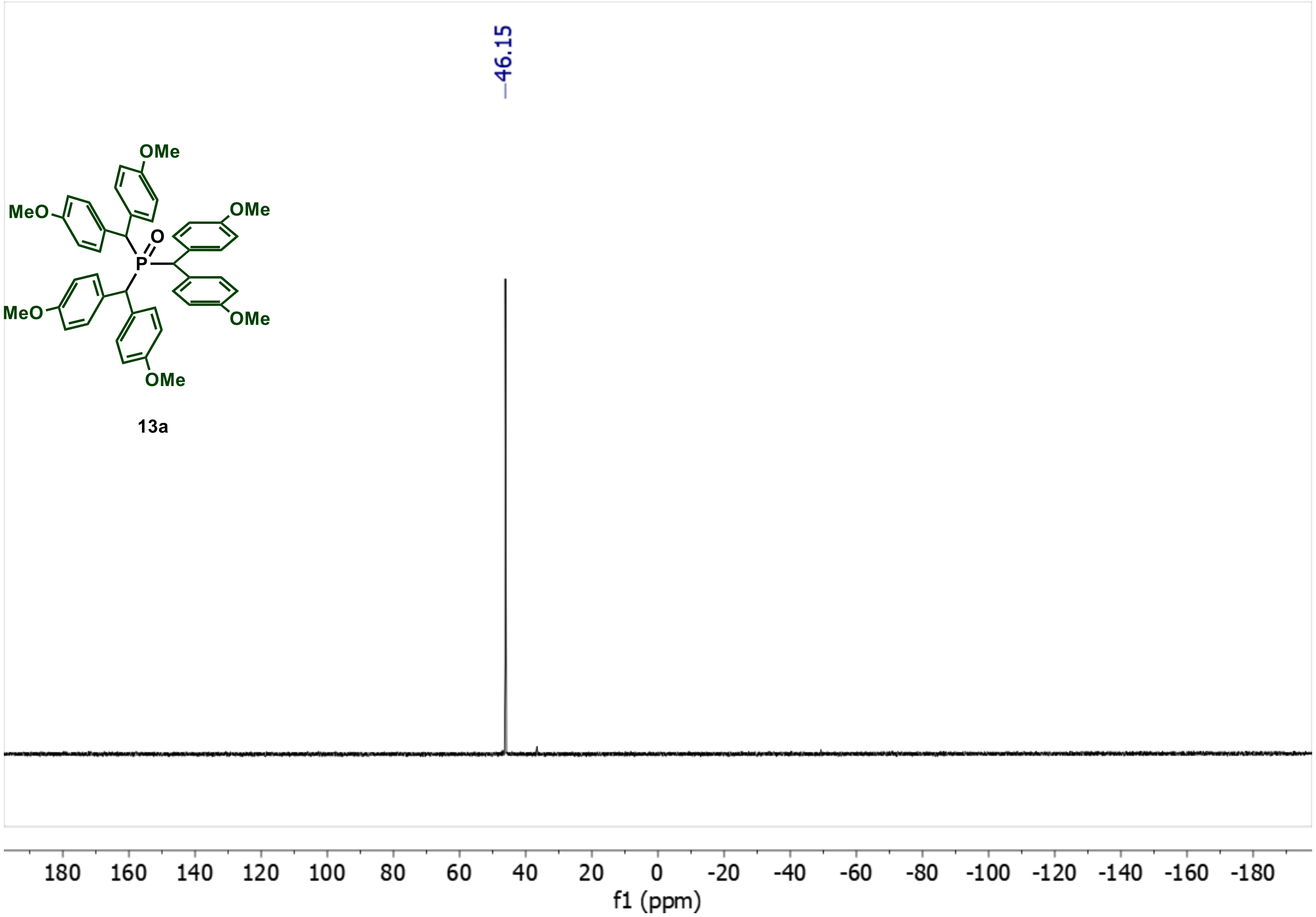

Figure 12. $^{31}P\{H\}$ NMR (162 MHz, Methylene Chloride-d_2) of **13a**

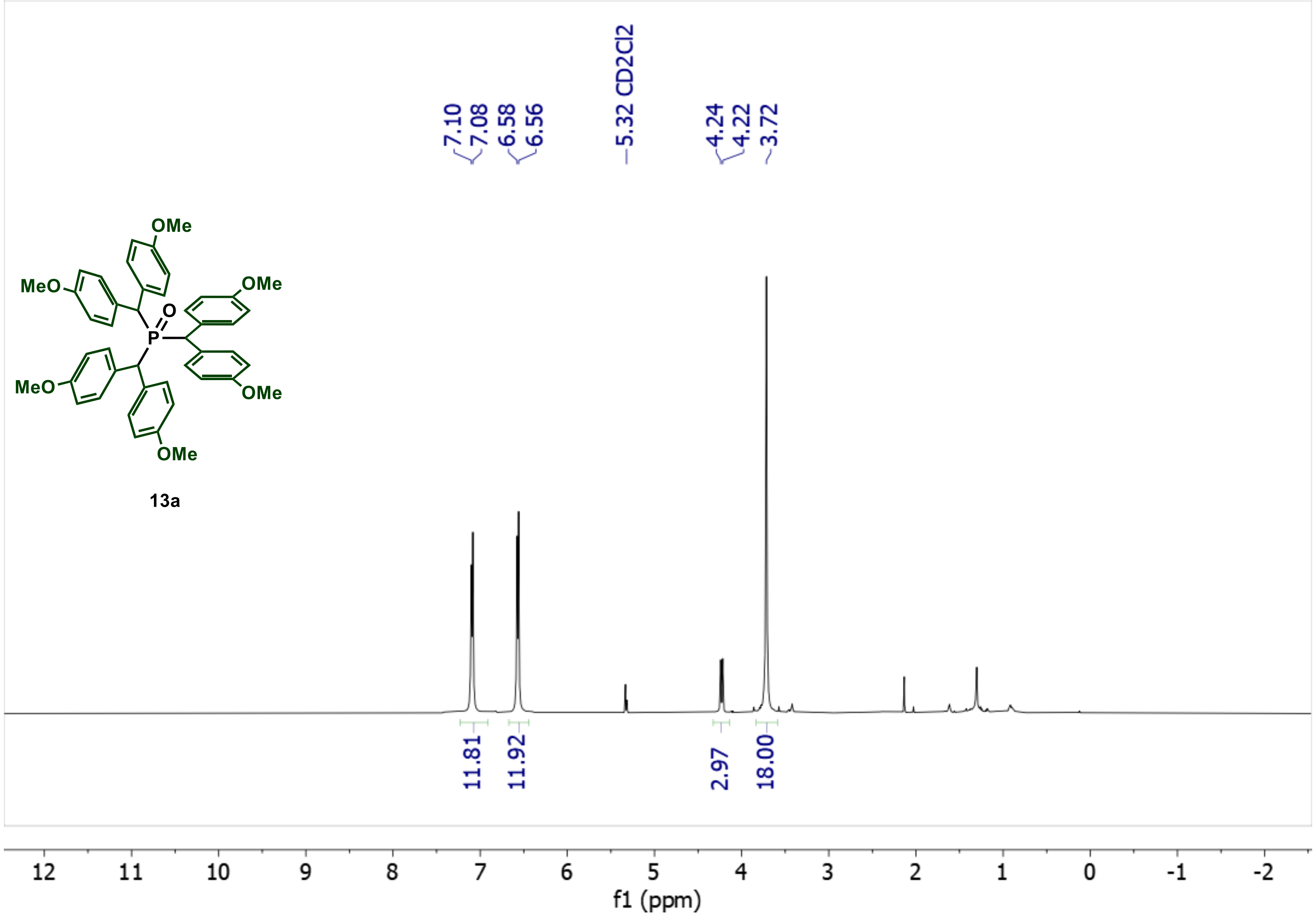

Figure 13. ^{1}H NMR (500 MHz, Methylene Chloride-d_2) of **13a**

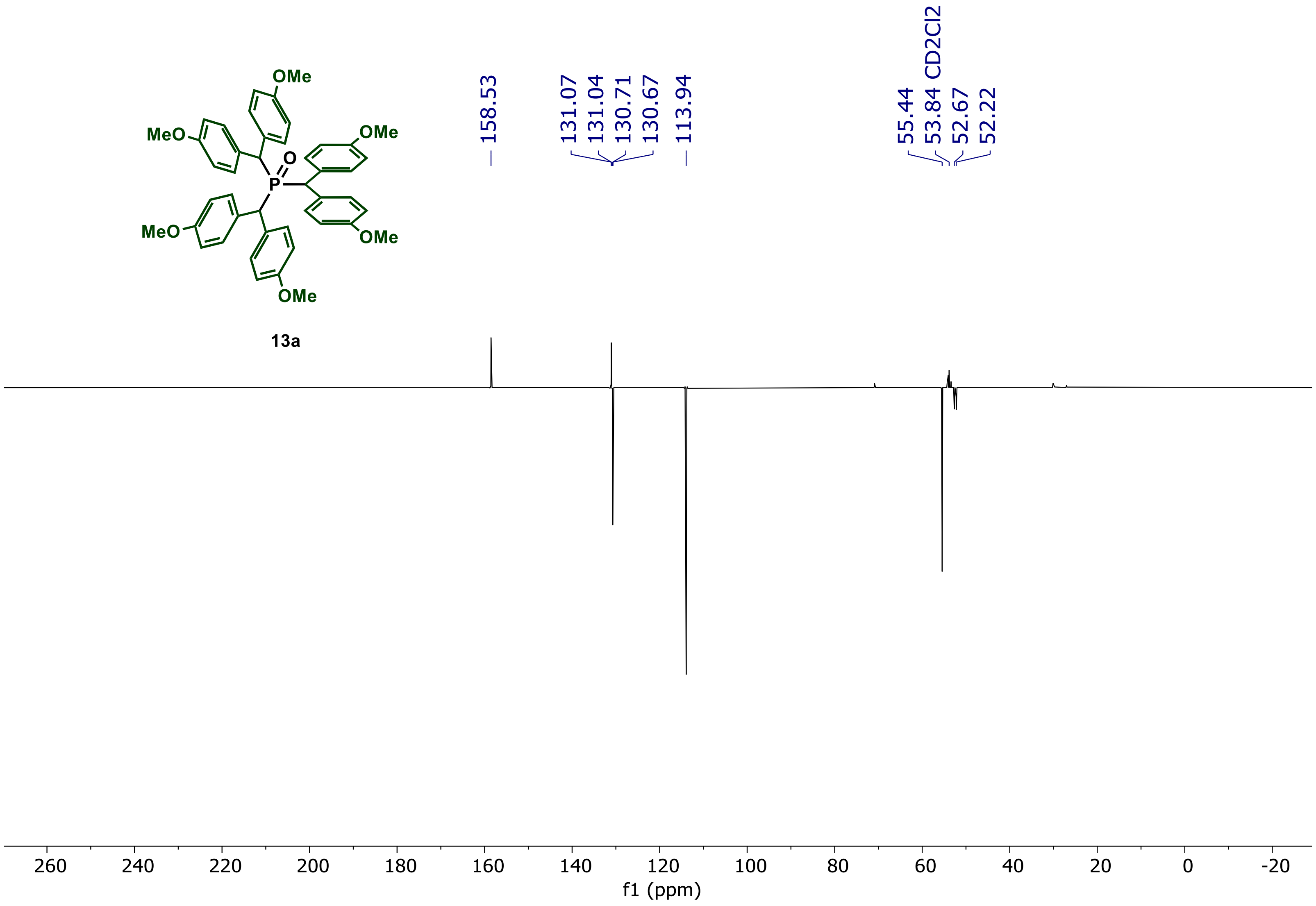

Figure 14. JMOD ^{13}C NMR (126 MHz, Methylene Chloride-d_2) of **13a**

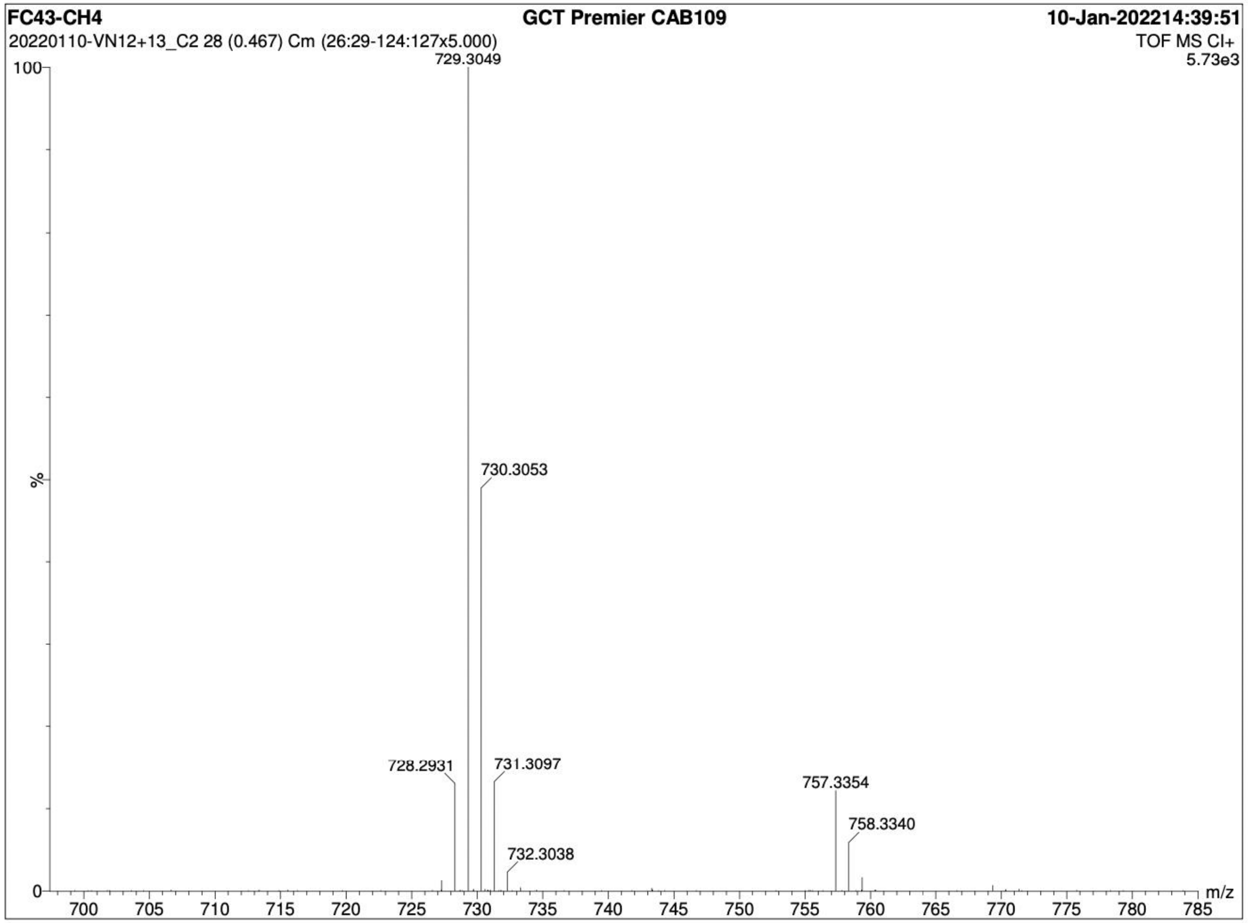

Figure 15. **HRMS** (DCI) for **13a**

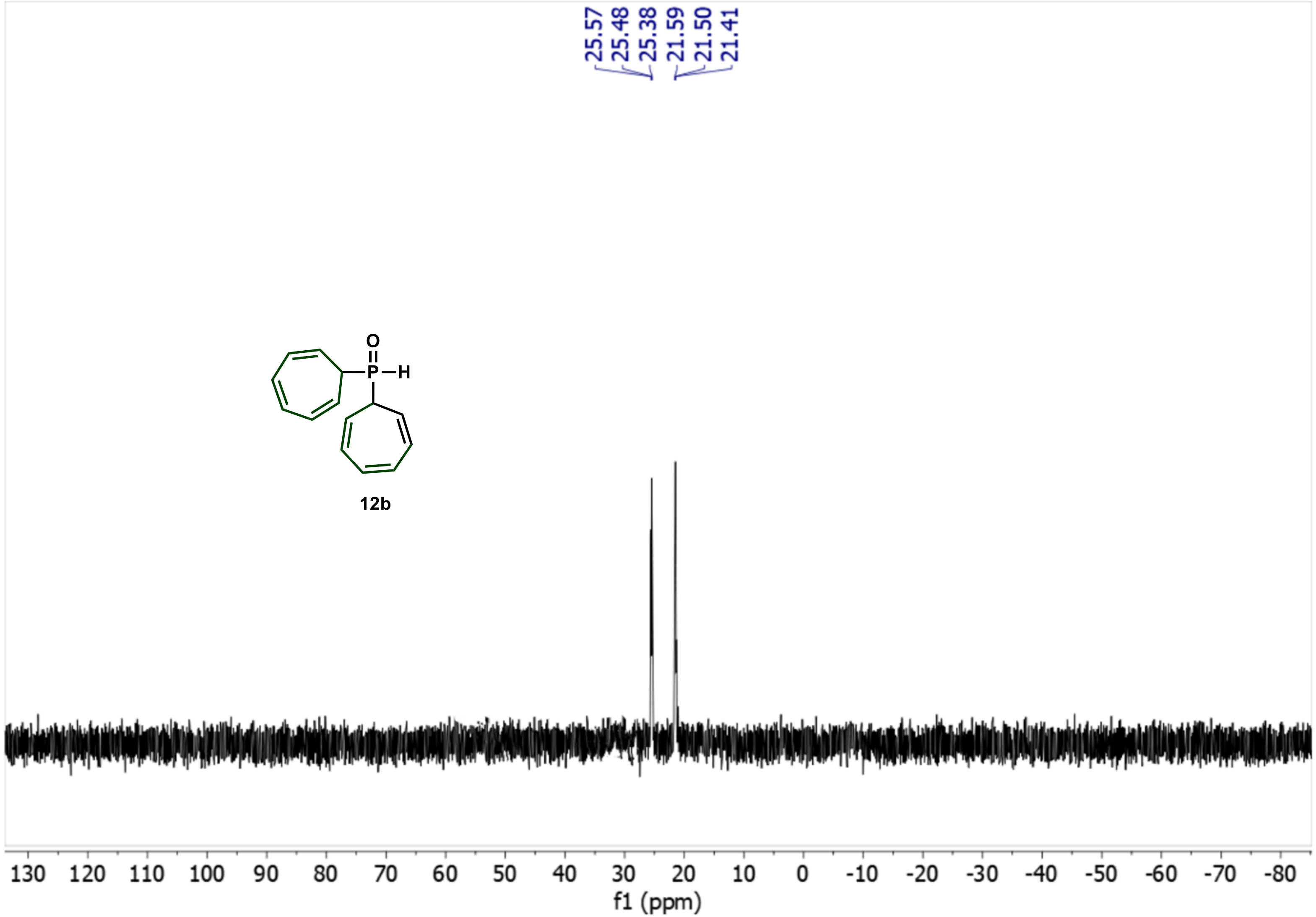

Figure 16. ^{31}P NMR (162 MHz, Methylene Chloride-d_2) of **12b**

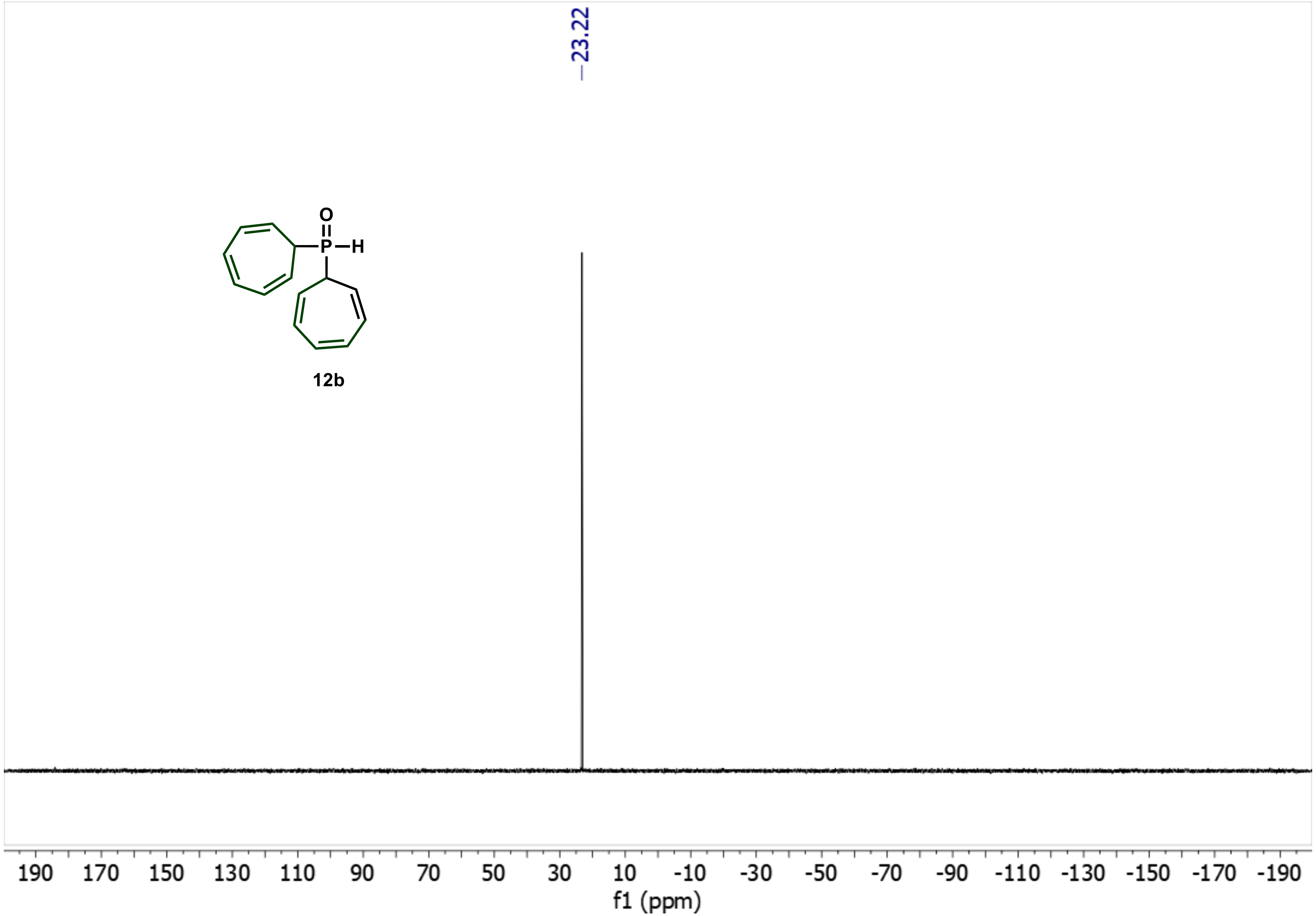

Figure 17. $^{31}P\{H\}$ NMR (162 MHz, Methylene Chloride-d_2) of **12b**

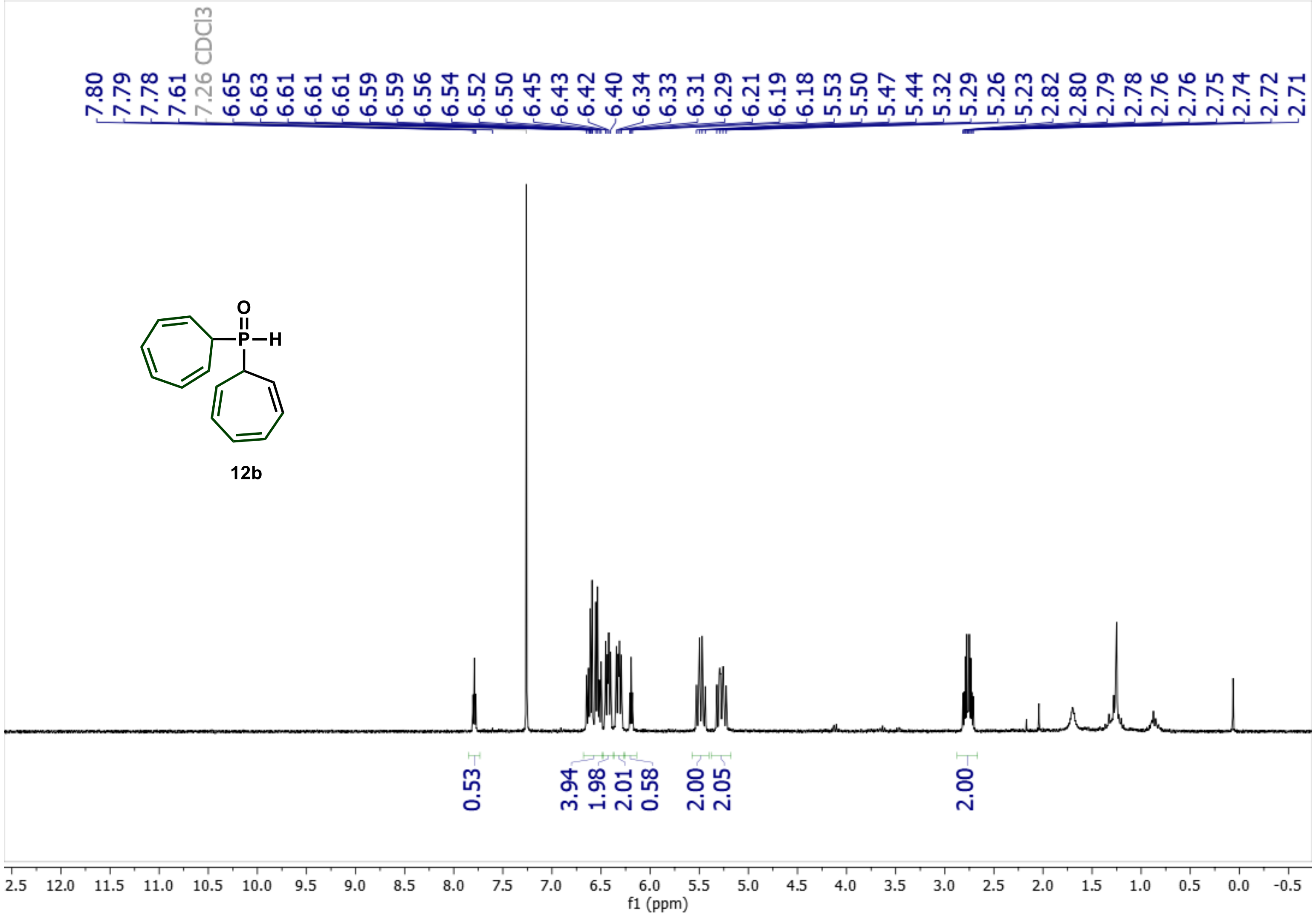

Figure 18. ^{1}H NMR (300 MHz, Chloroform-*d*) of **12b**

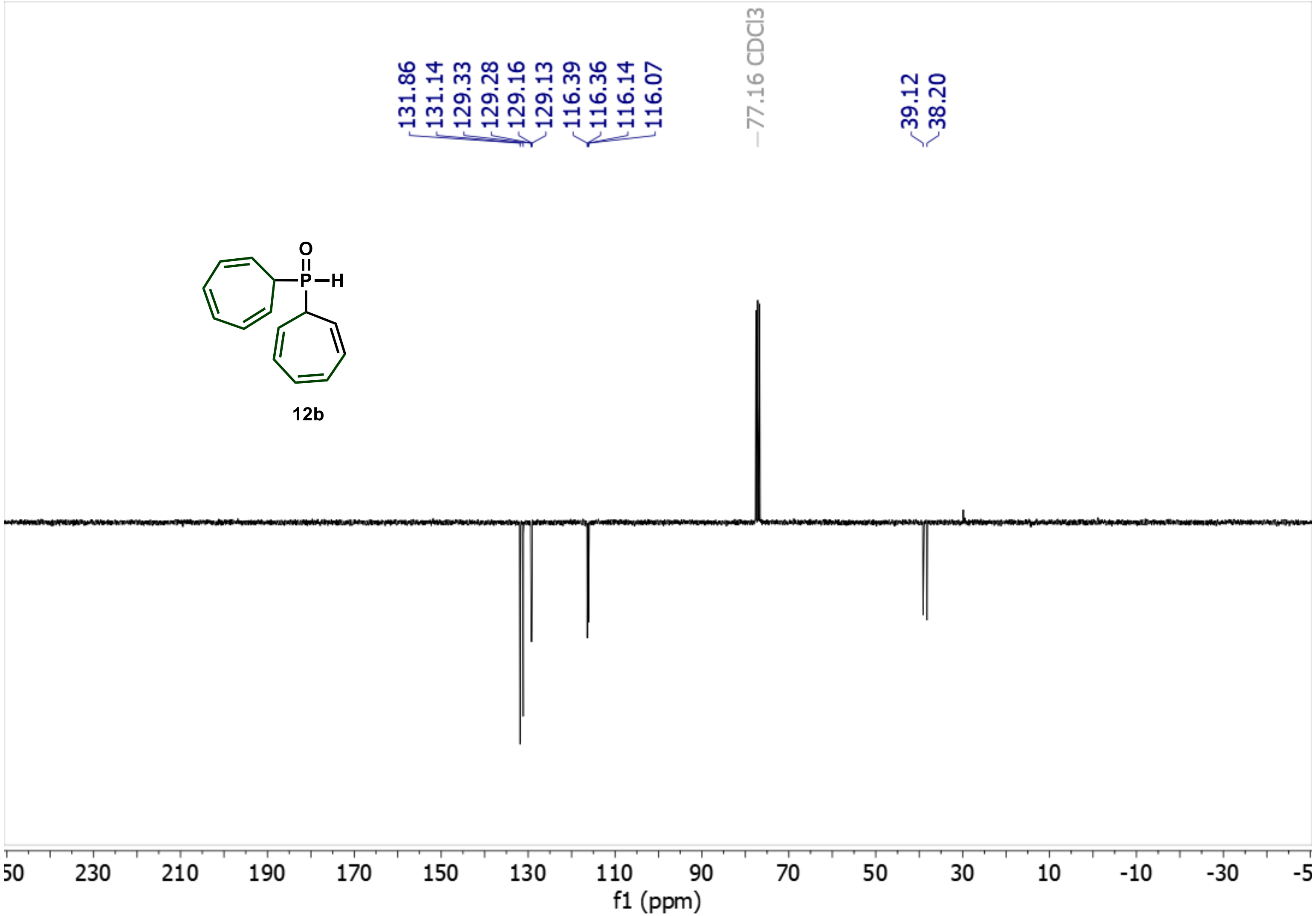

Figure 19. JMOD ^{13}C NMR (75 MHz, Chloroform-*d*) of **12b**

Single Mass Analysis

Tolerance = 5.0 PPM / DBE: min = -1.5, max = 80.0
Element prediction: Off

Monoisotopic Mass, Odd and Even Electron Ions
18 formula(e) evaluated with 1 results within limits (all results (up to 1000) for each mass)
Elements Used:
C: 0-100 H: 0-100 O: 0-5 P: 1-1

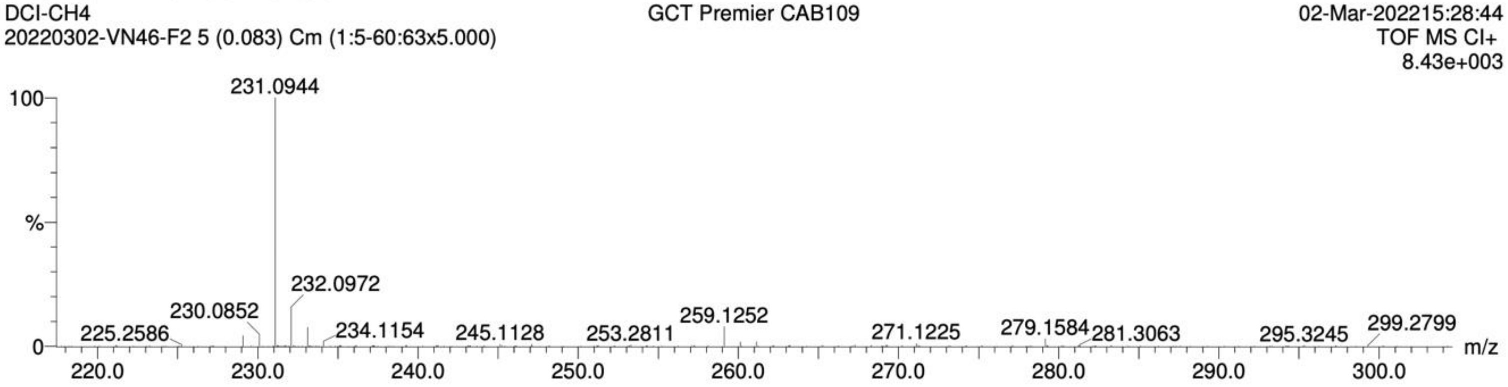

Minimum:				-1.5		
Maximum:		1.4	5.0	80.0		
Mass	Calc. Mass	mDa	PPM	DBE	i-FIT	Formula
231.0944	231.0939	0.5	2.2	7.5	211.4	C14 H16 O P

Figure 20. **HRMS** (DCI) for **12b**

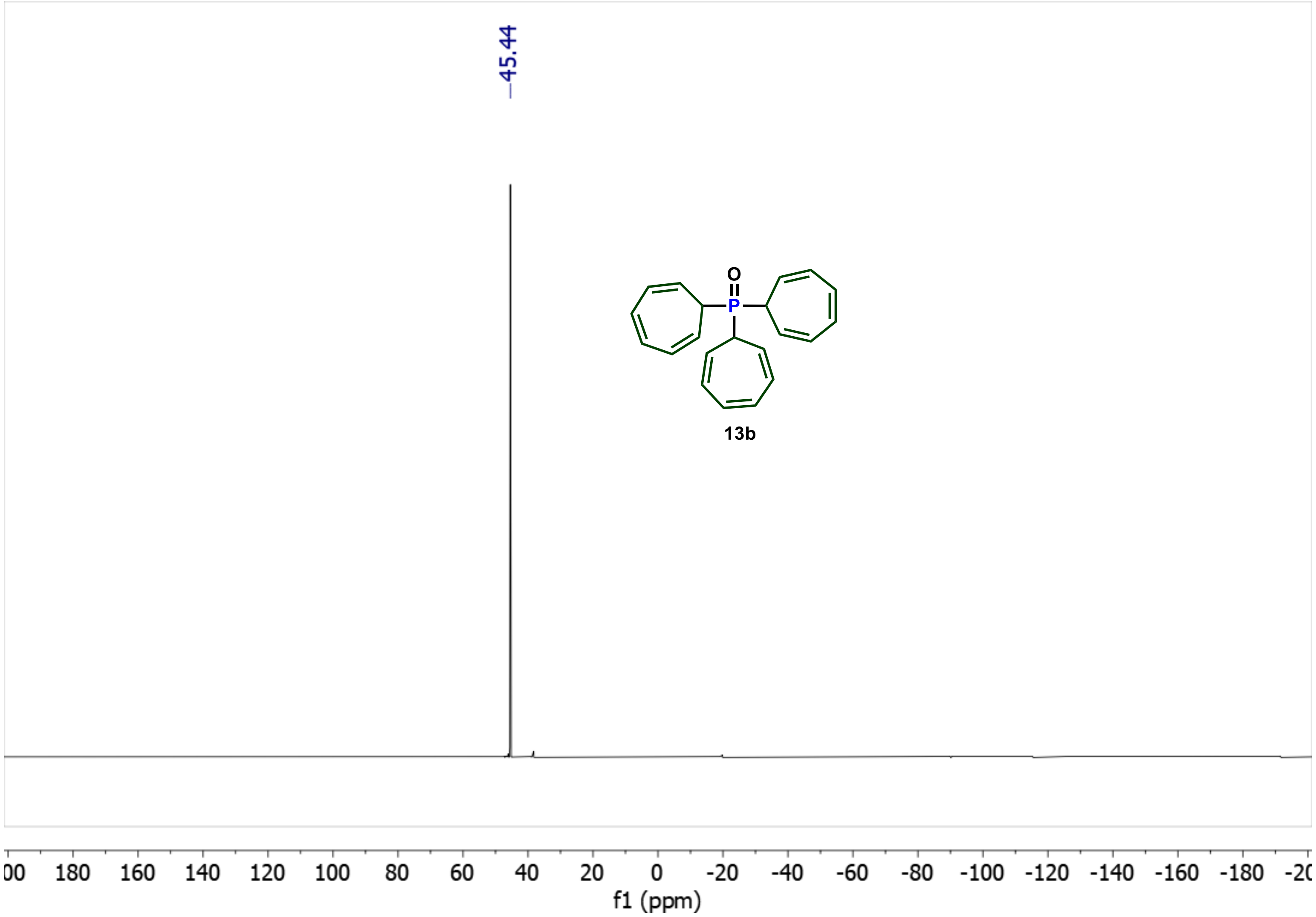

Figure 21. $^{31}P\{H\}$ NMR (162 MHz, Methylene Chloride-d_2) of **13b**

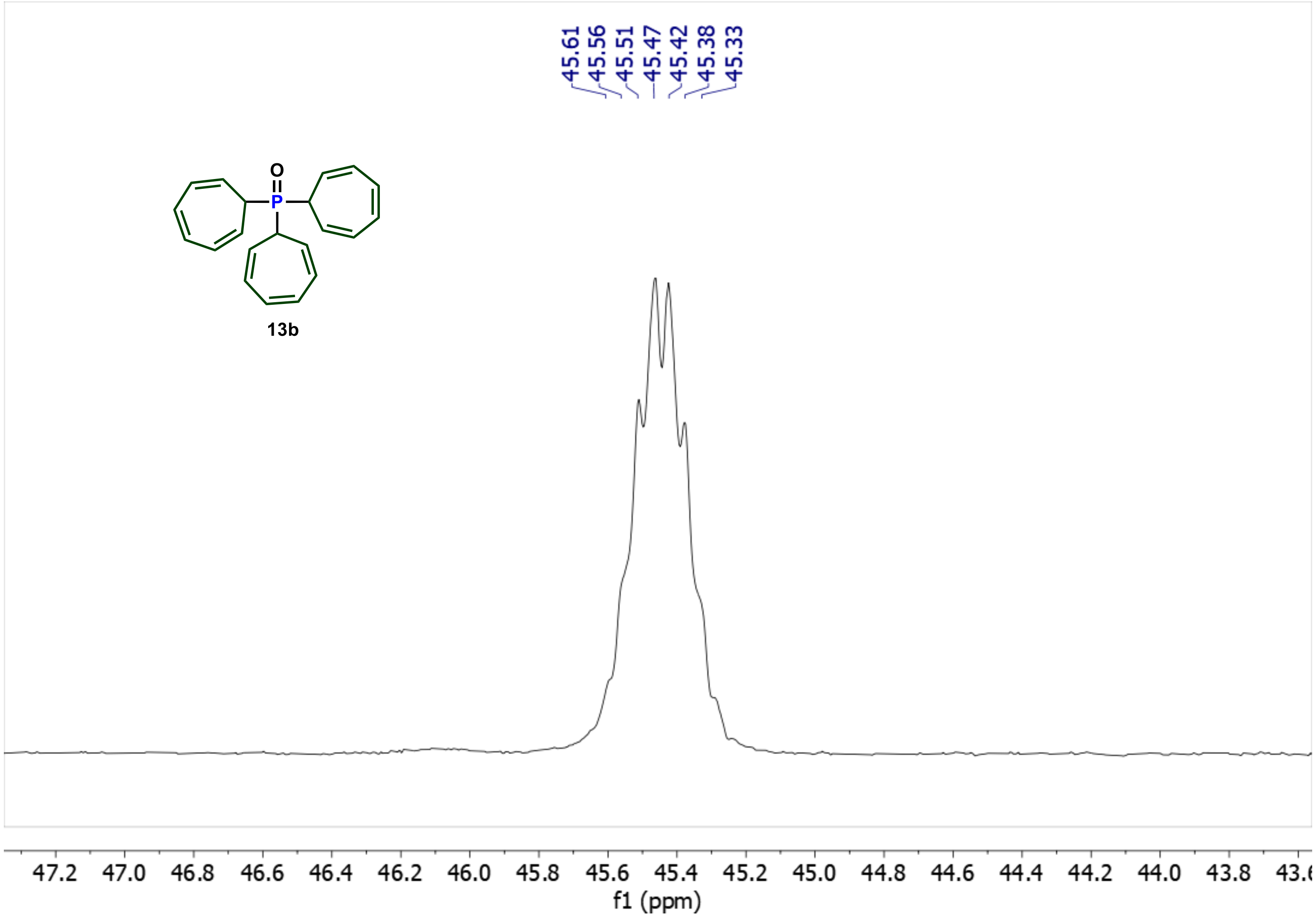

Figure 22. ^{31}P NMR (162 MHz, Methylene Chloride-d_2) of **13b**

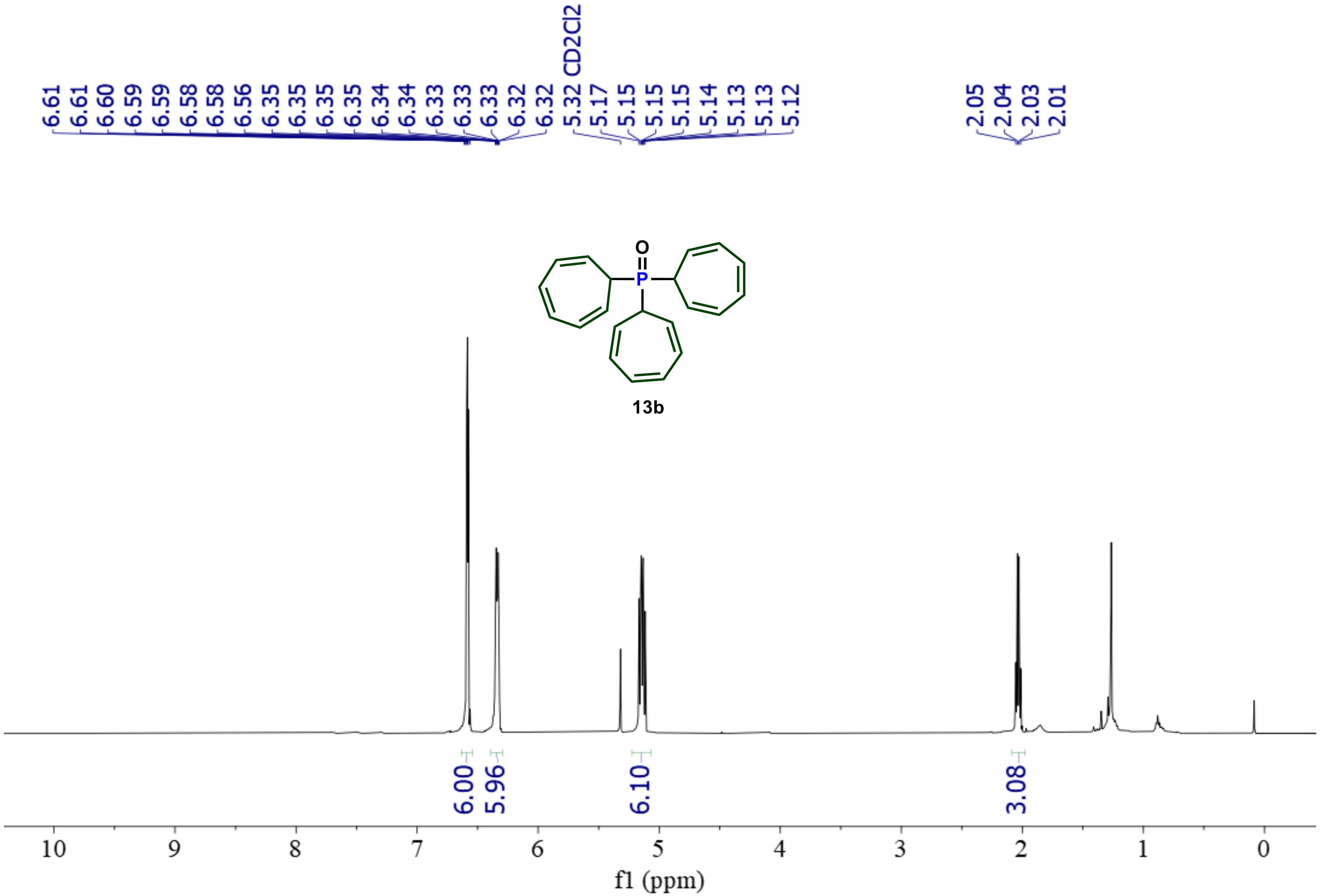

Figure 23. ^{1}H NMR (500 MHz, Methylene Chloride-d_2) of **13b**

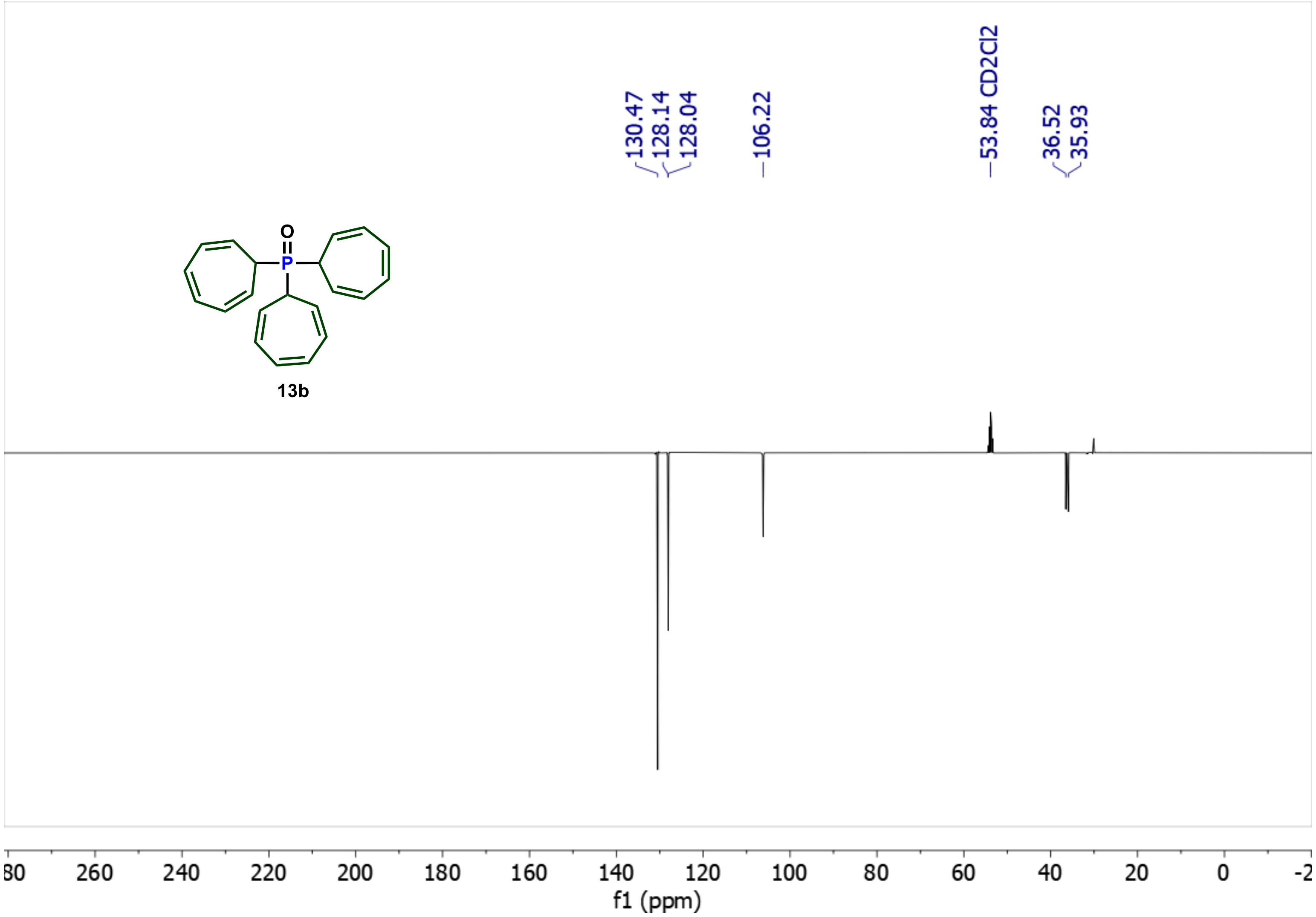

Figure 24. JMOD ^{13}C NMR (126 MHz, Methylene Chloride-d_2) of **13b**

Single Mass Analysis

Tolerance = 5.0 PPM / DBE: min = -1.5, max = 80.0
Element prediction: Off

Monoisotopic Mass, Odd and Even Electron Ions
23 formula(e) evaluated with 1 results within limits (all results (up to 1000) for each mass)
Elements Used:
C: 0-100 H: 0-100 O: 0-5 P: 1-1

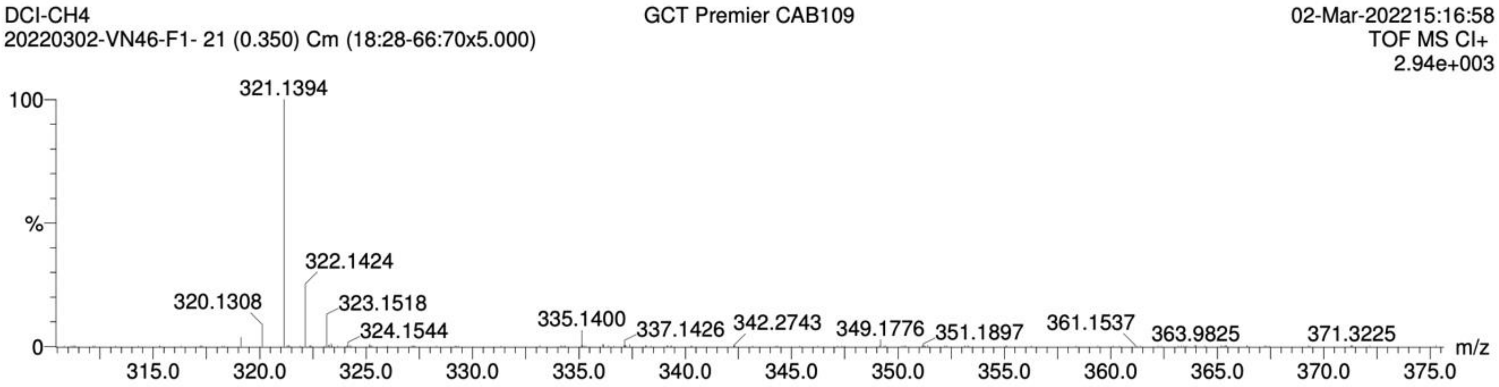

Minimum:				-1.5		
Maximum:		1.4	5.0	80.0		
Mass	Calc. Mass	mDa	PPM	DBE	i-FIT	Formula
321.1394	321.1408	-1.4	-4.4	11.5	115.4	C21 H22 O P

Figure 25. HRMS (DCI) for **13b**

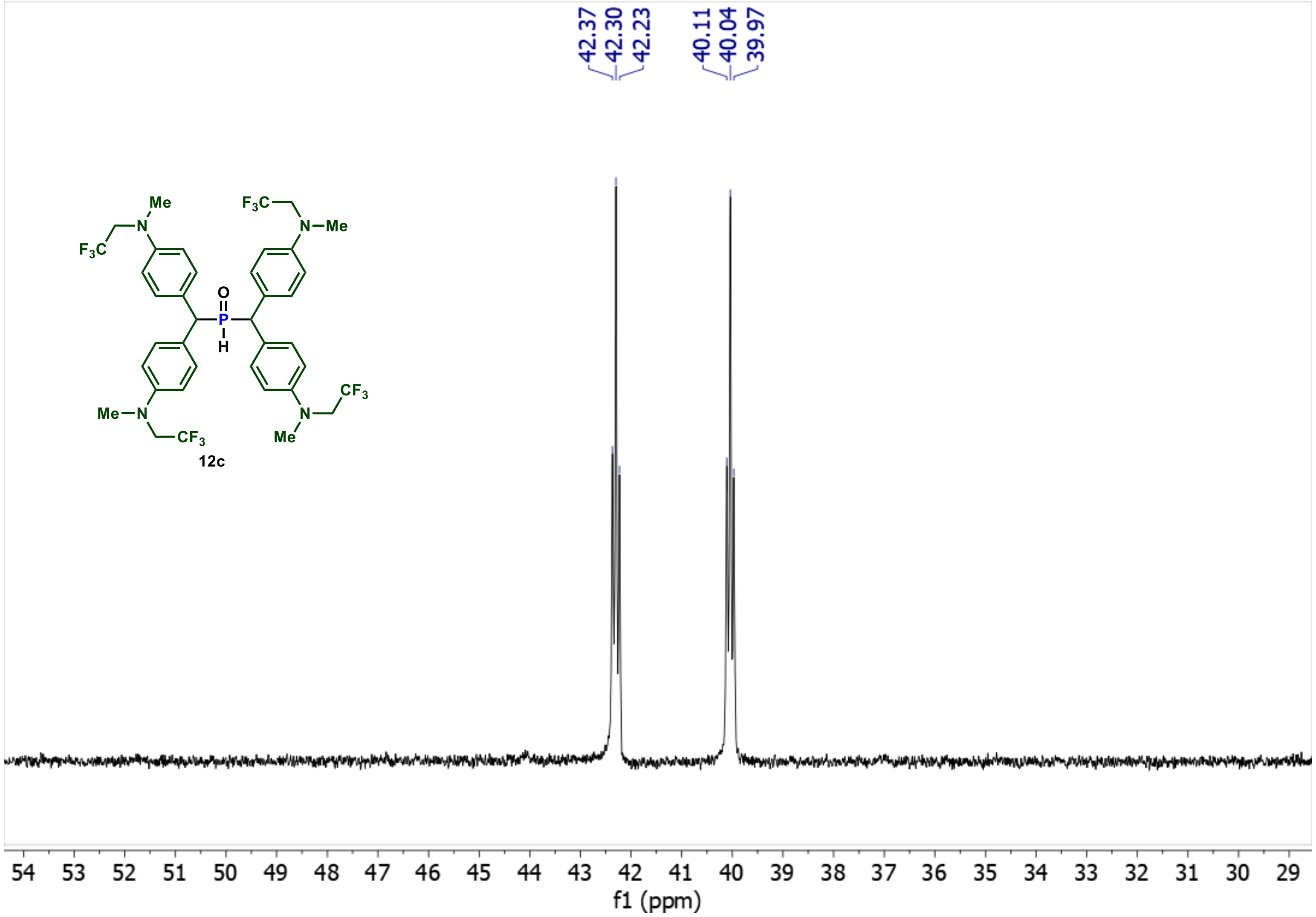

Figure 26. ^{31}P NMR (162 MHz, Methylene Chloride-d_2) of **12c**

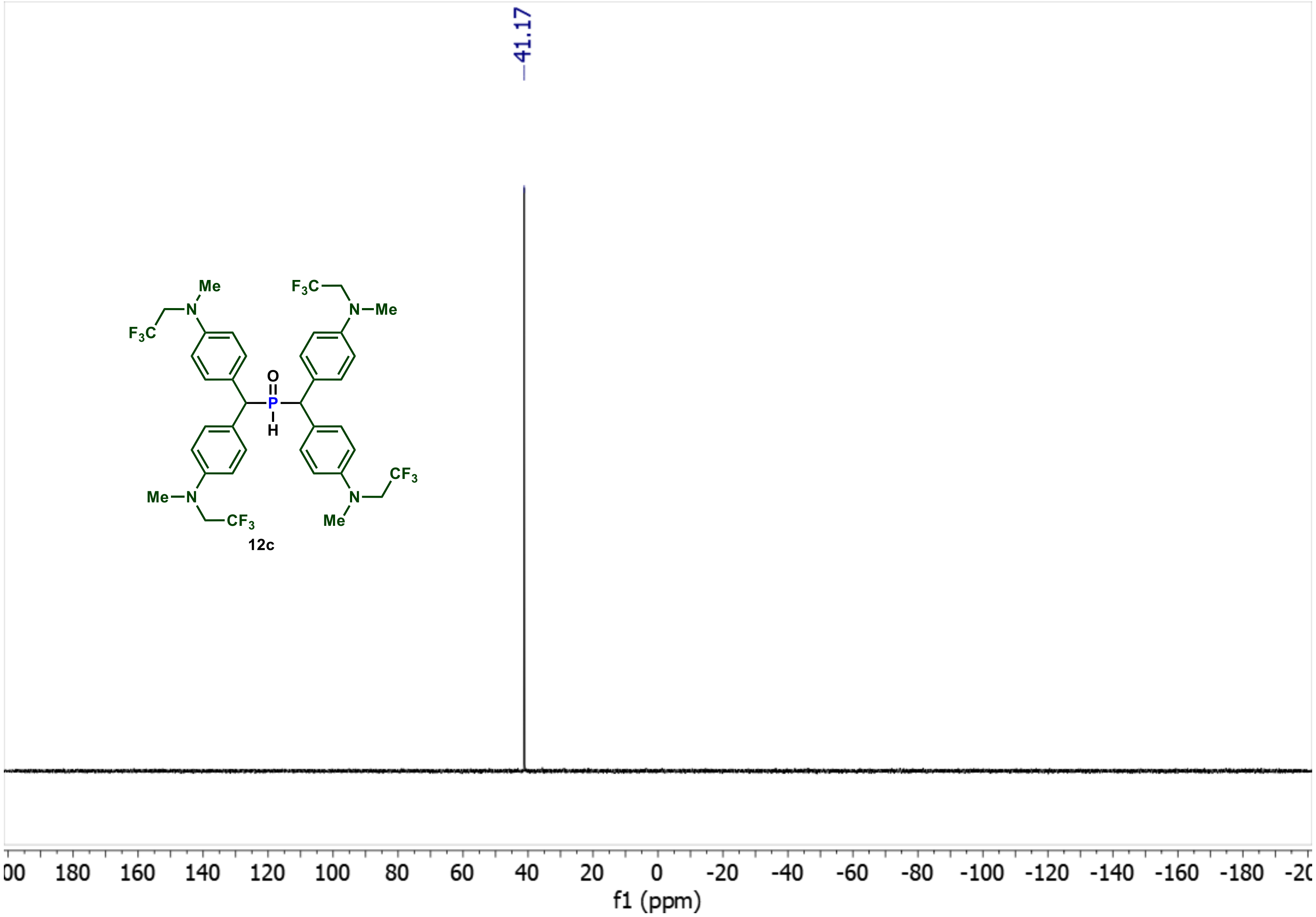

Figure 27. $^{31}P\{H\}$ NMR (162 MHz, Methylene Chloride-d_2) of **12c**

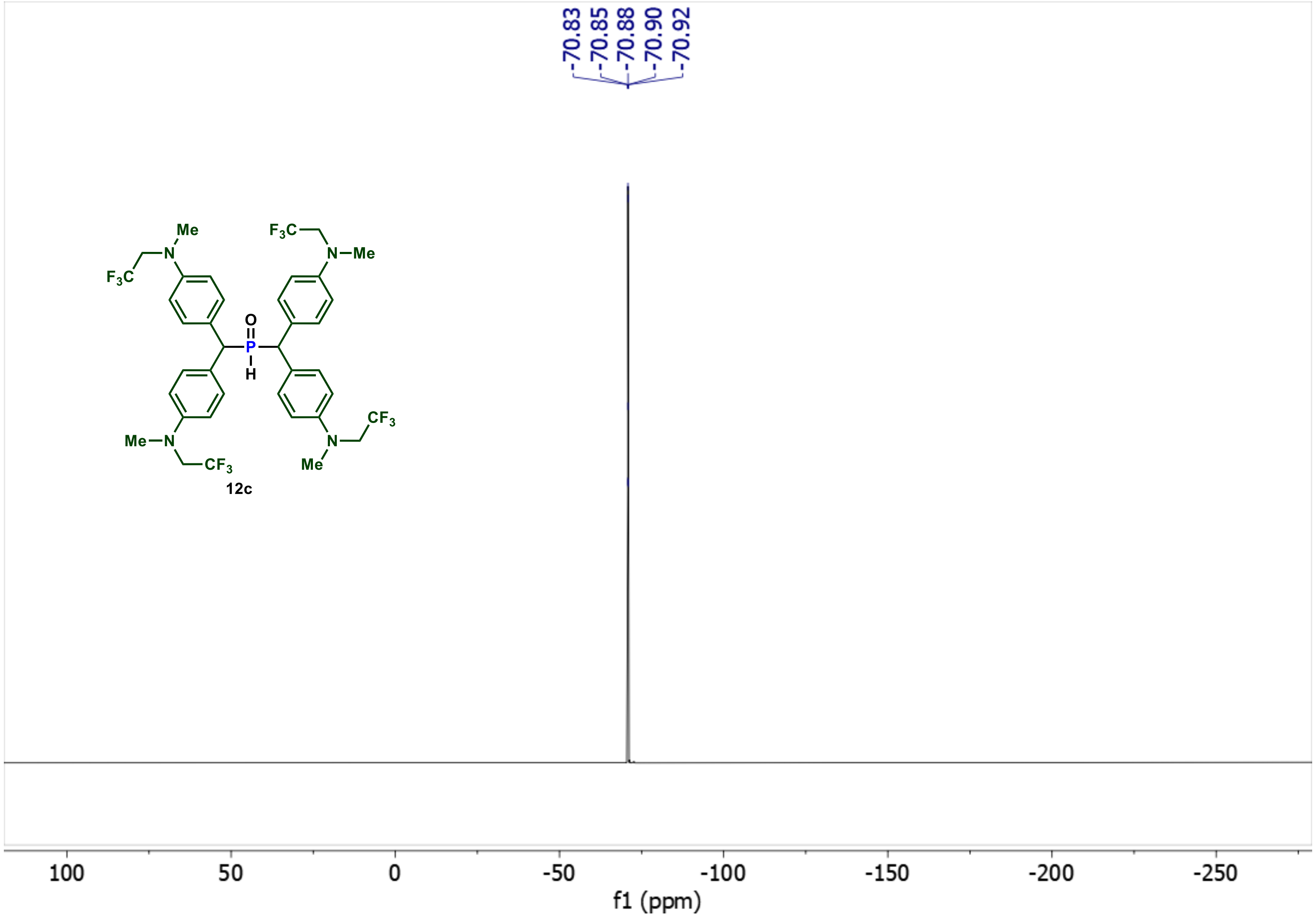

Figure 28. ^{19}F NMR (471 MHz, Methylene Chloride-d_2) of **12c**

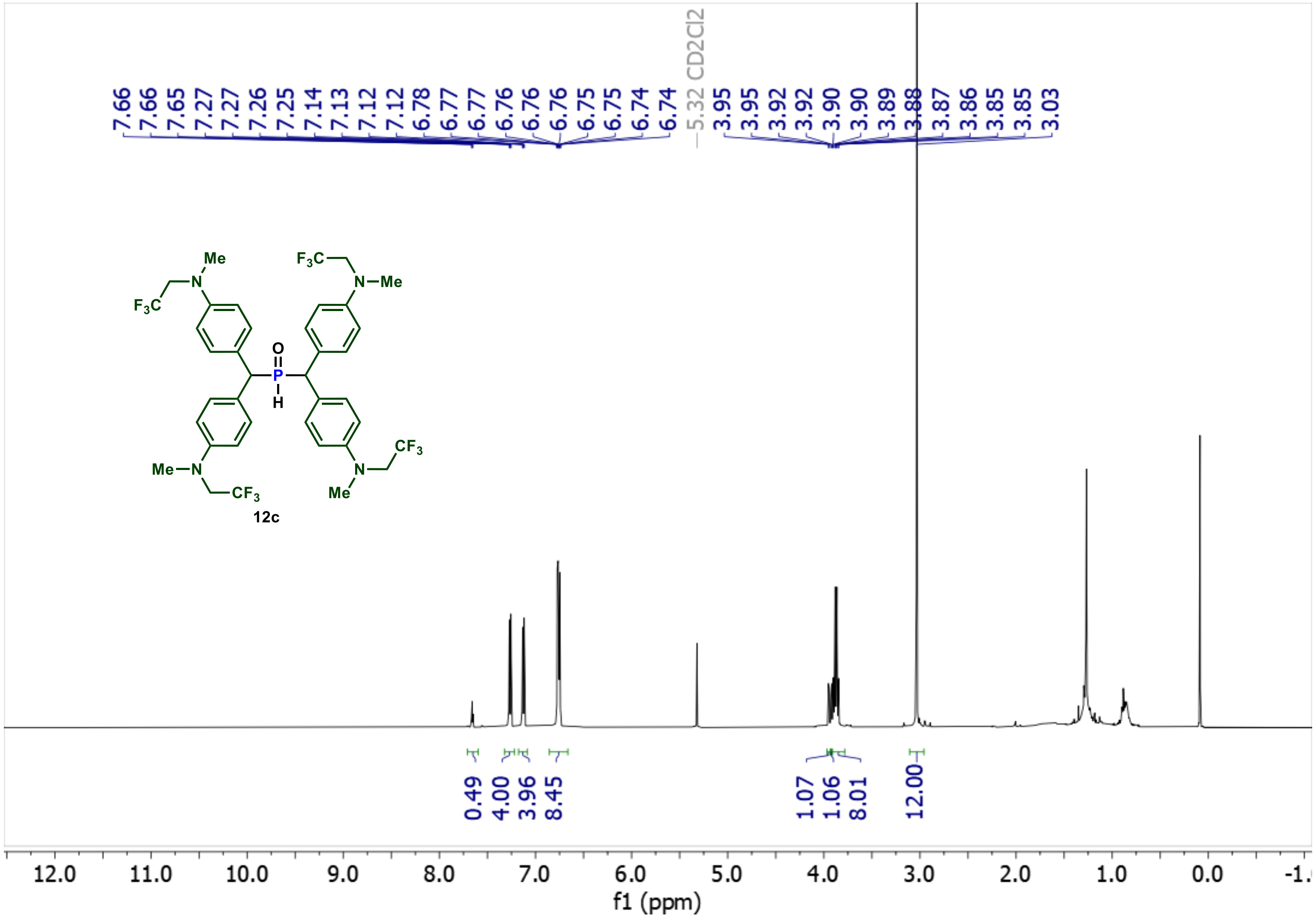

Figure 29. ^{1}H NMR (500 MHz, Methylene Chloride-d_2) of **12c**

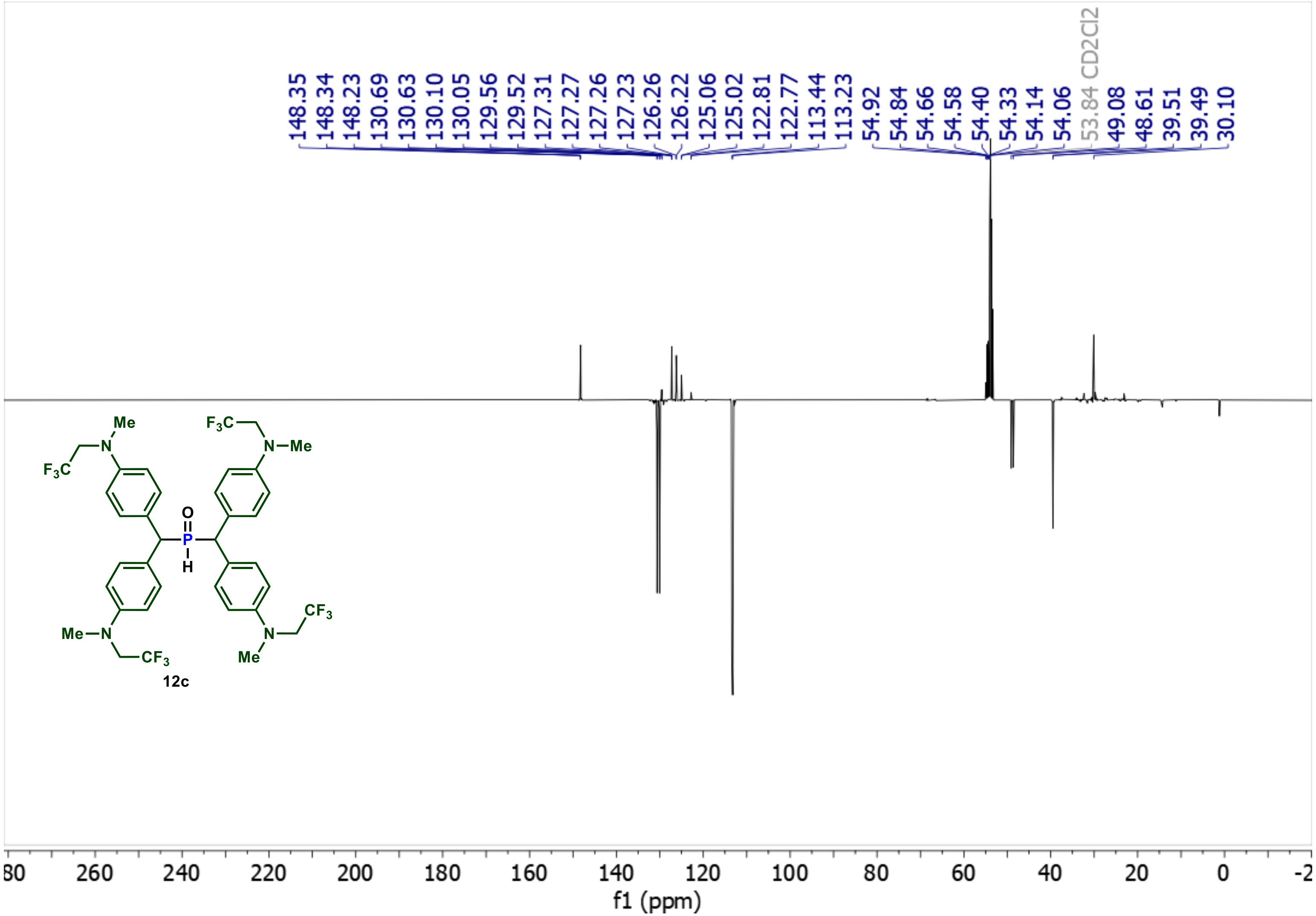

Figure 30. JMOD ^{13}C NMR (126 MHz, Methylene Chloride-d_2) of **12c**

Single Mass Analysis
Tolerance = 5.0 PPM / DBE: min = -1.5, max = 80.0
Element prediction: Off

Monoisotopic Mass, Odd and Even Electron Ions
240 formula(e) evaluated with 2 results within limits (all results (up to 1000) for each mass)
Elements Used:
C: 0-100 H: 0-100 N: 0-5 O: 0-5 F: 12-12 P: 1-1

DCI/CH4 GCT Premier CAB109 02-Jun-202315:27:10
20230602-VN02-180 F3 30 (0.500) Cm (24:30-65:71x5.000) TOF MS CI+ 2.29e+004

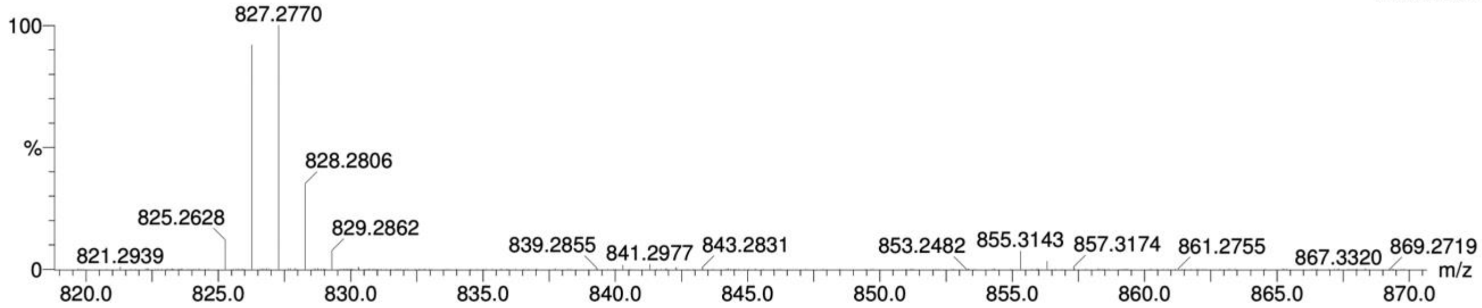

Minimum:				-1.5		
Maximum:		1.4	5.0	80.0		
Mass	Calc. Mass	mDa	PPM	DBE	i-FIT	Formula
826.2702	826.2683	1.9	2.3	15.5	4959.5	C40 H41 N O2 F12 P
	826.2670	3.2	3.9	16.0	5241.5	C38 H39 N4 O F12 P

Figure 31. HRMS (DCI) for **12c**

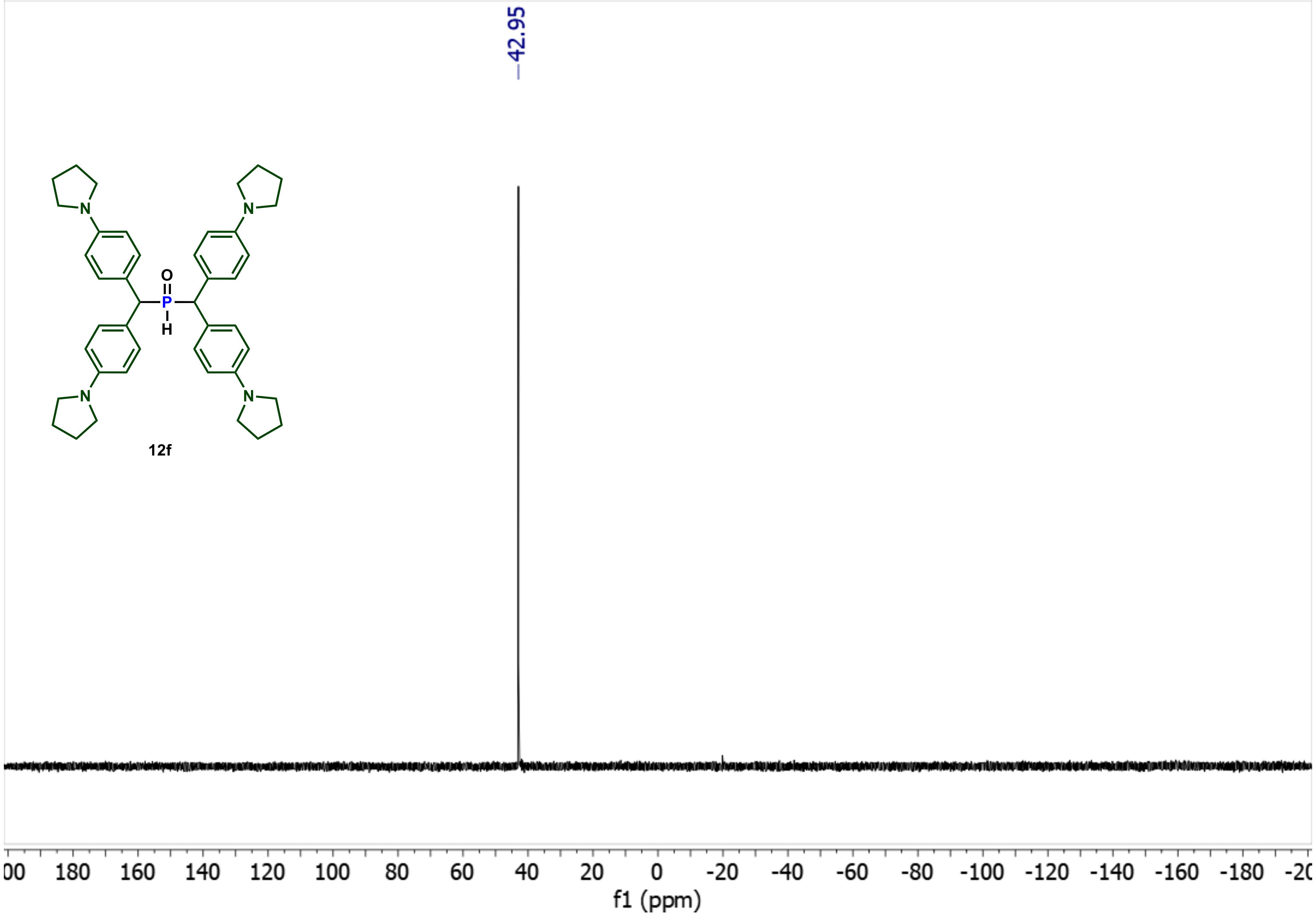

Figure 32. $^{31}P\{H\}$ NMR (202 MHz, Methylene Chloride-d_2) of **12f**

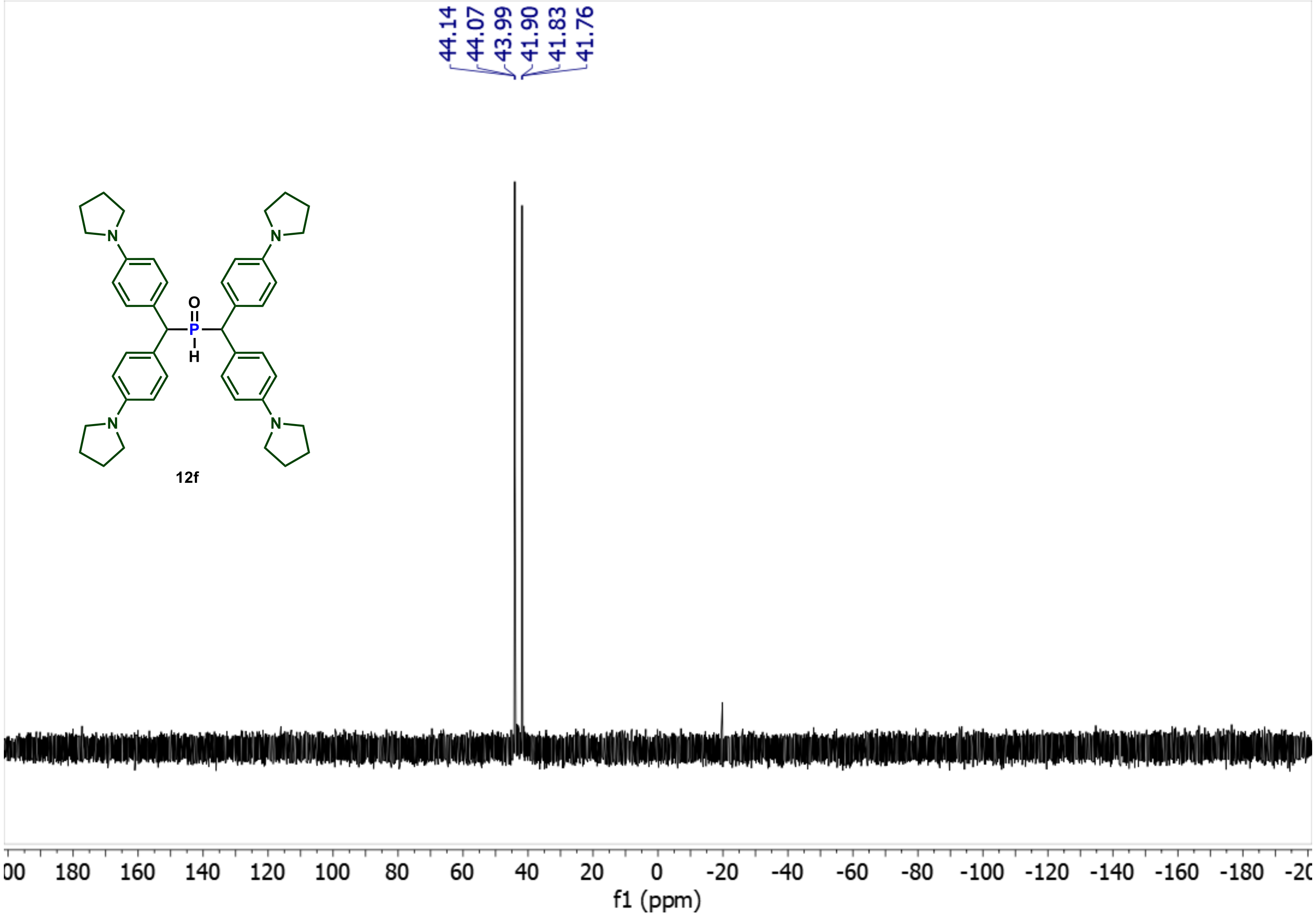

Figure 33. ^{31}P NMR (202 MHz, Methylene Chloride-d_2) of **12f**

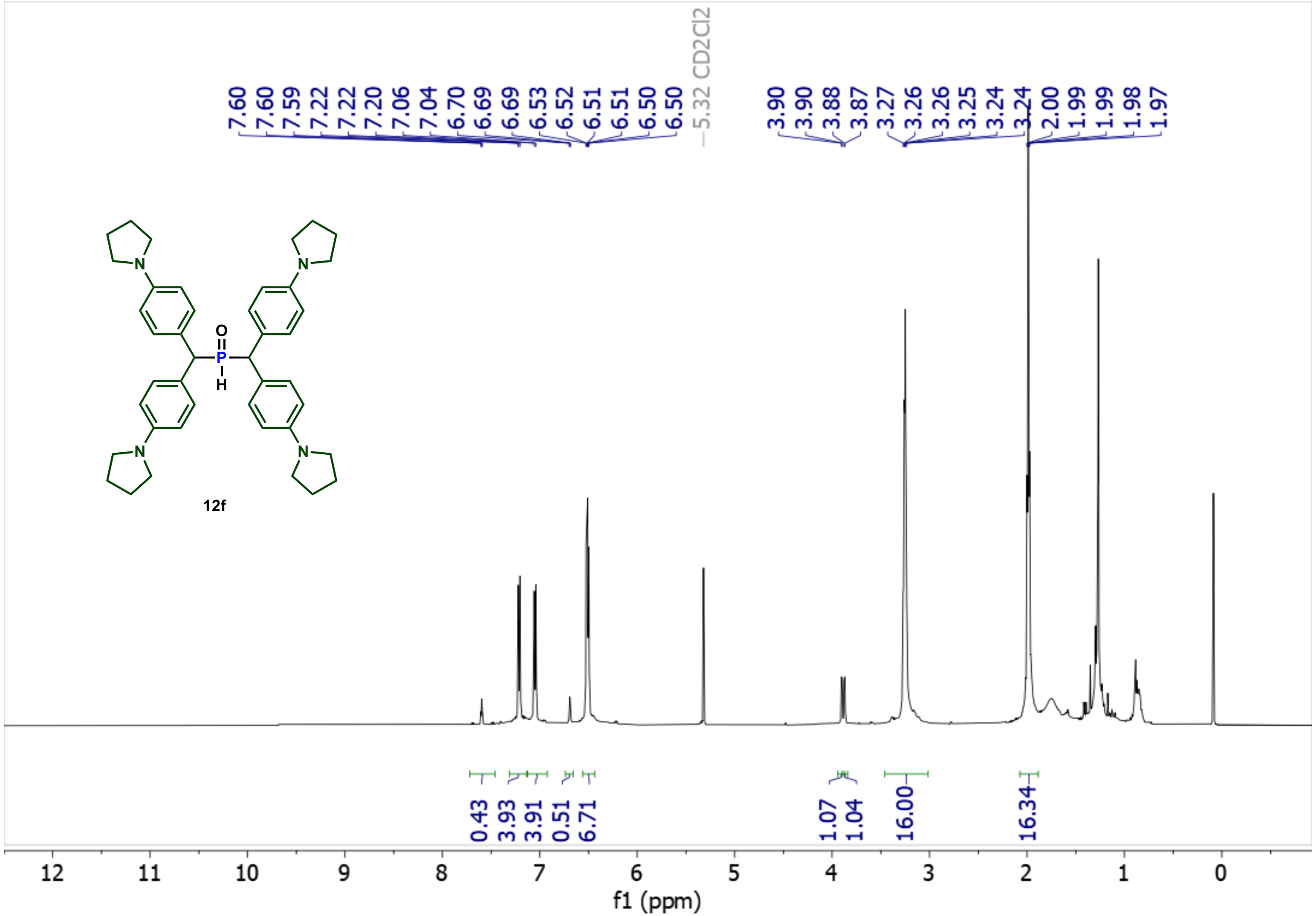

Figure 34. ^{1}H NMR (500 MHz, Methylene Chloride-d_2) of **12f**

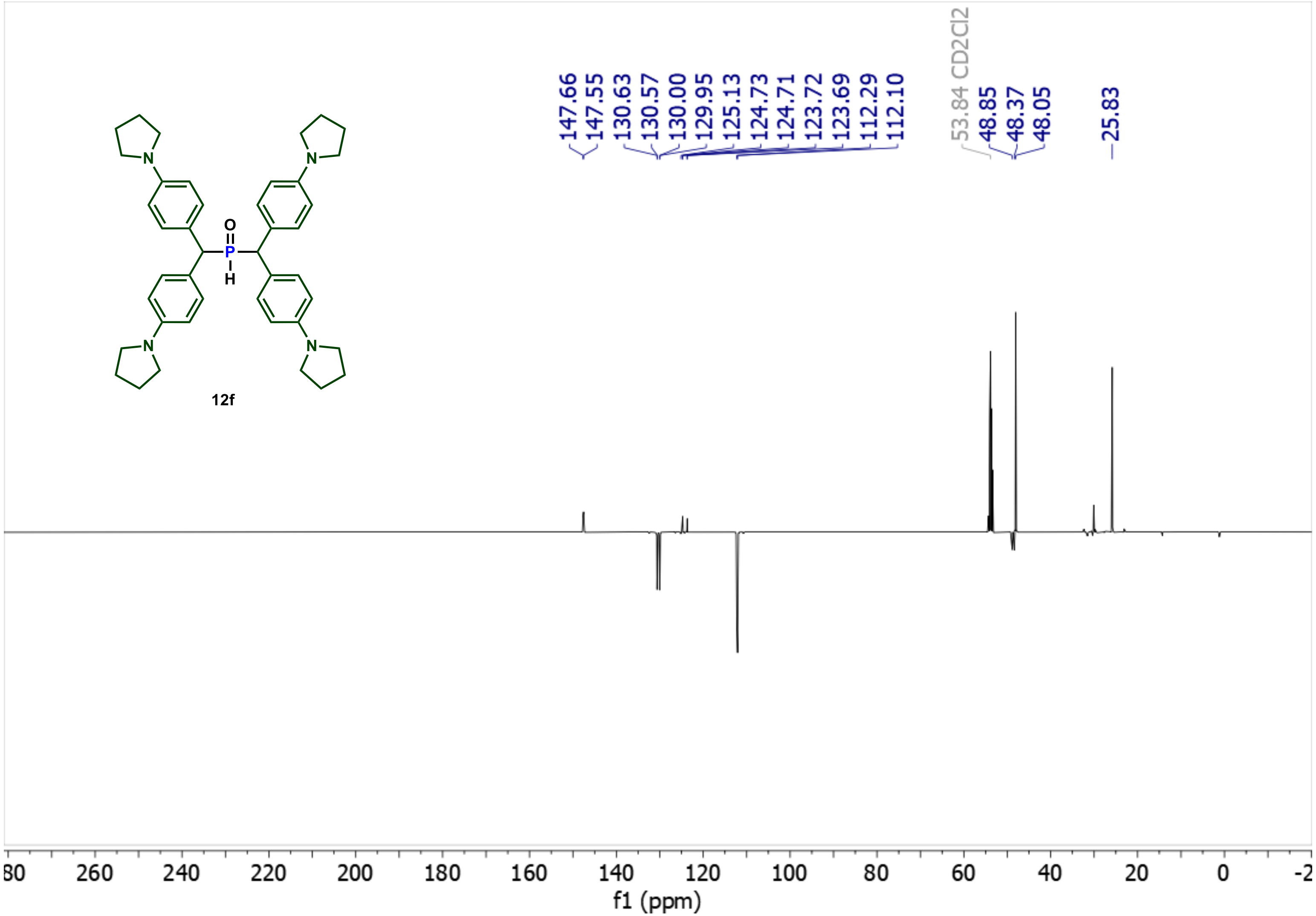

Figure 35. JMOD ^{13}C NMR (126 MHz, Methylene Chloride-d_2) of **12f**

Single Mass Analysis
Tolerance = 5.0 PPM / DBE: min = -1.5, max = 80.0
Element prediction: Off

Monoisotopic Mass, Odd and Even Electron Ions
282 formula(e) evaluated with 3 results within limits (all results (up to 1000) for each mass)
Elements Used:
C: 0-100 H: 0-100 N: 0-5 O: 0-5 P: 1-1

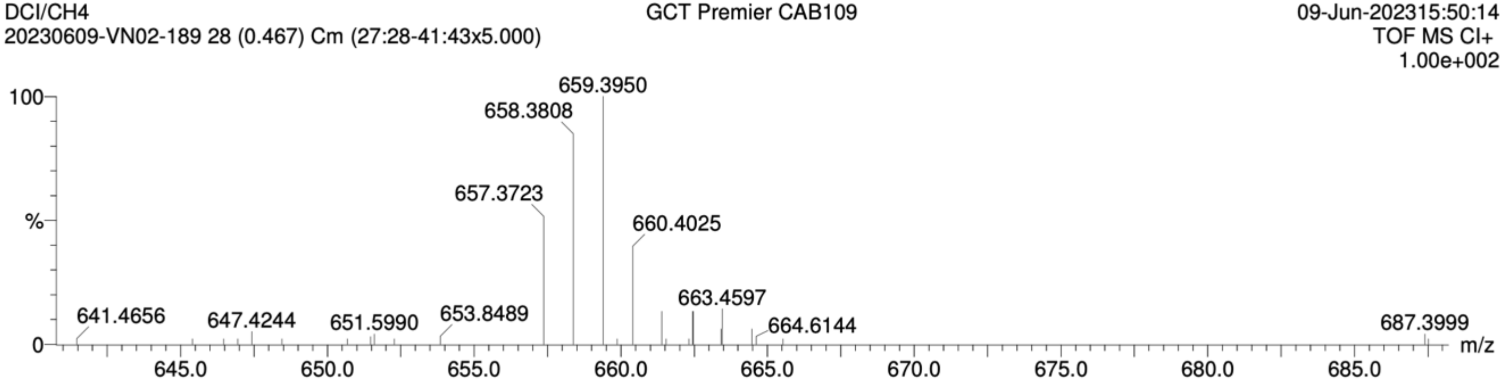

Minimum:				-1.5		
Maximum:		1.4	5.0	80.0		
Mass	Calc. Mass	mDa	PPM	DBE	i-FIT	Formula
658.3808	658.3814	-0.6	-0.9	19.5	22.1	C44 H53 N O2 P
	658.3801	0.7	1.1	20.0	23.3	C42 H51 N4 O P
	658.3787	2.1	3.2	15.0	24.8	C41 H55 O5 P

Figure 36. HRMS (DCI) for **12f**

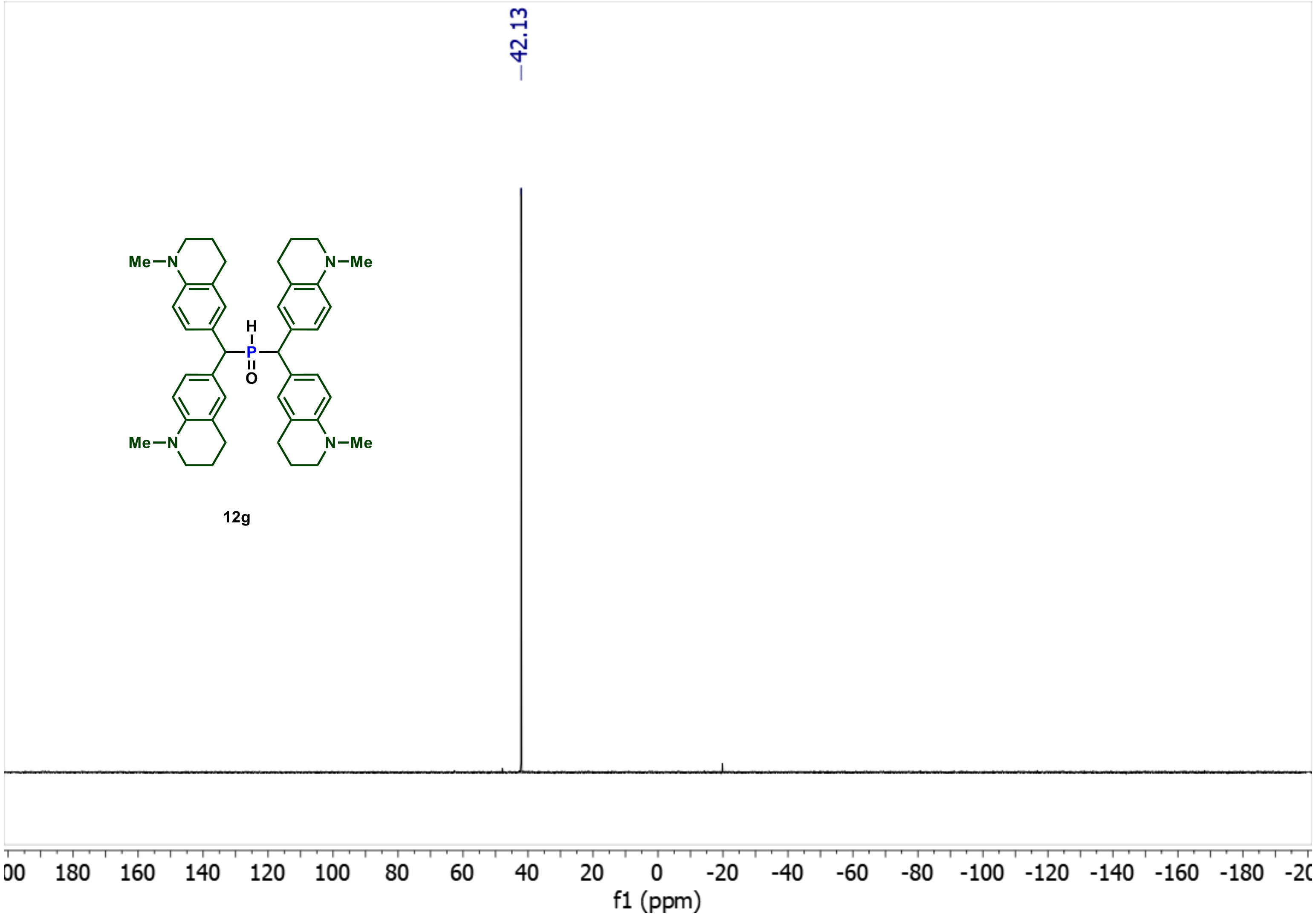

Figure 37. $^{31}P\{H\}$ NMR (162 MHz, Methylene Chloride-d_2) of **12g**

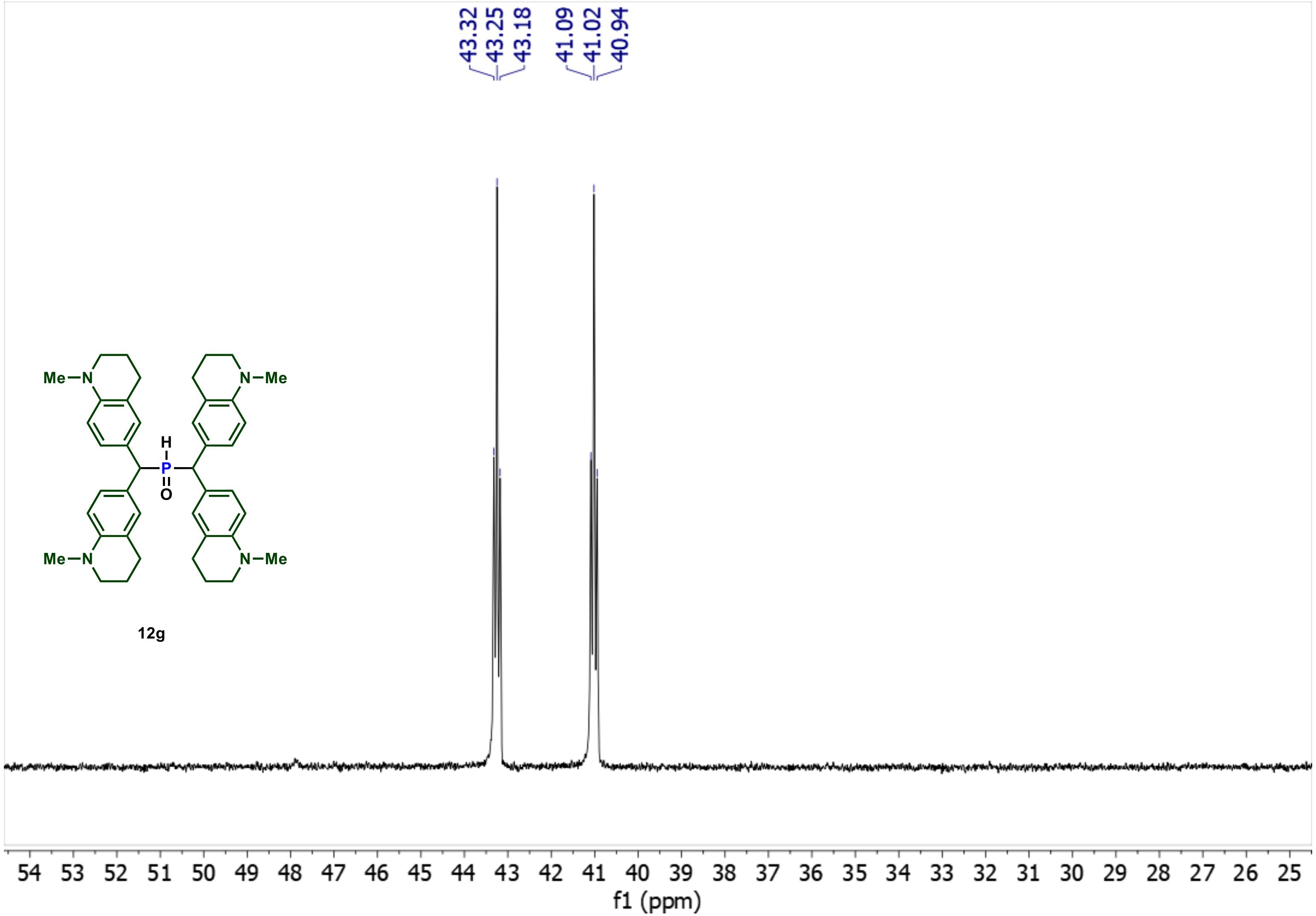

12g

Figure 38. ^{31}P NMR (162 MHz, Methylene Chloride-d_2) of **12g**

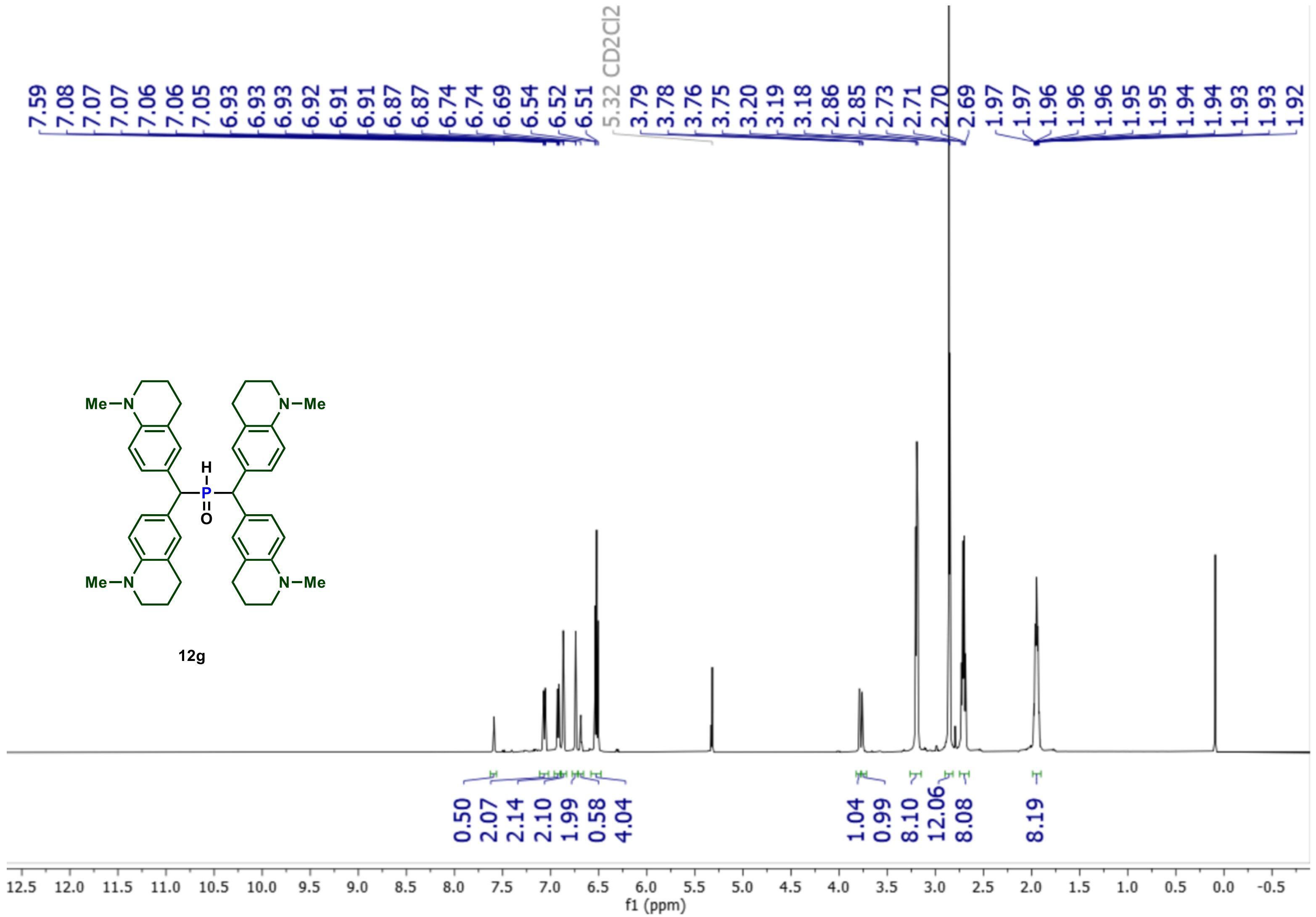

Figure 39. ^{1}H NMR (500 MHz, Methylene Chloride-d_2) of **12g**

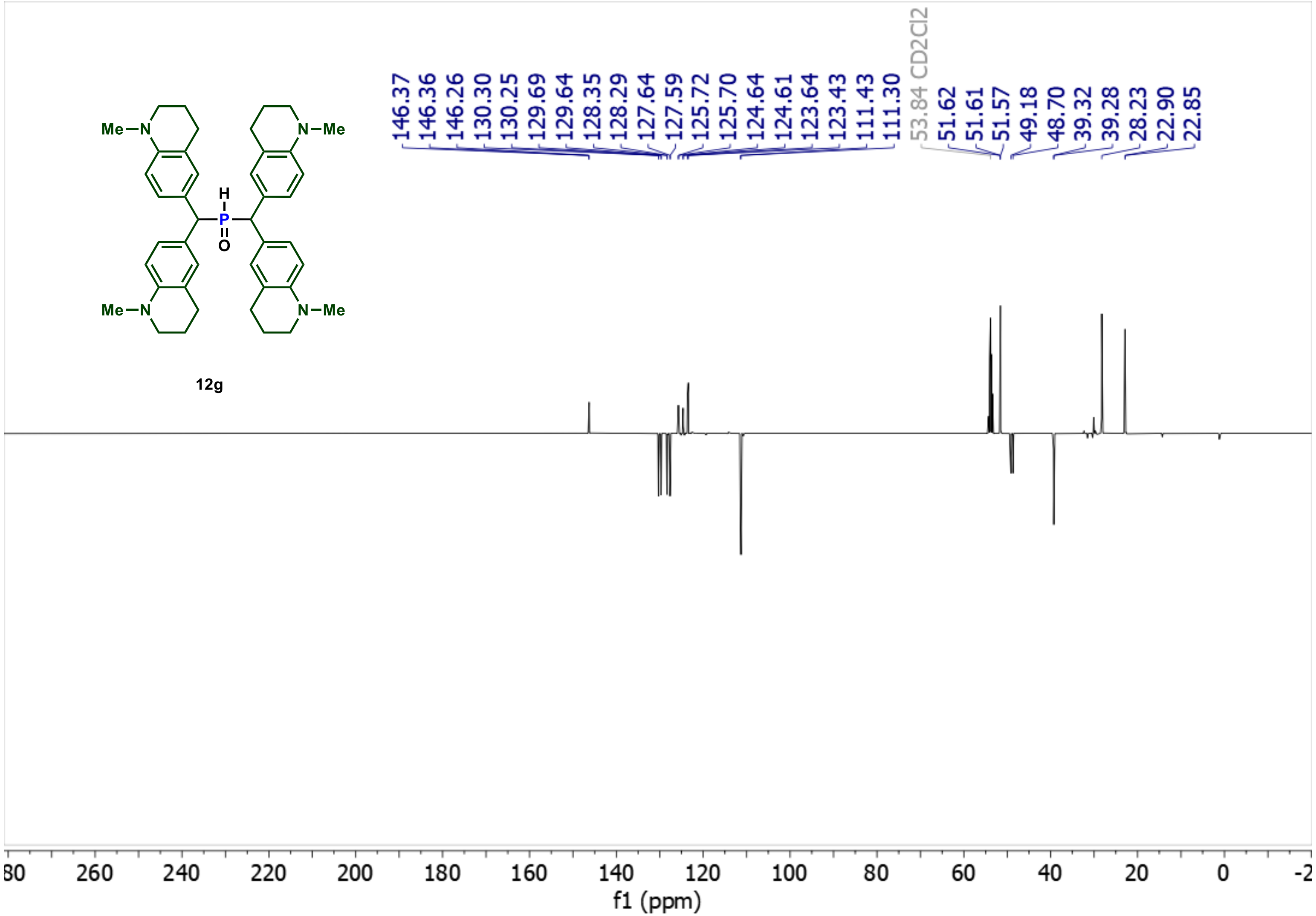

Figure 40. JMOD ^{13}C NMR (126 MHz, Methylene Chloride-d_2) of **12g**

Single Mass Analysis
Tolerance = 5.0 PPM / DBE: min = -1.5, max = 80.0
Element prediction: Off

Monoisotopic Mass, Odd and Even Electron Ions
282 formula(e) evaluated with 3 results within limits (all results (up to 1000) for each mass)
Elements Used:
C: 0-100 H: 0-100 N: 0-5 O: 0-5 P: 1-1

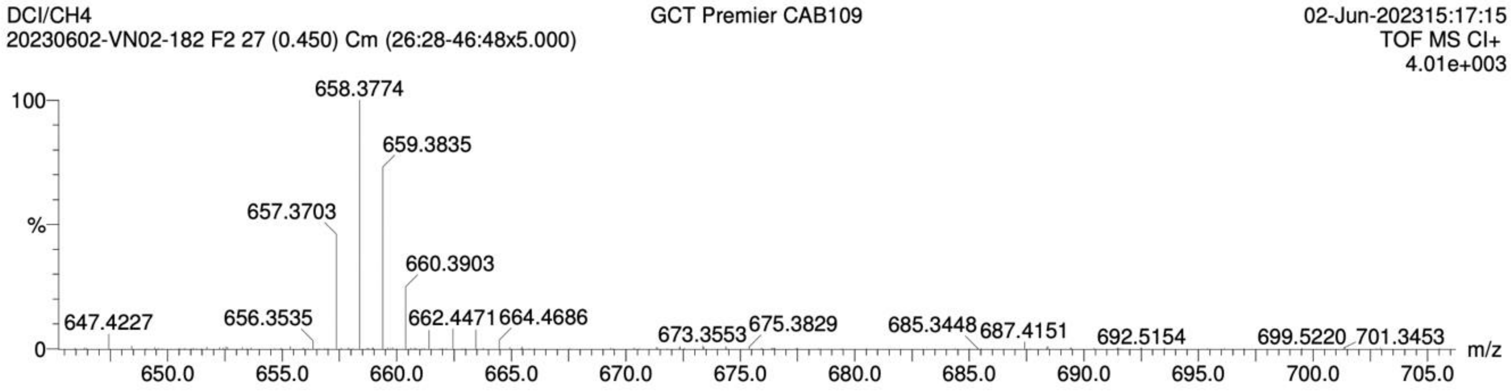

Minimum:				-1.5		
Maximum:		1.4	5.0	80.0		
Mass	Calc. Mass	mDa	PPM	DBE	i-FIT	Formula
658.3774	658.3801	-2.7	-4.1	20.0	219.7	C42 H51 N4 O P
	658.3787	-1.3	-2.0	15.0	252.3	C41 H55 O5 P
	658.3774	0.0	0.0	15.5	283.4	C39 H53 N3 O4 P

Figure 41. HRMS (DCI) for **12g**

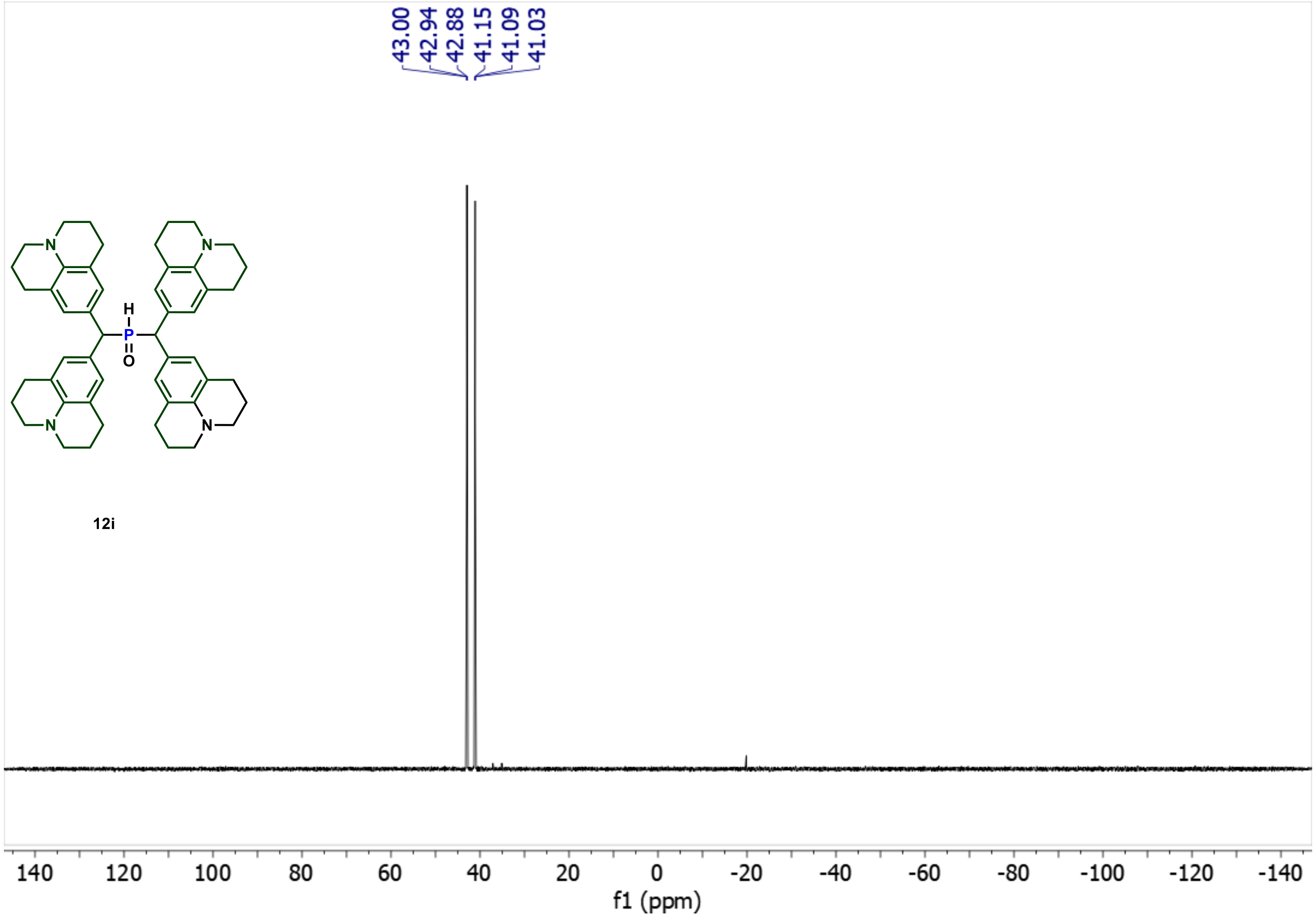

Figure 42. ^{31}P NMR (243 MHz, Methylene Chloride-d_2) of **12i**

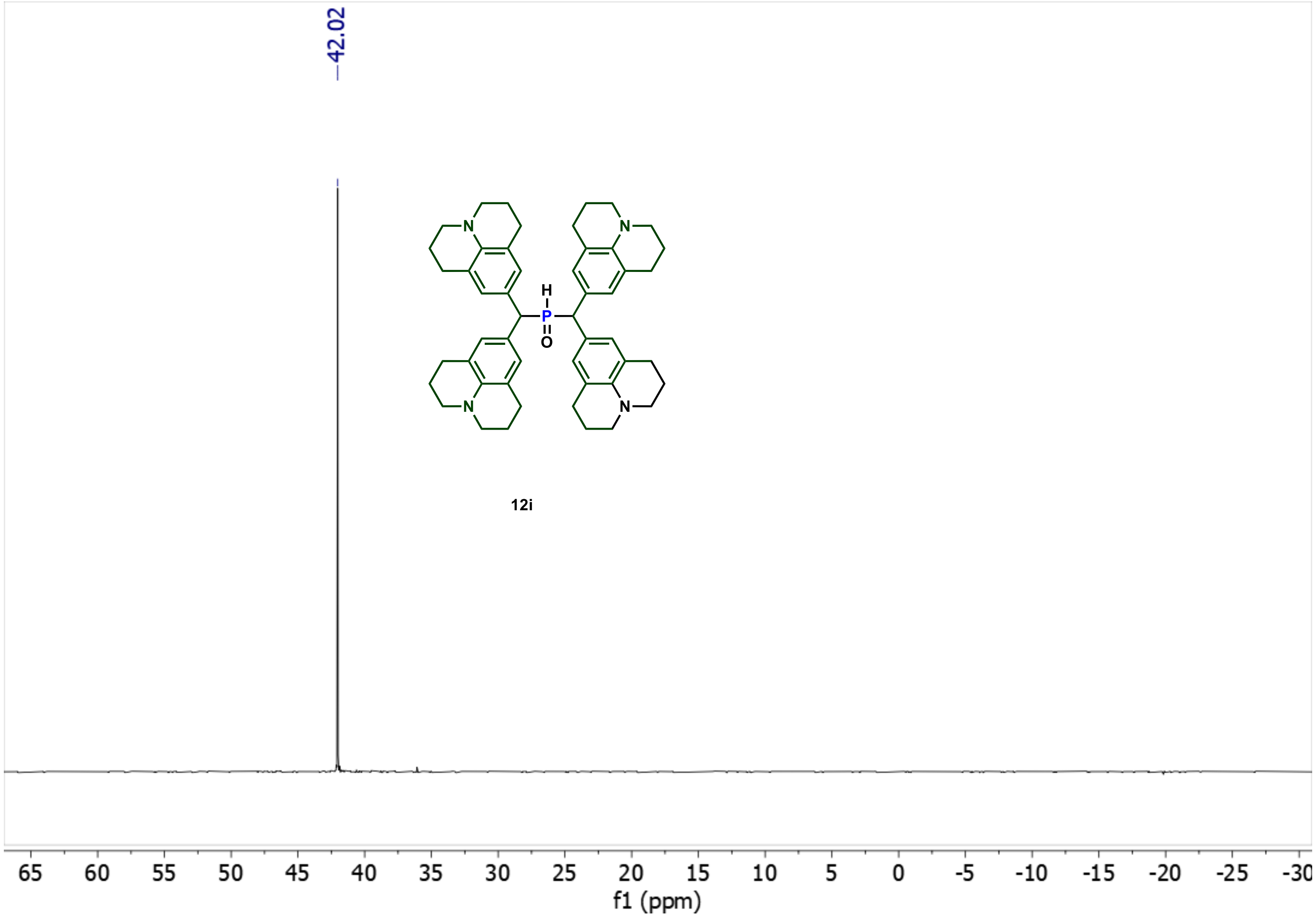

Figure 43. $^{31}P\{H\}$ NMR (243 MHz, Methylene Chloride-d_2) of **12i**

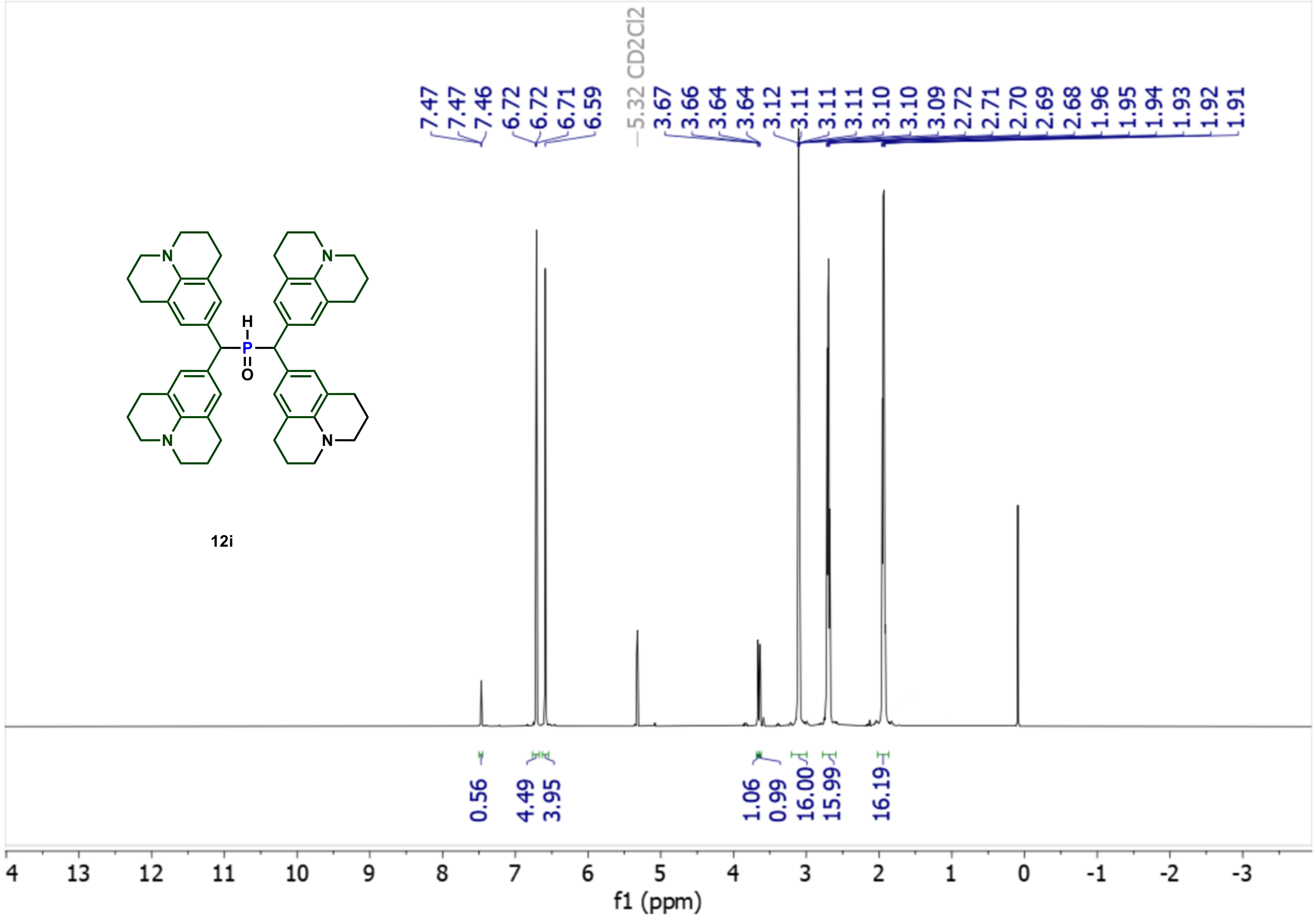

Figure 44. ^{1}H NMR (600 MHz, Methylene Chloride-d_2) of **12i**

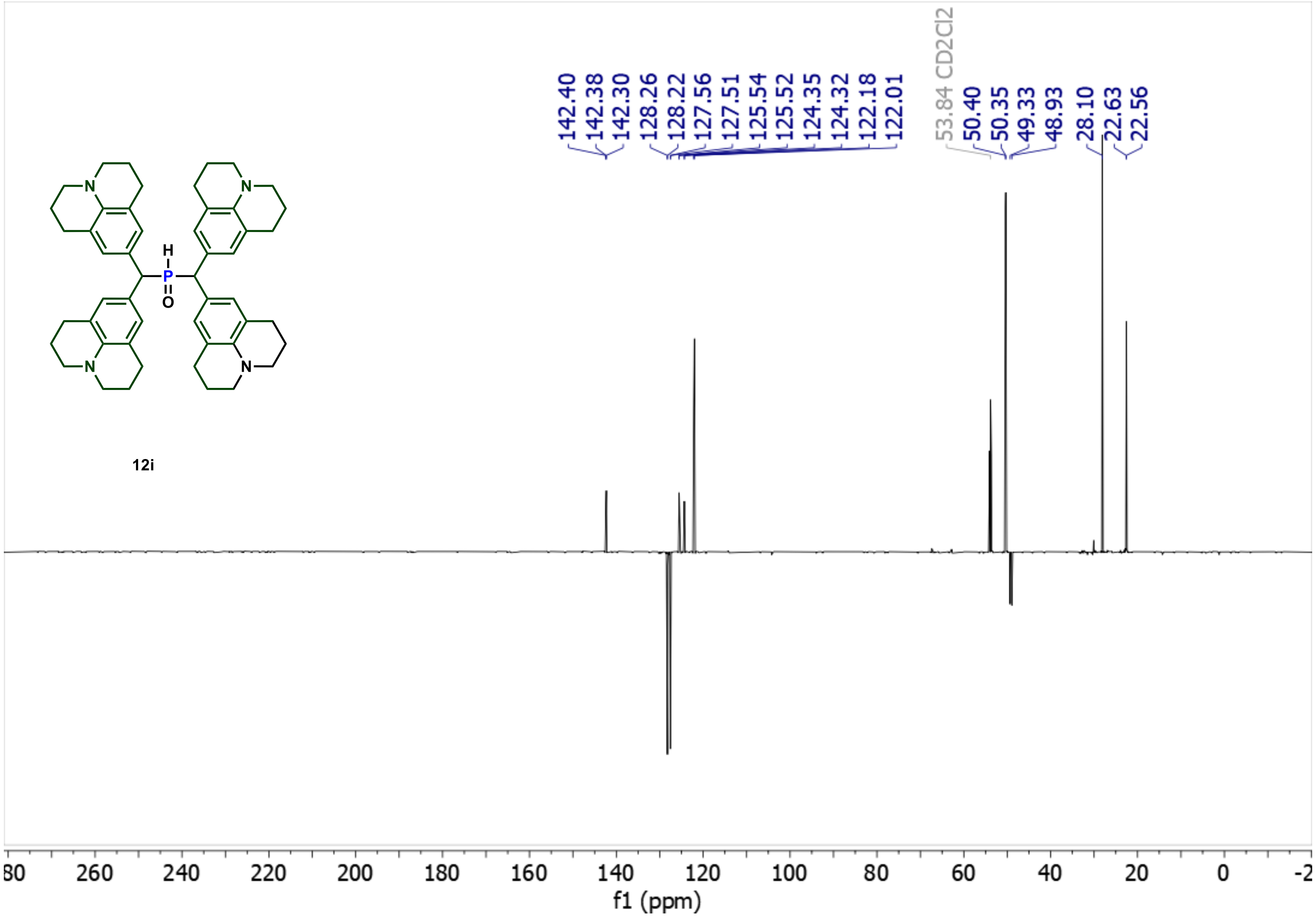

Figure 45. JMOD ^{13}C NMR (151 MHz, Methylene Chloride-d_2) of **12i**

Single Mass Analysis
Tolerance = 5.0 PPM / DBE: min = -1.5, max = 80.0
Element prediction: Off

Monoisotopic Mass, Odd and Even Electron Ions
306 formula(e) evaluated with 3 results within limits (all results (up to 1000) for each mass)
Elements Used:
C: 0-100 H: 0-100 N: 0-5 O: 0-5 P: 1-1

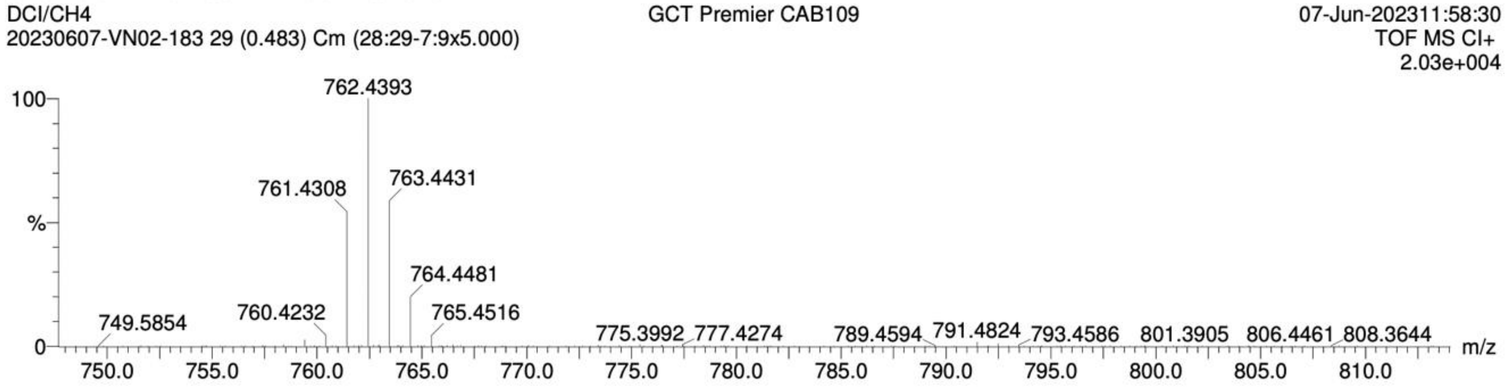

Minimum:				-1.5		
Maximum:		1.4	5.0	80.0		
Mass	Calc. Mass	mDa	PPM	DBE	i-FIT	Formula
762.4393	762.4427	-3.4	-4.5	24.0	43.4	C50 H59 N4 O P
	762.4413	-2.0	-2.6	19.0	60.6	C49 H63 O5 P
	762.4400	-0.7	-0.9	19.5	96.2	C47 H61 N3 O4 P

Figure 46. HRMS (DCI) for **12i**

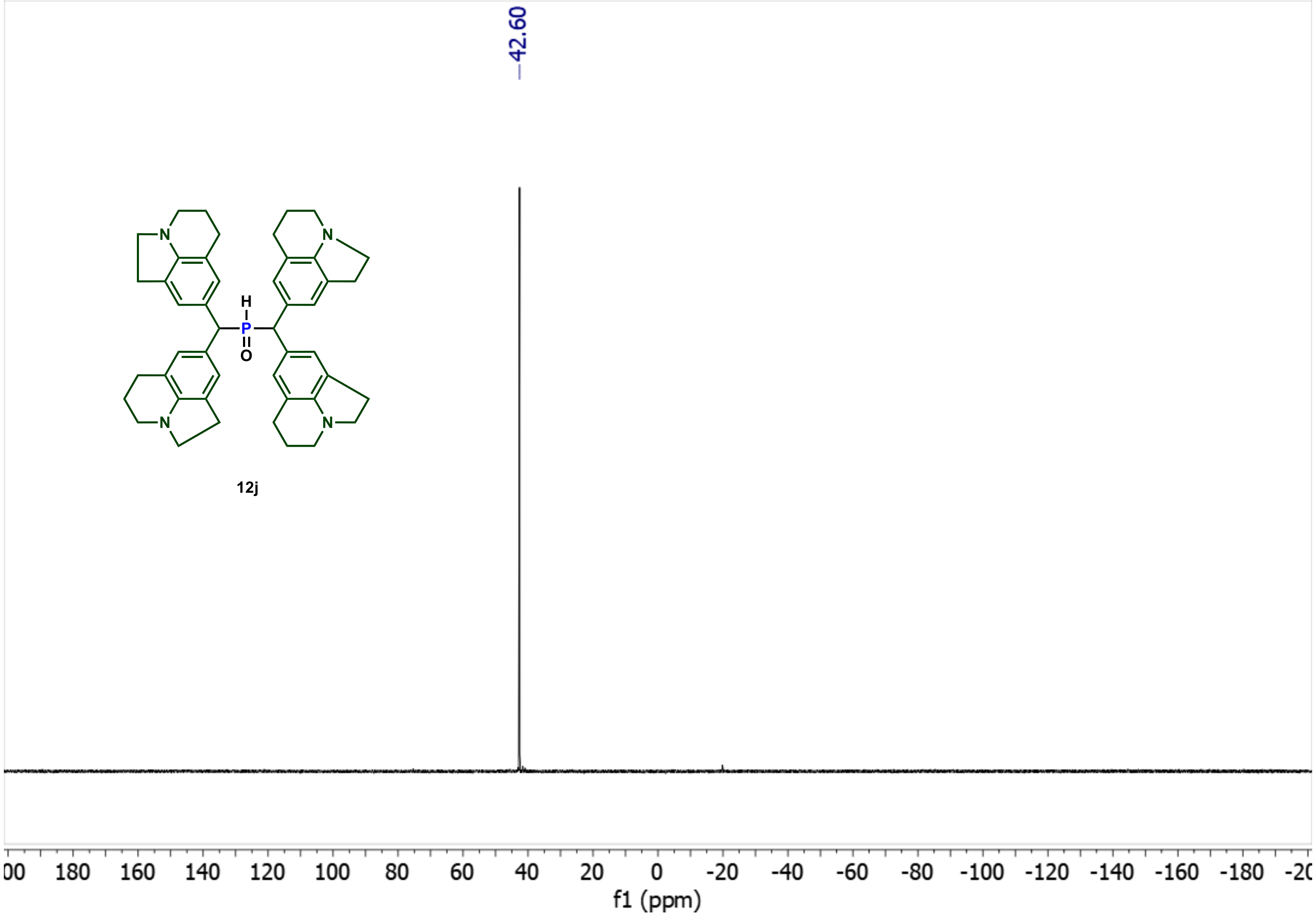

Figure 47. $^{31}P\{H\}$ NMR (202 MHz, Methylene Chloride-d_2) of **12j**

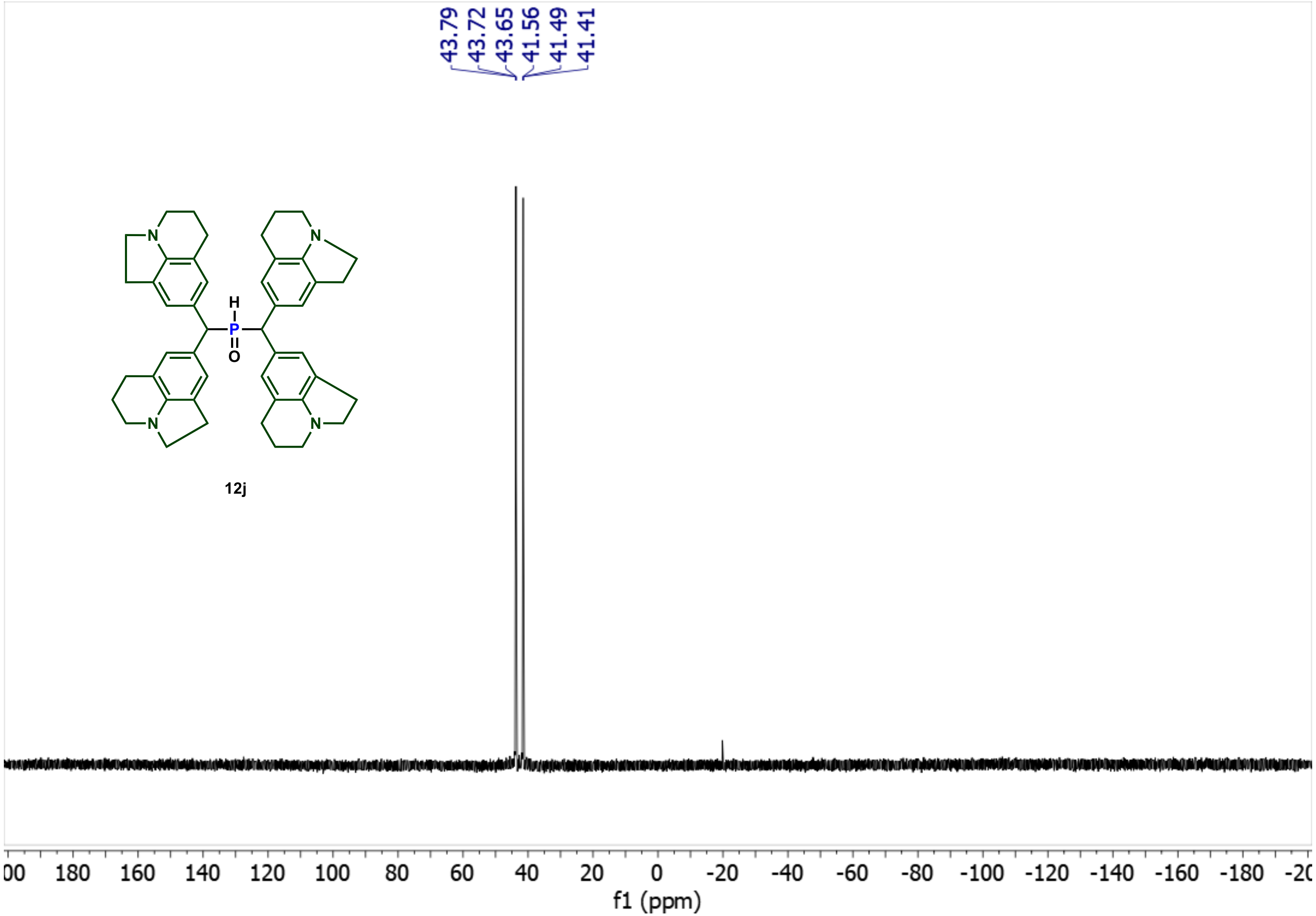

Figure 48. ^{31}P NMR (202 MHz, Methylene Chloride-d_2) of **12j**

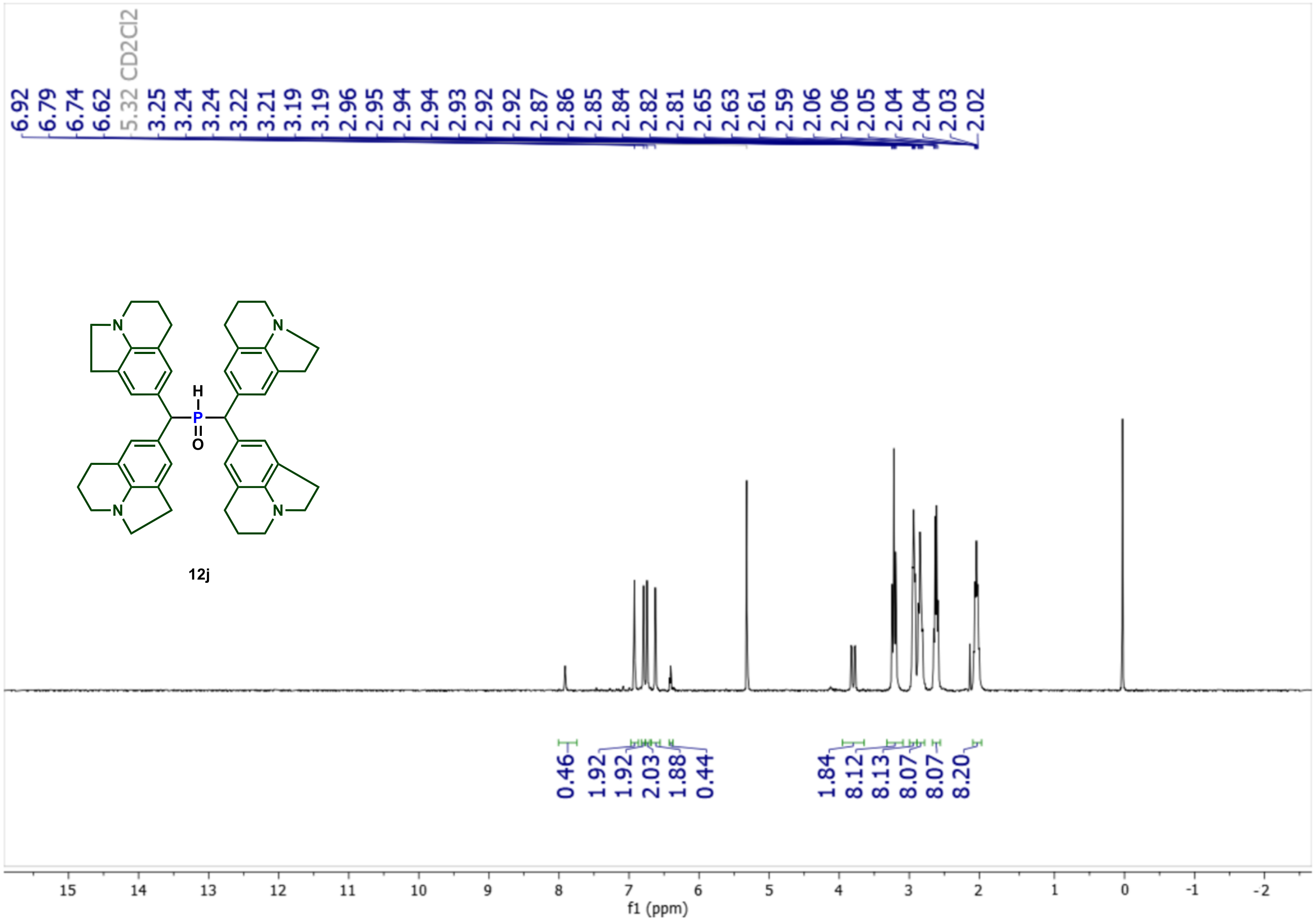

Figure 49. ^{1}H NMR (300 MHz, Methylene Chloride-d_2) of **12j**

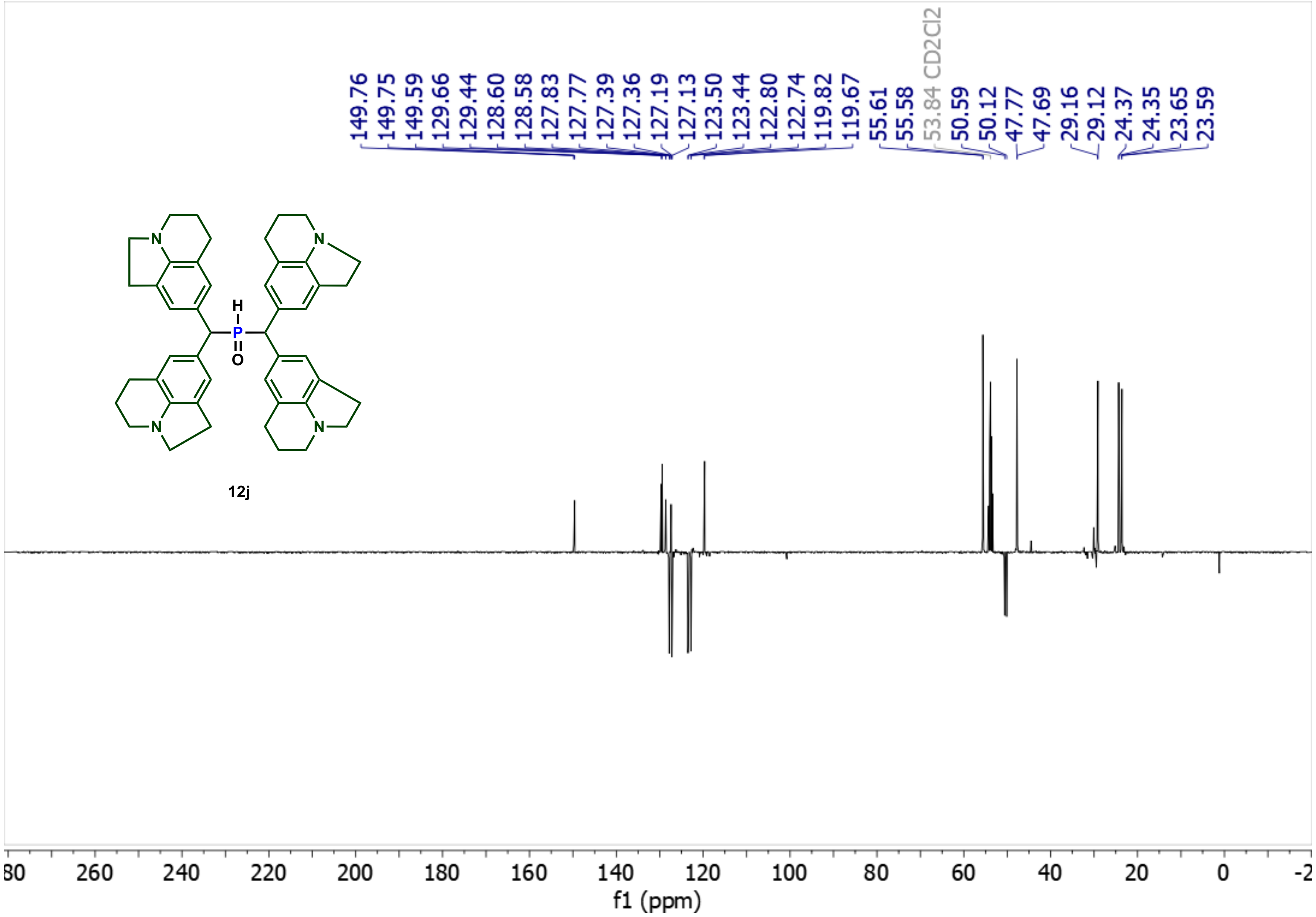

Figure 50. JMOD ^{13}C NMR (126 MHz, Methylene Chloride-d_2) of **12j**

Single Mass Analysis
Tolerance = 5.0 PPM / DBE: min = -1.5, max = 80.0
Element prediction: Off

Monoisotopic Mass, Odd and Even Electron Ions
300 formula(e) evaluated with 4 results within limits (all results (up to 1000) for each mass)
Elements Used:
C: 0-100 H: 0-100 N: 0-5 O: 0-5 P: 1-1

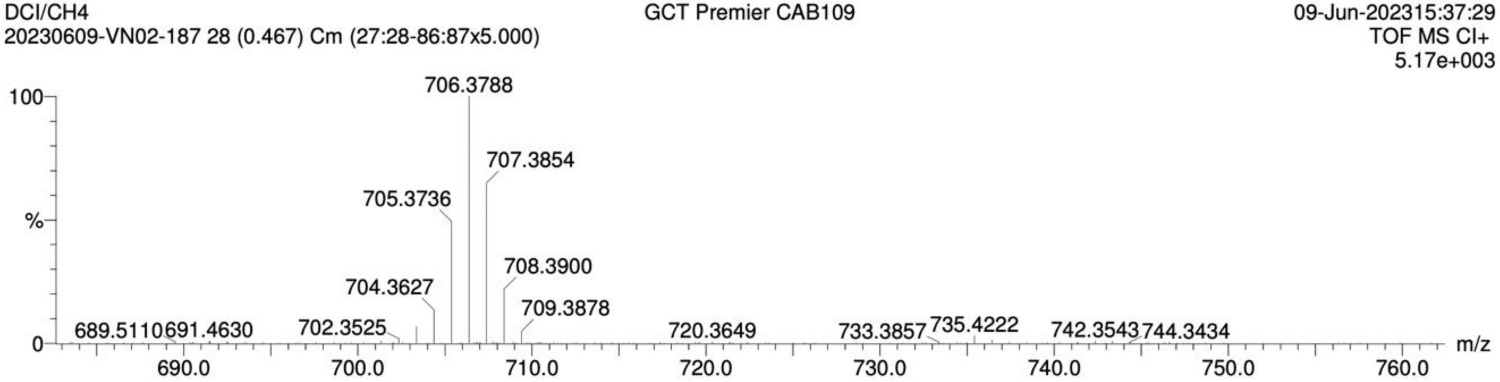

		mDa	PPM	DBE	i-FIT	Formula
Minimum:				-1.5		
Maximum:		1.4	5.0	80.0		
Mass	Calc. Mass	mDa	PPM	DBE	i-FIT	Formula
706.3788	706.3787	0.1	0.1	19.0	106.8	C45 H55 O5 P
	706.3801	-1.3	-1.8	24.0	84.3	C46 H51 N4 O P
	706.3774	1.4	2.0	19.5	130.6	C43 H53 N3 O4 P
	706.3814	-2.6	-3.7	23.5	65.6	C48 H53 N O2 P

Figure 51. HRMS (DCI) for **12j**

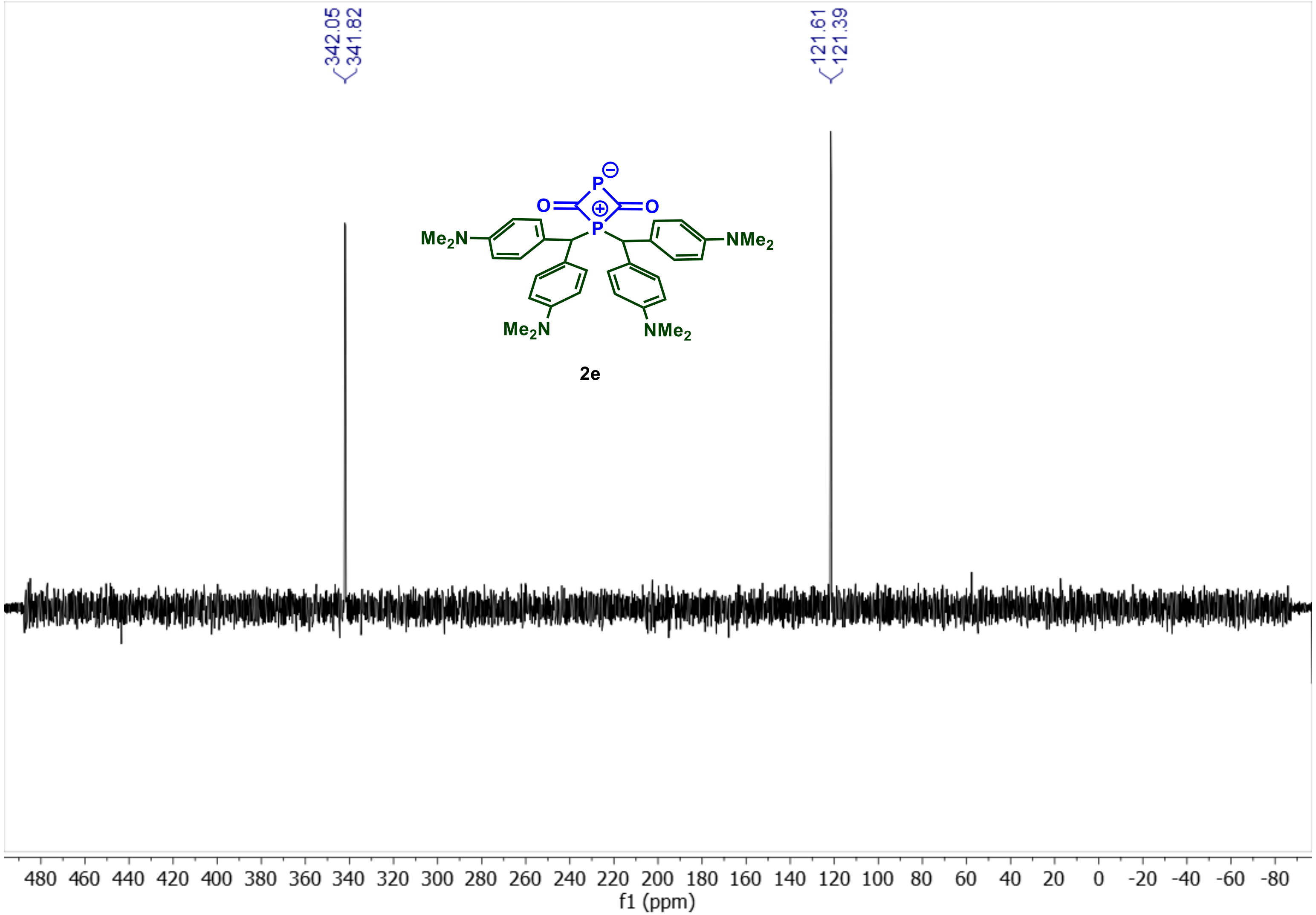

Figure 52. $^{31}P\{H\}$ NMR (162 MHz, THF-d_8 and CD_2Cl_2) at -60°C of **2e**

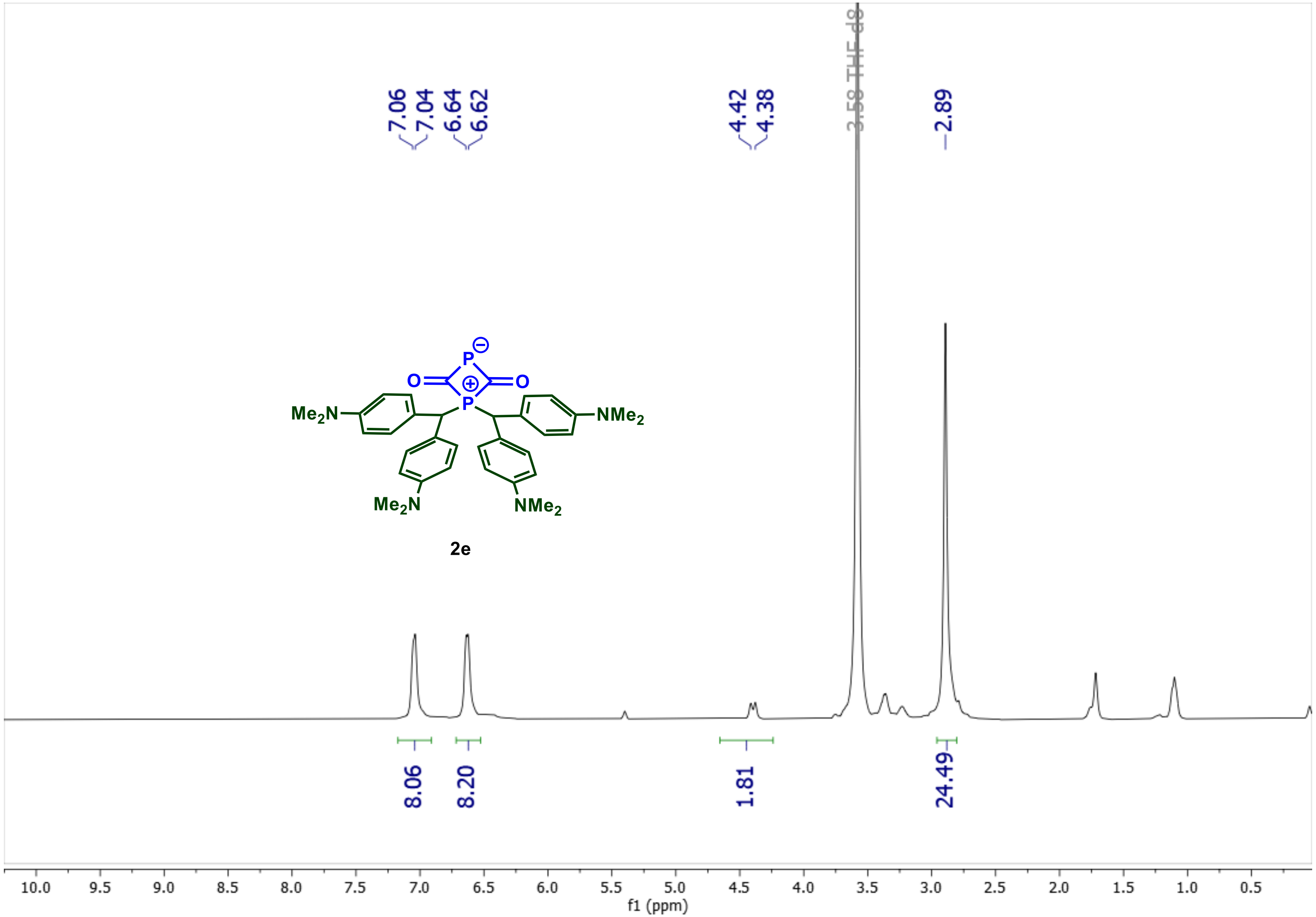

Figure 53. ^{1}H NMR (400 MHz, THF-d_8 and CD_2Cl_2) at -60ºC of **2e**

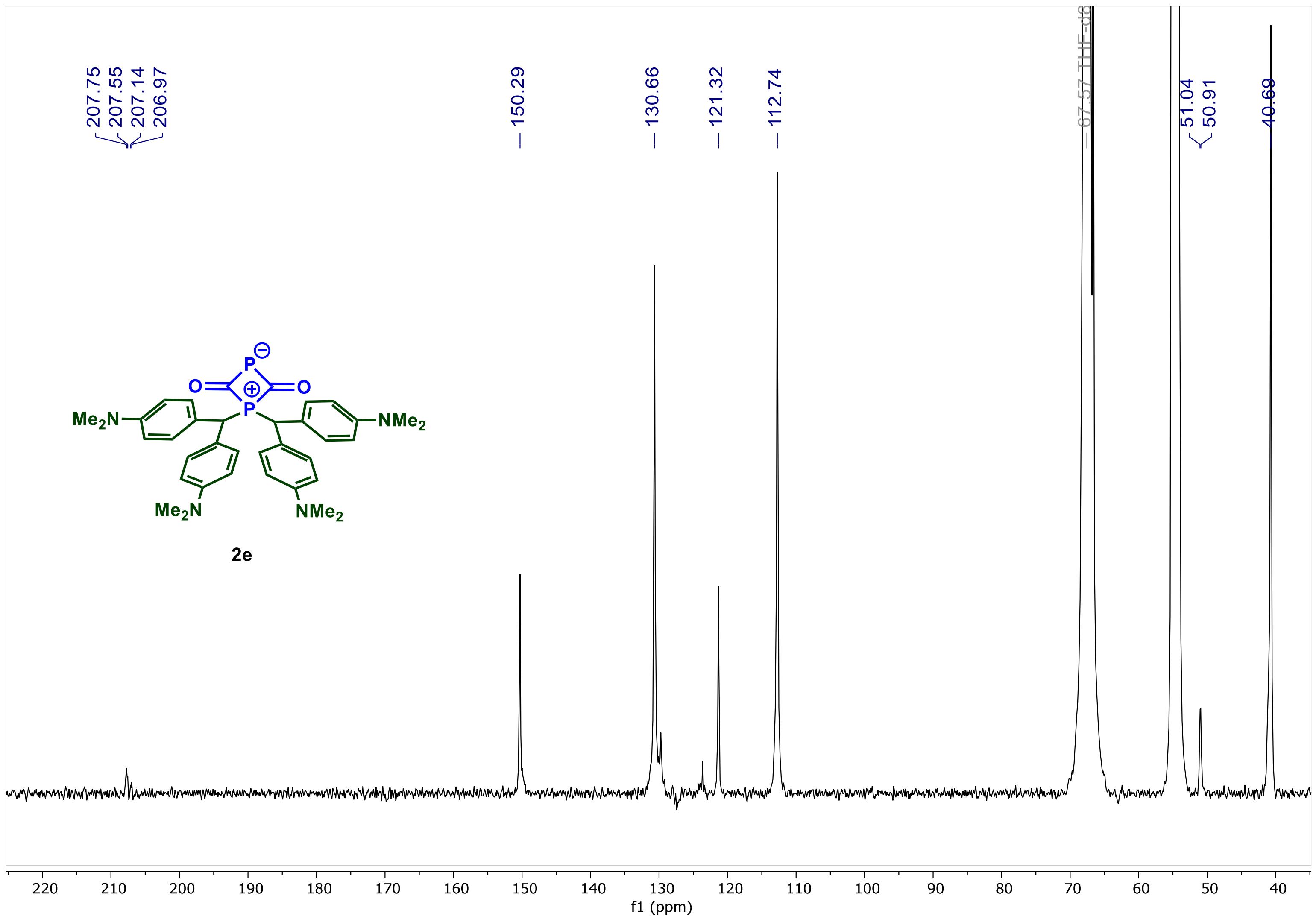

Figure 54. JMOD ^{13}C NMR (101 MHz, THF-d_8 and CD_2Cl_2) at -60°C of **2e**

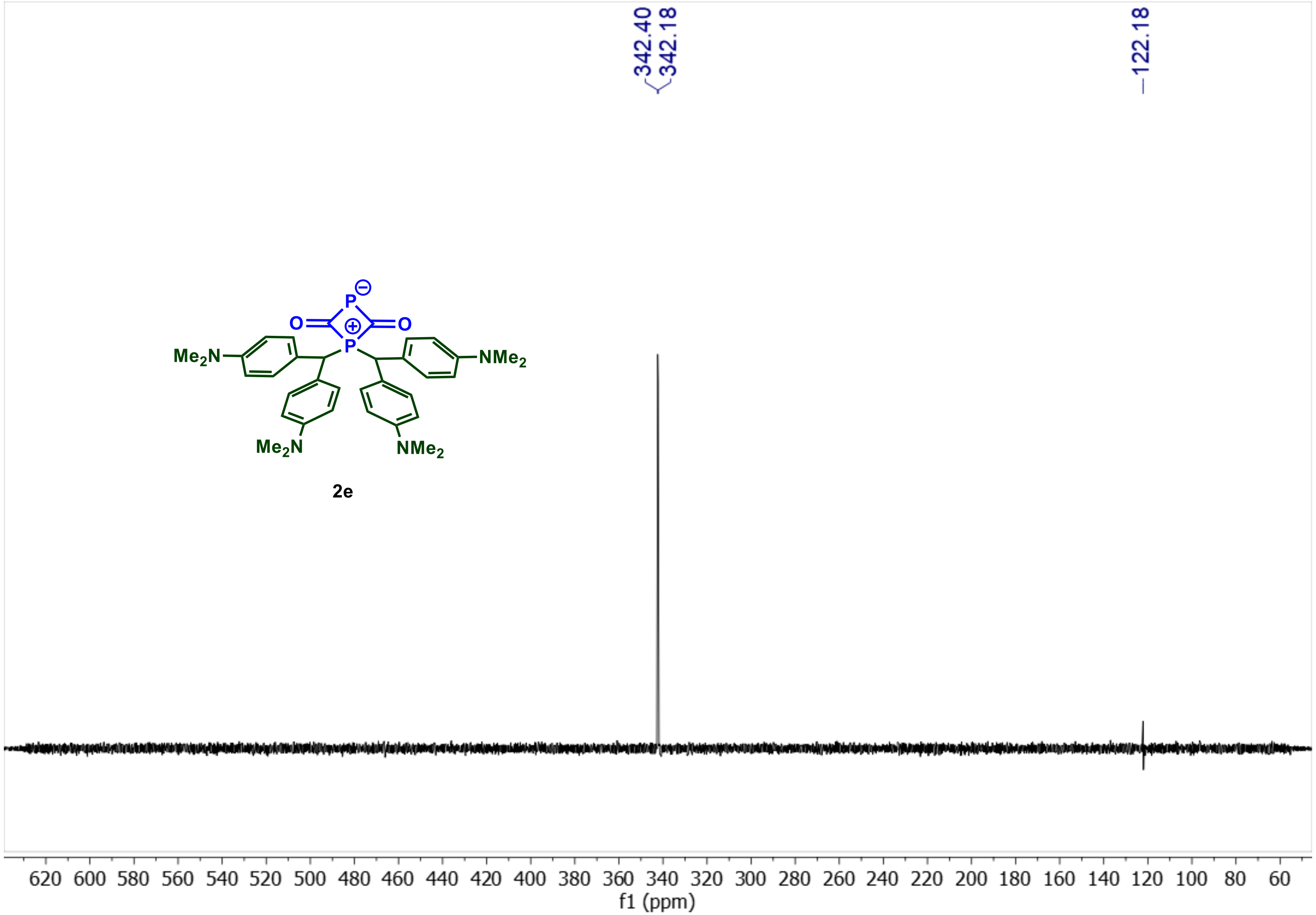

Figure 55. COSY NMR Selective 1D ^{31}P – ^{31}P decoupled (101 MHz, THF-d_8 and CD_2Cl_2) at -60°C of **2e**

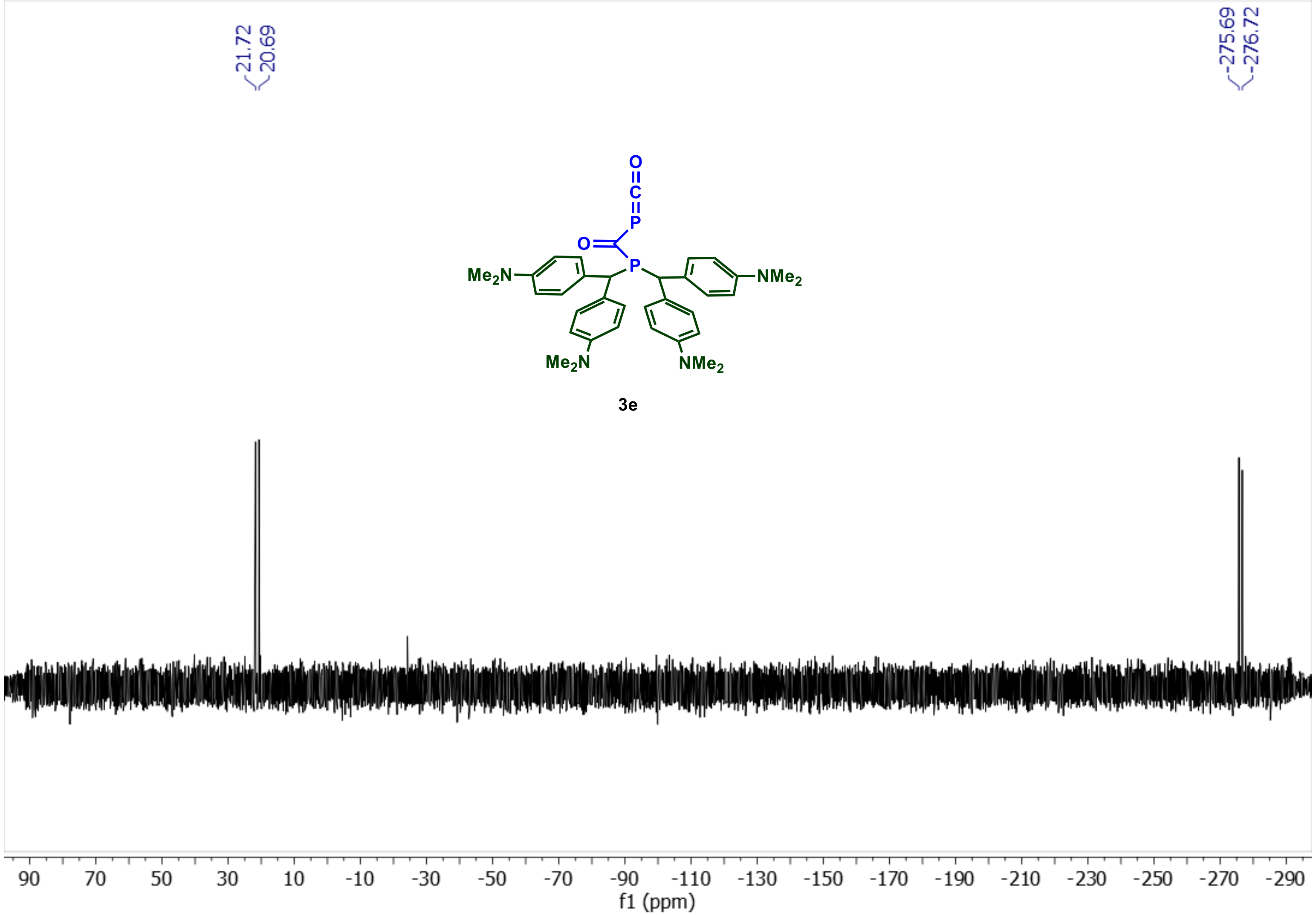

Figure 56. $^{31}P\{H\}$ NMR (162 MHz, THF-d_8 and CD_2Cl_2) at -20^oC of **3e**

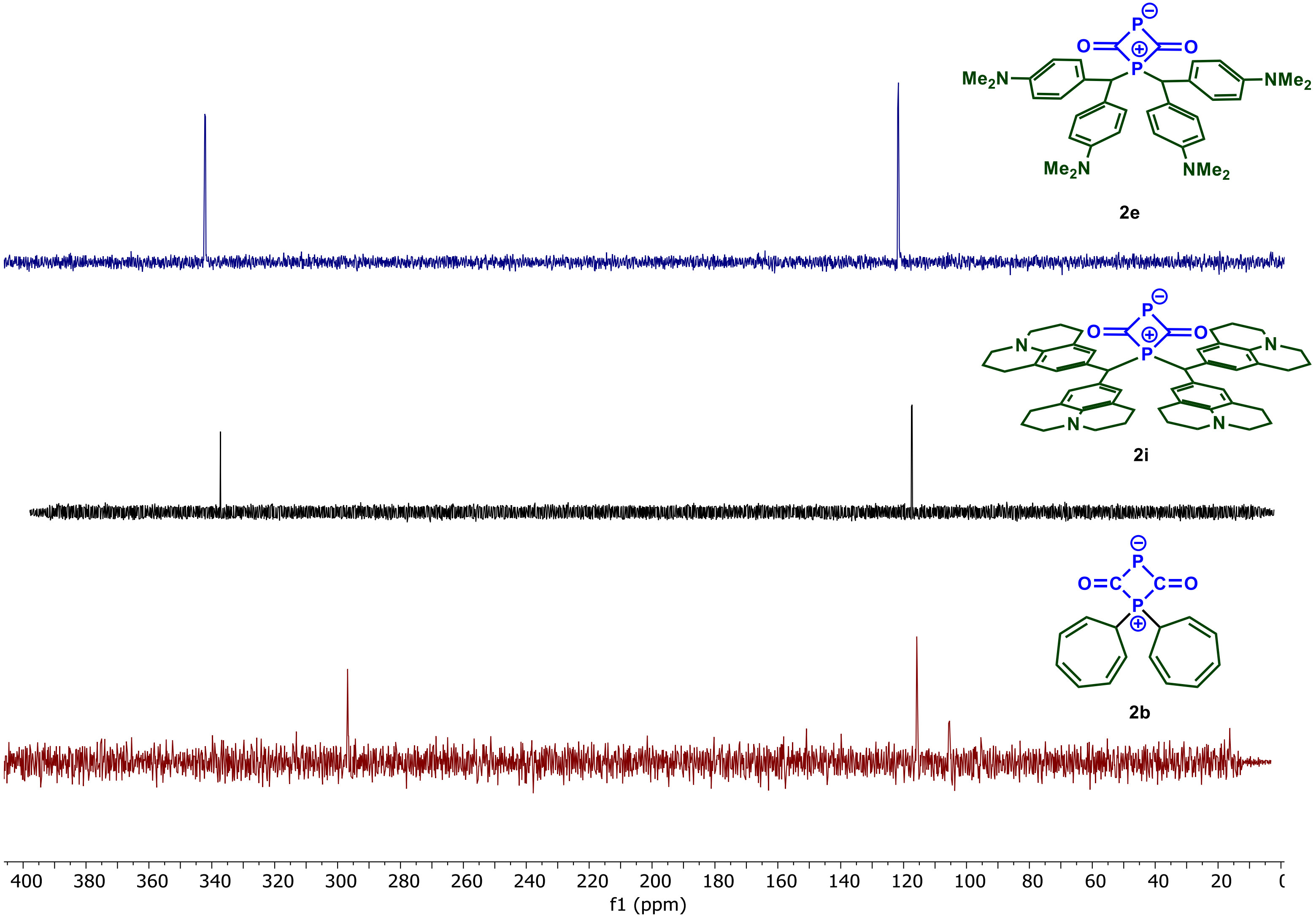

Figure 57. Stacked $^{31}P\{H\}$ NMR (162 MHz, THF-d_8 and CD_2Cl_2) at -60°C of **2e**, **2i** and **2b**

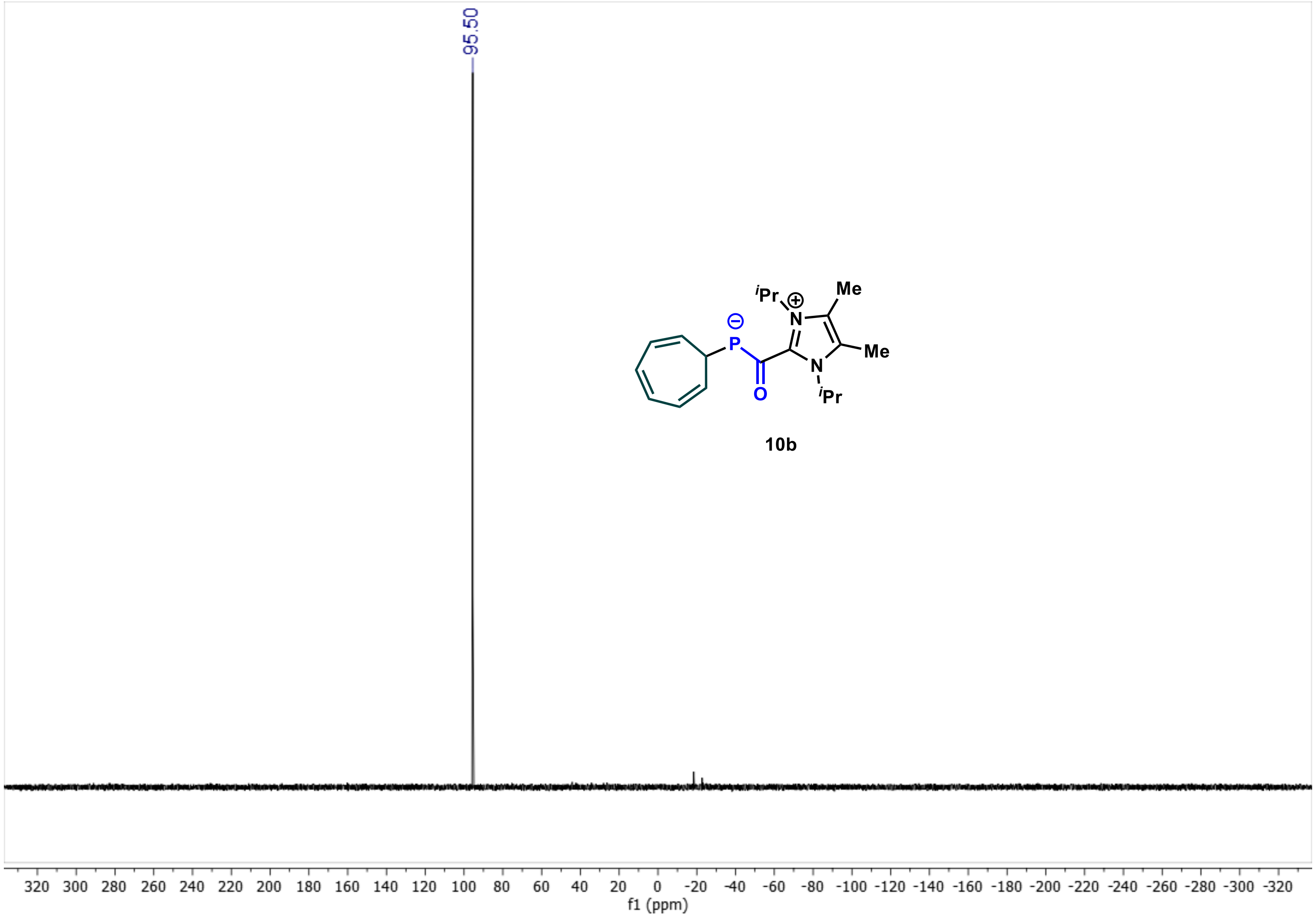

Figure 58. $^{31}P\{H\}$ NMR (202 MHz, Methylene Chloride-d_2) of **10b**

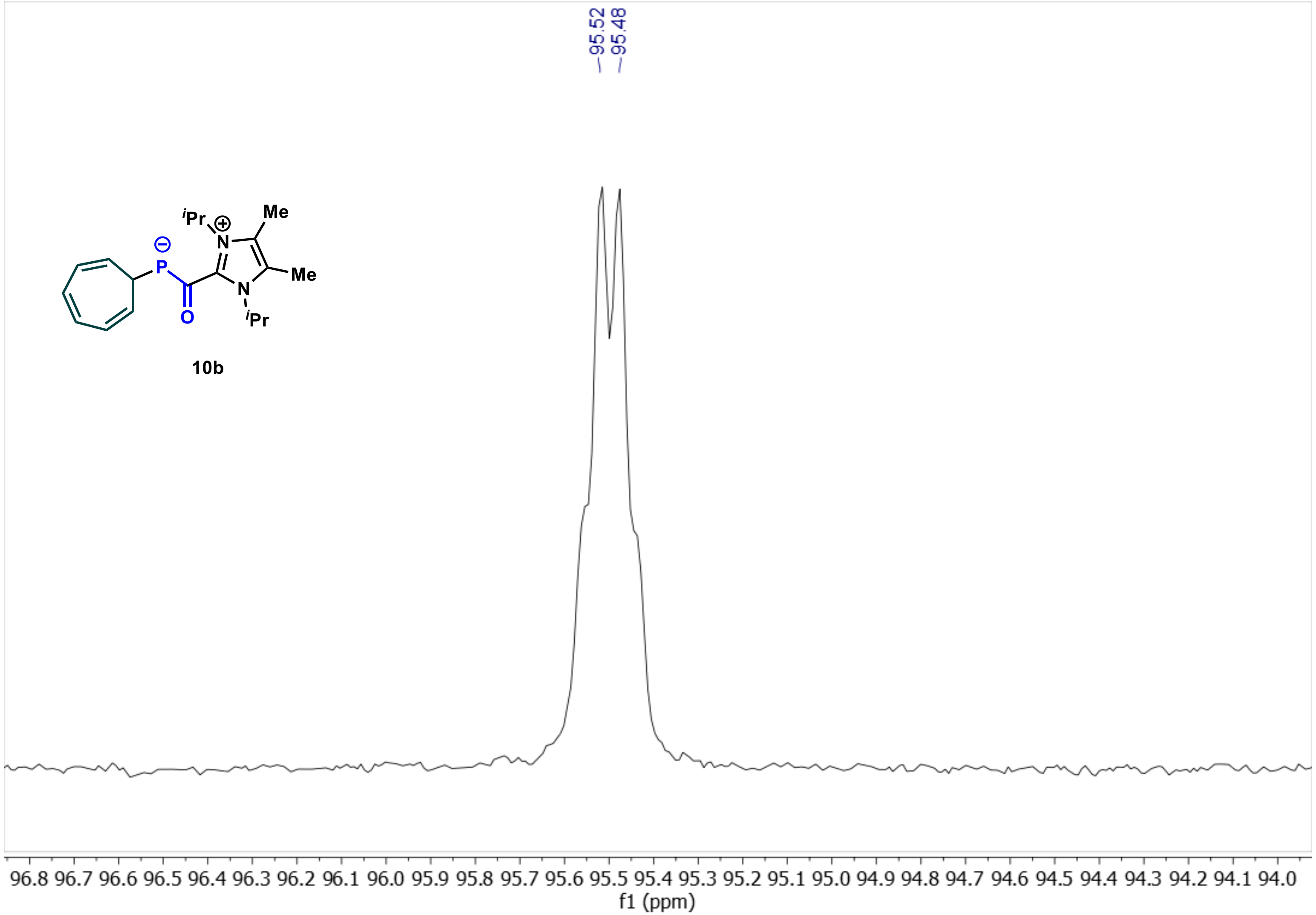

Figure 59. ^{31}P NMR (202 MHz, Methylene Chloride-d_2) of **10b**

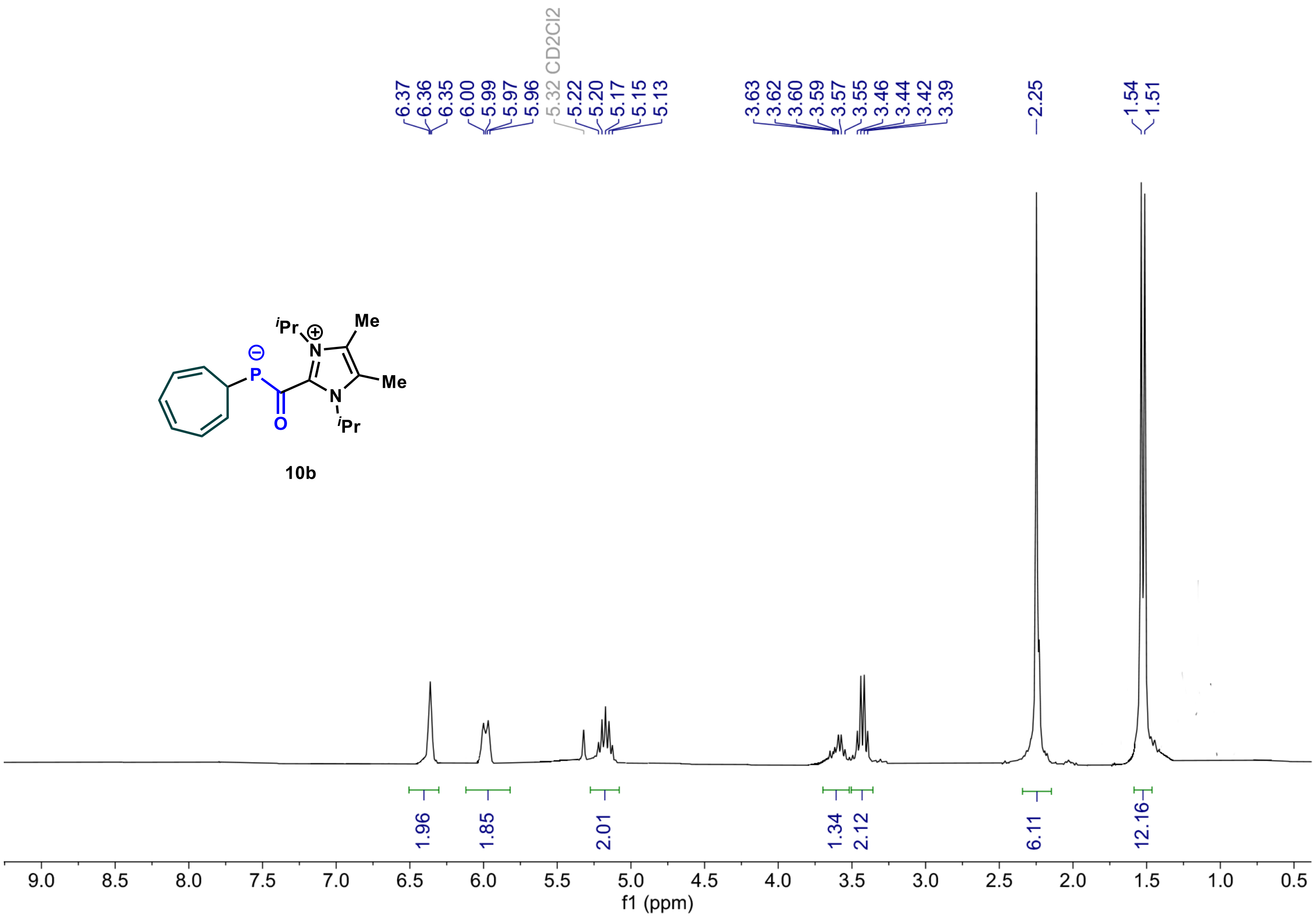

Figure 60. ^{1}H NMR (300 MHz, Methylene Chloride-d_2) of **10b**

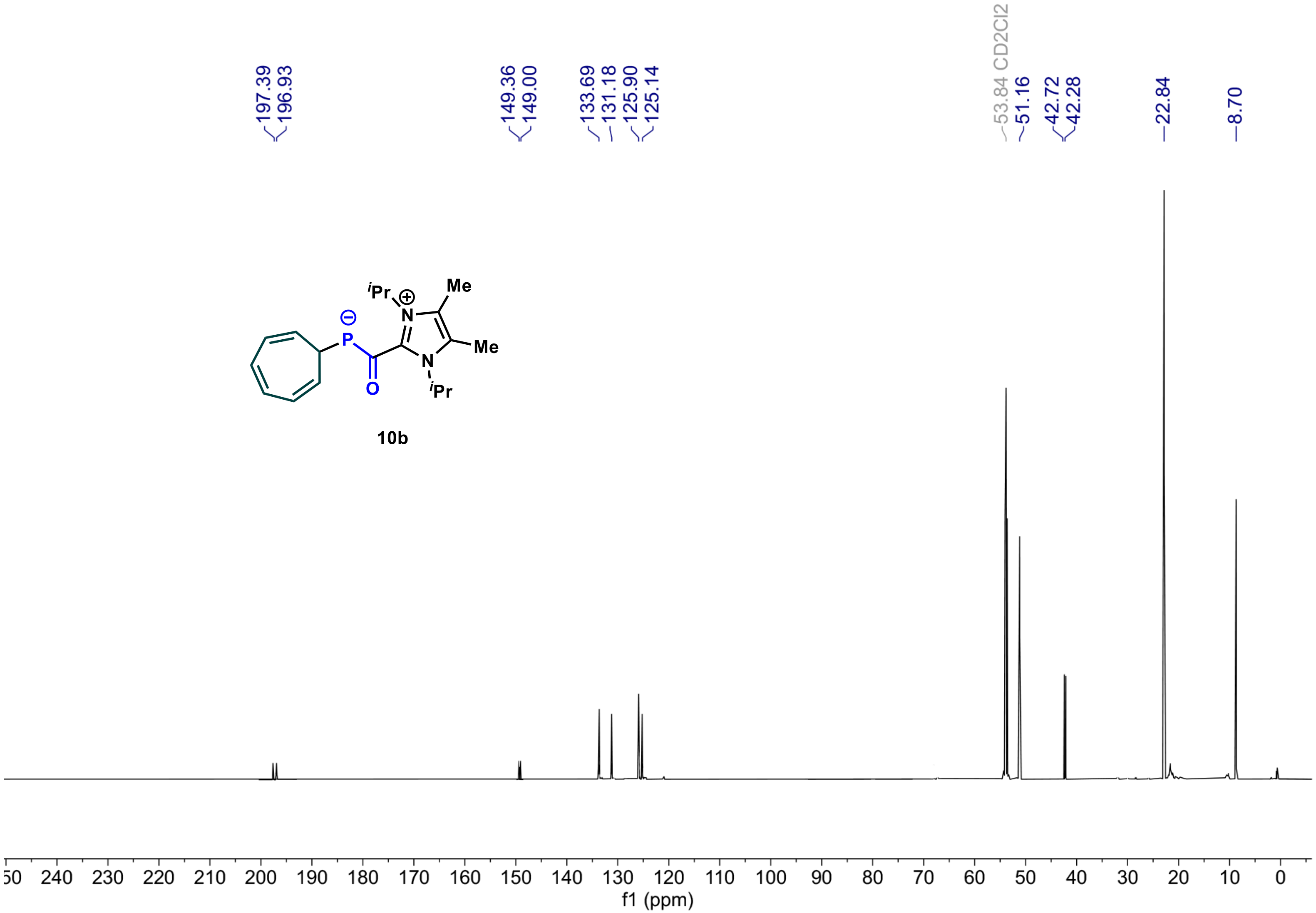

Figure 61. ^{13}C NMR (126 MHz, Methylene Chloride-d_2) of **10b**

Elemental Composition Report

Single Mass Analysis

Tolerance = 5.0 PPM / DBE: min = -1.5, max = 80.0
Element prediction: Off

Monoisotopic Mass, Odd and Even Electron Ions
285 formula(e) evaluated with 1 results within limits (all results (up to 1000) for each mass)
Elements Used:
C: 0-100 H: 0-100 N: 0-5 O: 0-5 P: 0-1

DCI-CH4 GCT Premier CAB109
20240703-VN103 22 (0.367) Cm (19:26-98:105x5.000)

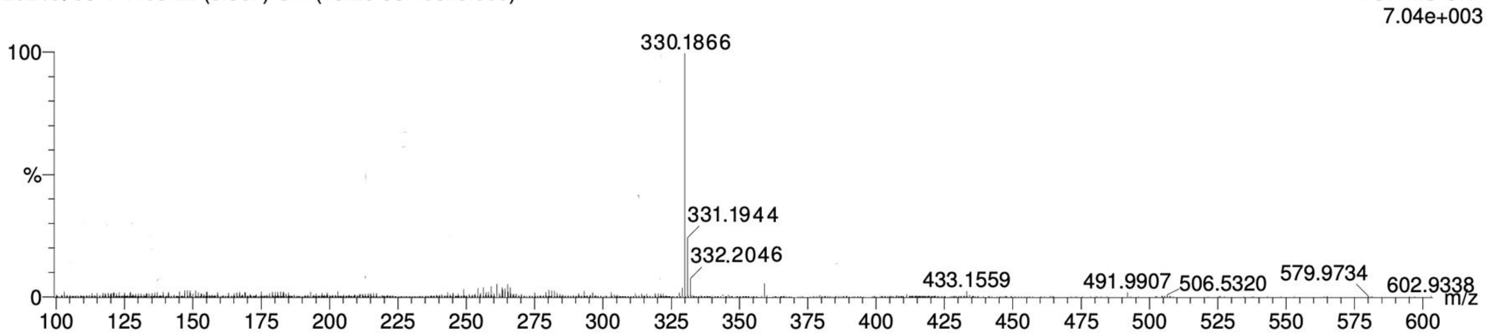

Minimum:				-1.5		
Maximum:		1.4	5.0	80.0		
Mass	Calc. Mass	mDa	PPM	DBE	i-FIT	Formula
330.1866	330.1861	0.5	1.6	11.5	114.5	C19 H27 N2 O P

Figure 62. HRMS (DCI) for **10b**

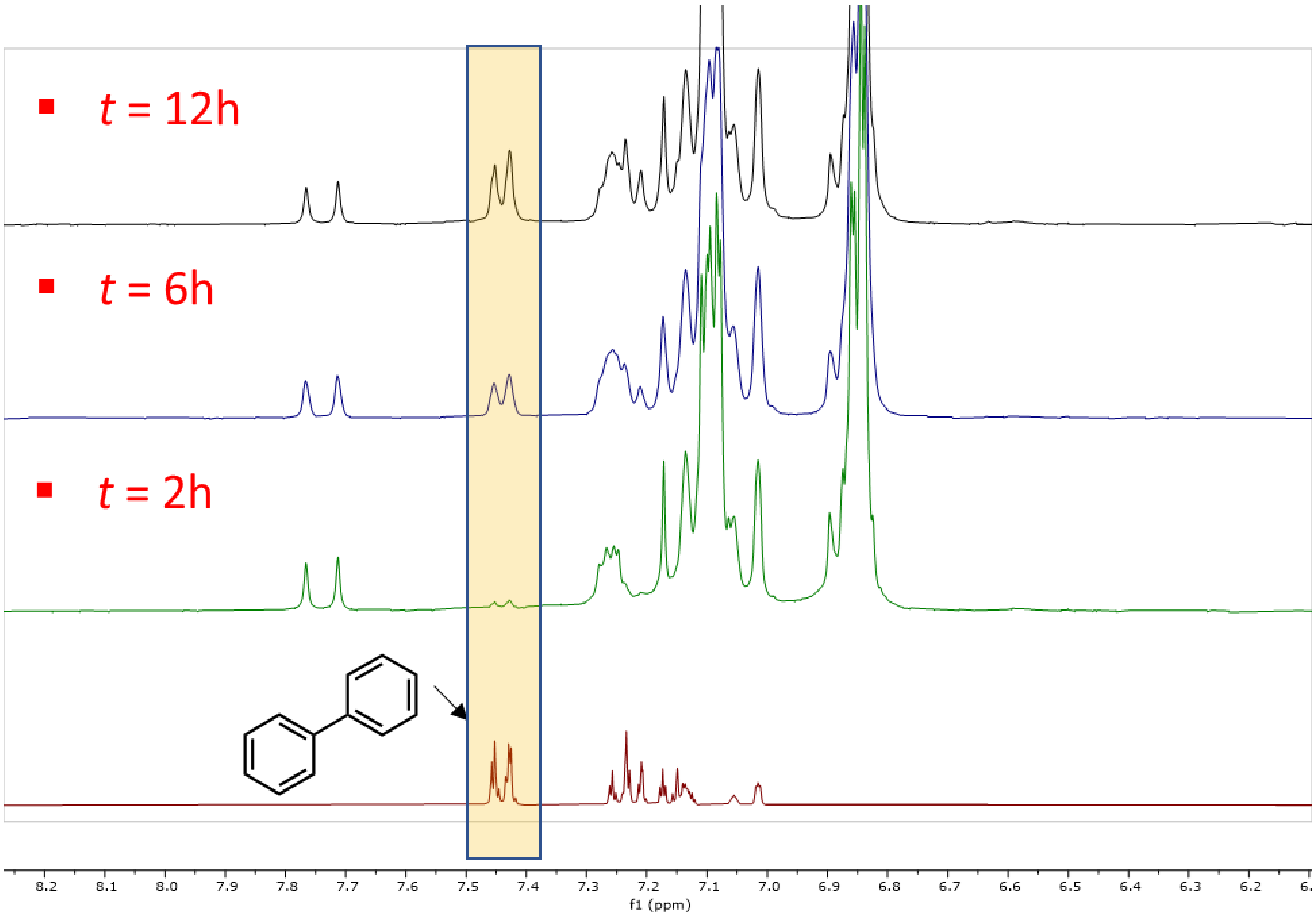

Figure 63. NMR studies of reaction between chlorobenzene and phenylboronic acid using systeme catalytic [Pd]/Phosphine 2 mol %

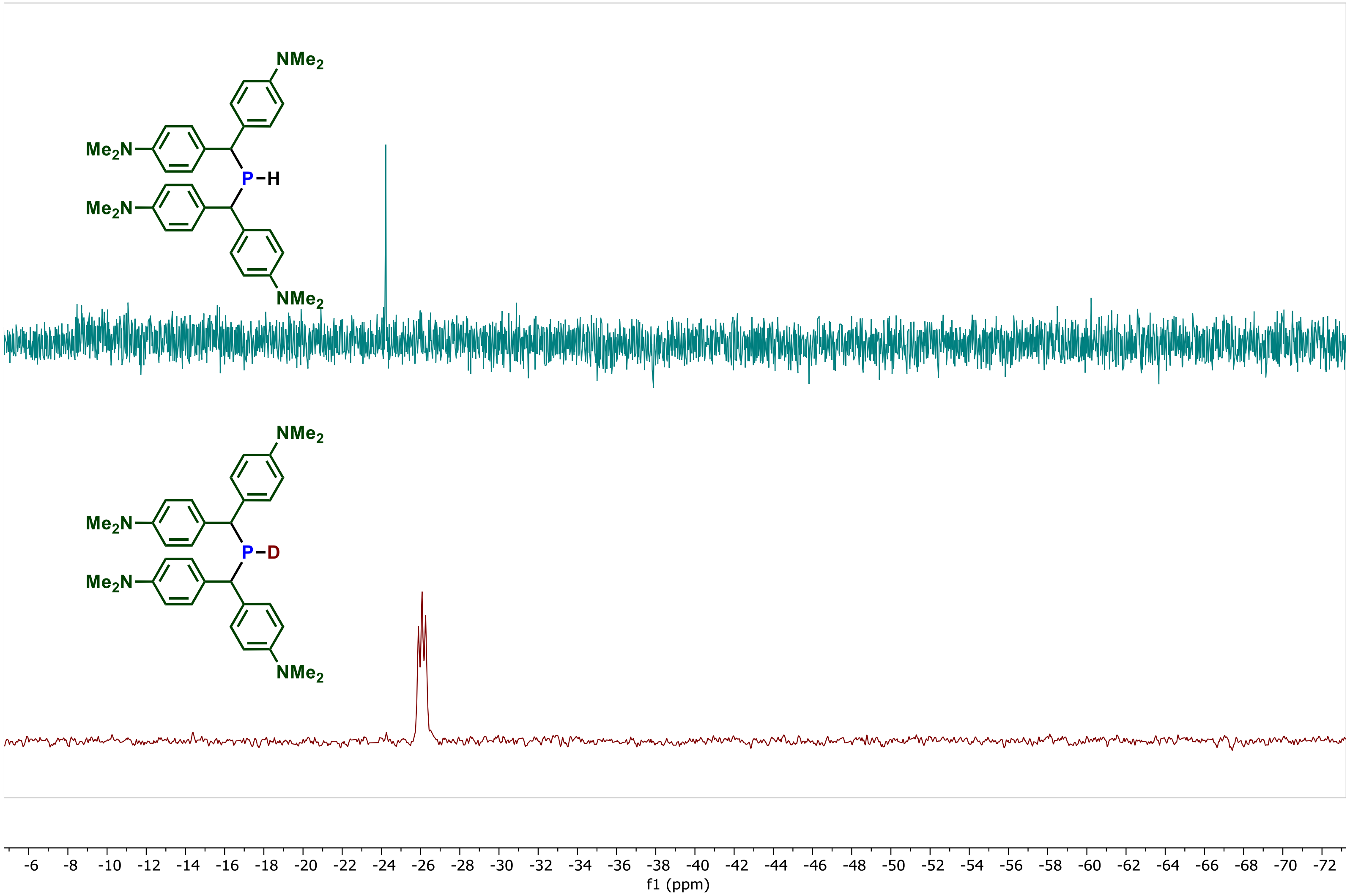

Figure 64. Comparison of $^{31}P[^{1}H]$ NMR of experiment in presence of D_2O (bottom) and without D_2O (top)

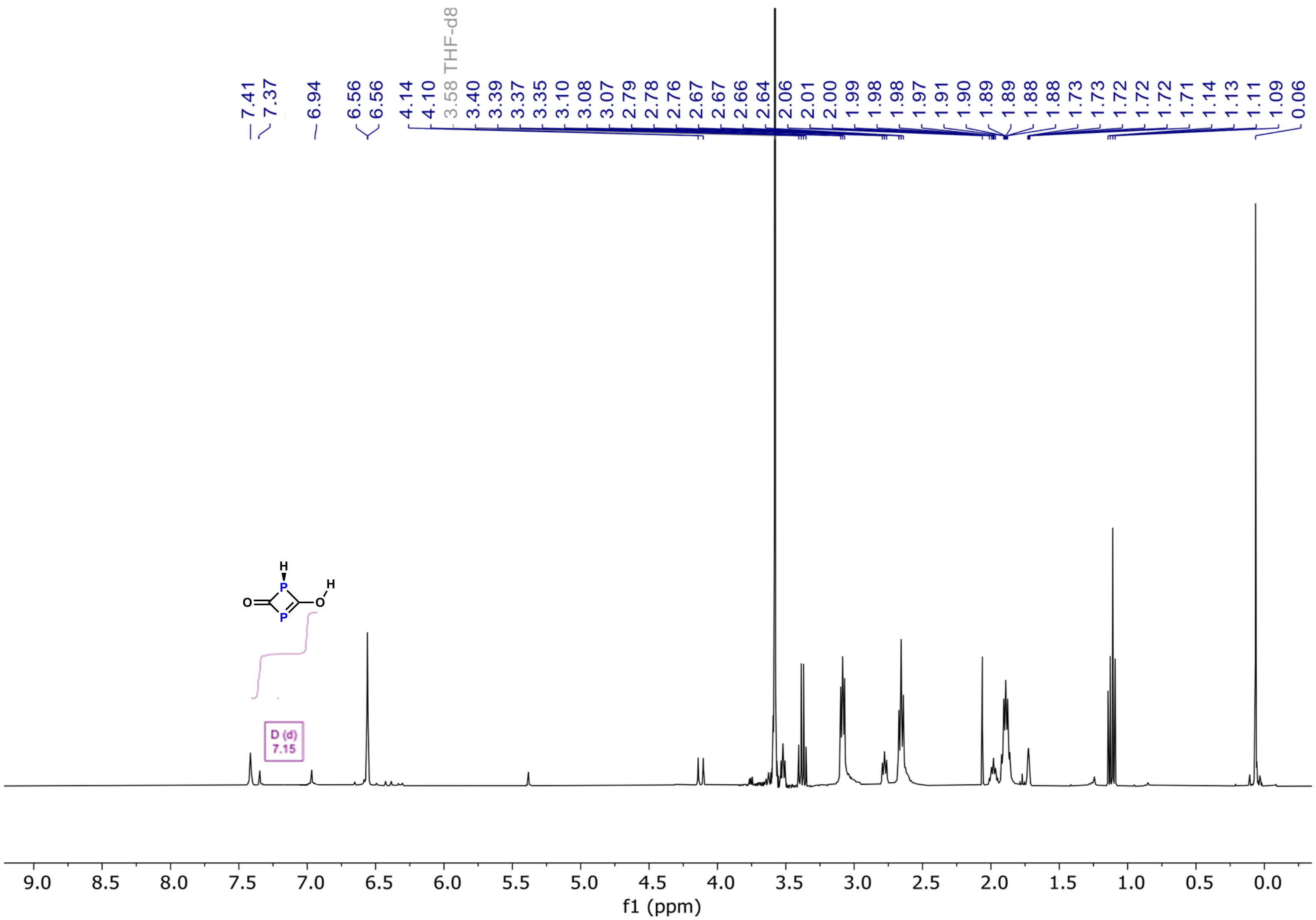

Figure 65. ^{1}H NMR (400 MHz, THF-d_8 and CD_2Cl_2) at -60°C of **2i, 3i** and dimer of HPCO

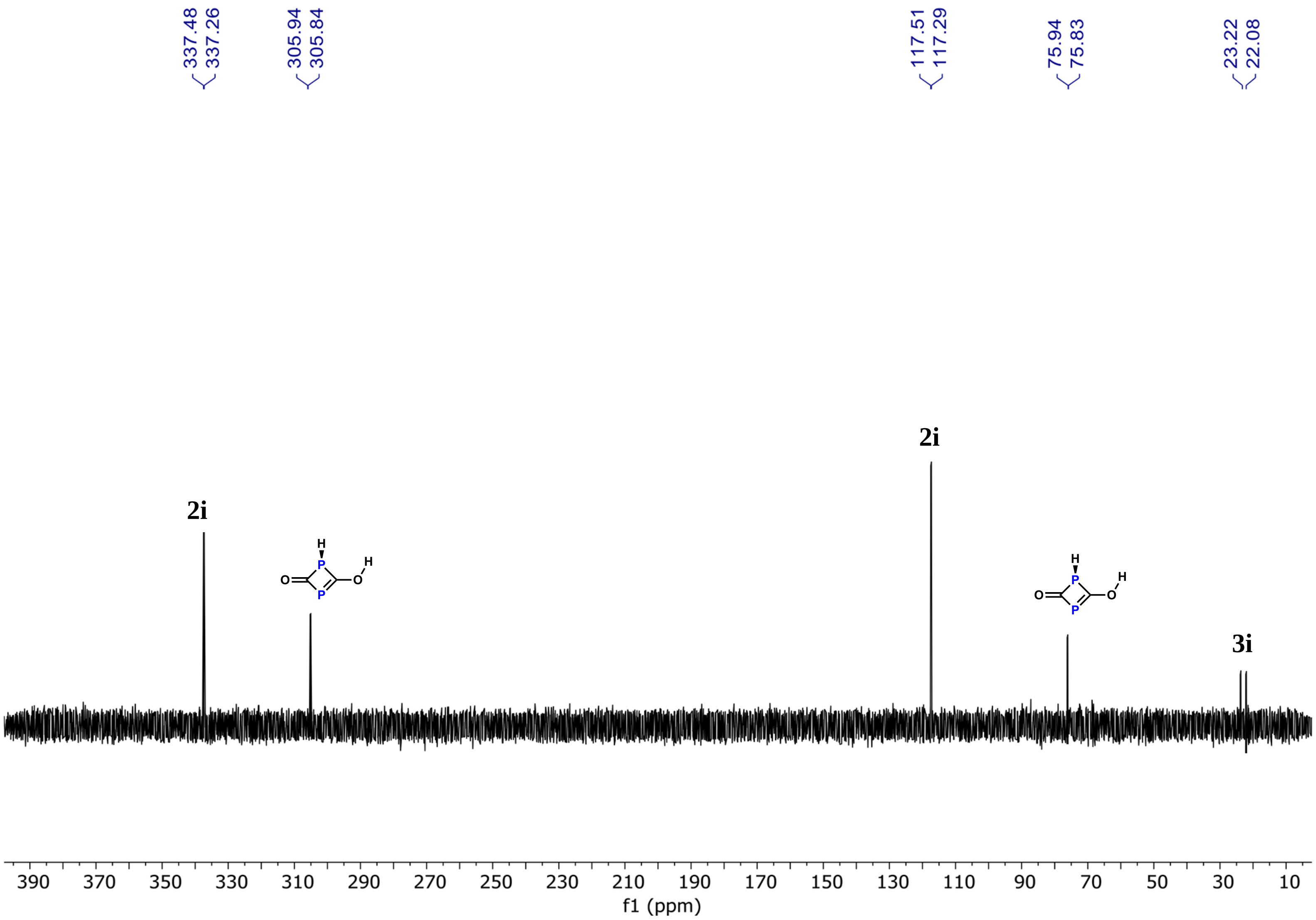

Figure 66. $^{31}P\{H\}$ NMR (162 MHz, THF-d_8 and CD_2Cl_2) at -60°C of **2i, 3i** and dimer of HPCO
